# Basalt-Hosted Microbial Communities in the Subsurface of the Young Volcanic Island of Surtsey, Iceland

**DOI:** 10.3389/fmicb.2021.728977

**Published:** 2021-09-29

**Authors:** Pauline Bergsten, Pauline Vannier, Alexandra María Klonowski, Stephen Knobloch, Magnús Tumi Gudmundsson, Marie Dolores Jackson, Viggó Thor Marteinsson

**Affiliations:** ^1^Exploration & Utilization of Genetic Resources, Matís, Reykjavík, Iceland; ^2^Faculty of Life and Environmental Sciences, University of Iceland, Reykjavík, Iceland; ^3^Nordvulk, Institute of Earth Sciences, University of Iceland, Reykjavík, Iceland; ^4^Department of Geology and Geophysics, University of Utah, Salt Lake City, UT, United States; ^5^Faculty of Food Science and Nutrition, University of Iceland, Reykjavík, Iceland

**Keywords:** 16S rRNA gene amplicon sequencing, bacterial and archaeal communities, microbial diversity, extreme environment, subsurface, oceanic basaltic crust, Iceland

## Abstract

The island of Surtsey was formed in 1963–1967 on the offshore Icelandic volcanic rift zone. It offers a unique opportunity to study the subsurface biosphere in newly formed oceanic crust and an associated hydrothermal-seawater system, whose maximum temperature is currently above 120°C at about 100m below surface. Here, we present new insights into the diversity, distribution, and abundance of microorganisms in the subsurface of the island, 50years after its creation. Samples, including basaltic tuff drill cores and associated fluids acquired at successive depths as well as surface fumes from fumaroles, were collected during expedition 5059 of the International Continental Scientific Drilling Program specifically designed to collect microbiological samples. Results of this microbial survey are investigated with 16S rRNA gene amplicon sequencing and scanning electron microscopy. To distinguish endemic microbial taxa of subsurface rocks from potential contaminants present in the drilling fluid, we use both methodological and computational strategies. Our 16S rRNA gene analysis results expose diverse and distinct microbial communities in the drill cores and the borehole fluid samples, which harbor thermophiles in high abundance. Whereas some taxonomic lineages detected across these habitats remain uncharacterized (e.g., Acetothermiia, Ammonifexales), our results highlight potential residents of the subsurface that could be identified at lower taxonomic rank such as *Thermaerobacter*, BRH-c8a (Desulfallas-Sporotomaculum), *Thioalkalimicrobium,* and *Sulfurospirillum*. Microscopy images reveal possible biotic structures attached to the basaltic substrate. Finally, microbial colonization of the newly formed basaltic crust and the metabolic potential are discussed on the basis of the data.

## Introduction

The subsurface biosphere, defined as an ecosystem encompassing regions beneath soils and sediments, occupies roughly twice the volume of the oceans and holds about 15% of the total biomass on Earth (Pedersen, 2000; Heberling et al., 2010; Bar-On et al., 2018; Orcutt et al., 2019). Recent estimates suggest that these zones (i.e., continental subsurface, subseafloor sediments, and oceanic crust) contain ~70% of all prokaryotic cells and possibly more than 80% of all bacterial and archaeal species (Magnabosco et al., 2018). This large biome has only recently become the focus of research studies (Gold, 1992), and most microbial surveys have, to date, focused on subseafloor sediments (Parkes et al., 2000; D’Hondt et al., 2004; Inagaki et al., 2015). The deep biosphere hosted in the basaltic ocean crust, on the contrary, has been understudied because of various challenges: the relative inaccessibility of the samples (drilling operation often required), the low biomass (high risk of external contamination), the presence of minerals which generates DNA binding and inhibitions (Direito et al., 2012; Lever et al., 2015), and the difficulties in handling the inevitable microbial contamination from the surface (Baross et al., 2004; Sheik et al., 2018; Ramírez et al., 2019).

Over the past decades, many microorganisms have been discovered within Earth’s igneous oceanic crust despite extreme conditions, such as oligotrophy, temperature gradients, active circulation, and limited available space within the rock, which were once thought unsuitable to supporting life (Cowen et al., 2003; Nakagawa et al., 2006; Orcutt et al., 2011b; Jungbluth et al., 2013, 2014; Lever et al., 2013; Baquiran et al., 2016). Studies of ridge-flank systems have demonstrated that crustal aquifers harbor aerobic mesophiles and anaerobic thermophiles, involved in hydrogen, nitrogen, carbon, and sulfur cycling (Cowen et al., 2003; Nakagawa et al., 2006; Smith et al., 2011; Lever et al., 2013; Orcutt et al., 2013; Robador et al., 2015; Jungbluth et al., 2016). The comparison of biomes in very young (<3Ma) and old (80Ma) oceanic crust indicates that microbial diversity increases with the age of the basalt and the community compositions converge toward similar profiles over time (Lee et al., 2015). The colonization of rocks and the succession of microbial communities mainly depend on the temperature and reduction-oxidation conditions (Baquiran et al., 2016; Ramírez et al., 2019). While microbial communities hosted by young basalt (<10Ma) have been detected (Konhauser et al., 2002; Templeton et al., 2005), our knowledge of the pioneering communities inhabiting newly erupted oceanic basalt (<100years) is extremely limited.

**Figure 1:**
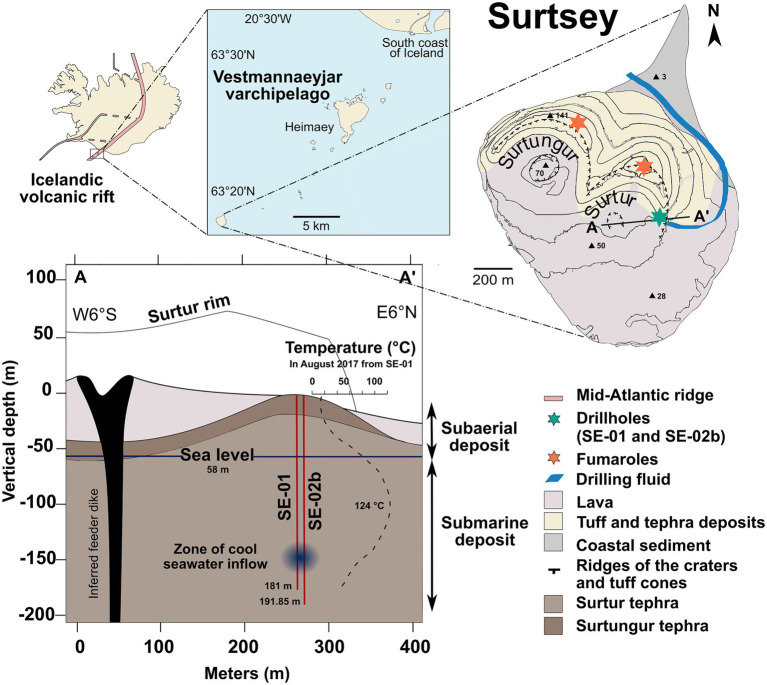
Location of the study area of the south coast of Iceland. Map of the Surtsey volcanic island within the Westman Islands (Vestmannaeyjar) archipelago and location of sampling sites: 1979 borehole SE-01 and 2017 borehole SE-02b (green star), drilling fluid (blue line), and fumaroles (orange stars). Schematic cross section of Surtur crater showing the geological setting of the subsurface deposits and the location of the time-lapse cored boreholes (see Jackson et al., 2019b) and a zone of seawater inflow.

A recent drilling operation has addressed this gap in knowledge through the study of the volcanic island, Surtsey, located on the southern offshore extension of the Icelandic volcanic rift zone (Figure 1). The island is the visible part of a volcano formed by underwater and basaltic eruptions from the seafloor between 1963 and 1967 (Þórarinsson, 1965; Jakobsson and Moore, 1982; Jakobsson et al., 2009). Since the initiation of eruptive activity, the entire area has been accessible only to scientific investigations. Surtsey is the site of long-term longitudinal studies, which have provided a unique record of pioneering species of plants and animals colonizing the surface of the basaltic deposits (Baldursson and Ingadóttir, 2007; Magnússon et al., 2014). In 1979, a 181m core (SE-01) drilled through the eastern sector of the Surtur vent (Figure 1), probed the hydrothermal system in the subaerial and the submarine deposits – above and below coastal sea level, respectively (Jakobsson and Moore, 1982, 1986; Jackson et al., 2019a). In 2017, three new cored boreholes (SE-02a, SE-02b and SE-03) were acquired through the International Continental Scientific Drilling Program (ICDP) 5,059 expedition, SUSTAIN drilling operation (Jackson et al., 2015, 2019b; Weisenberger et al., 2019). Annual monitoring of temperatures in the 1979 borehole, SE-01, indicates that the hydrothermal system has cooled down over the years. The maximal temperature in 1980 was 141.3°C at 100m depth, and it has decreased gradually to 124.6°C in 2017 (Figure 1; Jakobsson and Moore, 1986; Ólafsson and Jakobsson, 2009; Marteinsson et al., 2015; Jackson et al., 2019b). The current temperatures exceed a presumed upper limit for functional microbial life (122°C; Prieur and Marteinsson, 1998; Kashefi and Lovley, 2003; Takai et al., 2008). The highly porous subaerial and submarine tephra deposits were largely transformed to palagonitized lapilli tuff by 1979, described in studies of the SE-01 drill core (Jakobsson and Moore, 1982; Jakobsson and Moore, 1986). Progressive alteration was recorded in samples from a parallel, time-lapse drill core acquired in 2017, SE-02b (Figure 1; Prause et al., 2020). A recent study of geothermal water chemistries in the 1979 and 2017 Surtsey boreholes indicates depletion in boron, magnesium, iron, carbon dioxide, and sulfate concentrations, suggesting that the fluid compositions in the subsurface deposits are controlled by seawater-basalt interactions and temperature (Kleine et al., 2020). This further suggests that fluid-rock interactions in the submarine Surtsey basaltic deposits behave similarly to those interactions in basaltic oceanic crust, where the chemical composition of rocks and fluids changes and introduces organic matter and oxygen into the system (Furnes and Staudigel, 1999; Edwards et al., 2005; Orcutt et al., 2011a). The Surtsey volcano geothermal system represents an exceptional natural laboratory for studying fluid-rock-microbe interactions at temperatures approaching the presumed thermal limit for functional life on Earth. Its boreholes can be viewed as windows opened from the land surface that allow the study of subsurface processes at high temperature associated with the basaltic oceanic crust.

Several studies have described the volcanic structure of Surtsey and the geochemical, chemical, and mineralogical changes in the altered basaltic deposits (Jakobsson and Moore, 1982; Jakobsson and Moore, 1986; Schipper et al., 2016; McPhie et al., 2020; Moore and Jackson, 2020; Prause et al., 2020). Little is known, however, about the microorganisms inhabiting the subsurface and their metabolic potential. A pioneering study by Marteinsson et al. (2015) detected archaea of the *Archaeoglobus* genus at 145m depth (80°C) and one taxon from the Methanobacteriales order in the SE-01 borehole at depths to 170m (55°C; Marteinsson et al., 2015). Furthermore, recent studies of 1979 and 2017 drill core samples show abundant microtubules in basaltic glass that resemble endolithic microborings (Jackson et al., 2019a; Jackson, 2020); these are thought to indicate microbial microboring into glass (Fisk et al., 2003; Staudigel et al., 2008; Walton, 2008; Mcloughlin et al., 2010). These investigations suggest that Surtsey’s basalt and fluids could reveal new information about the microbial communities associated with the young hydrothermal-seawater system and the newly formed oceanic basaltic crust.

This work focuses on the following questions: (i) how can we distinguish the true residents of the subsurface from the marine microbial taxa derived from the drilling fluid? (ii) which microorganisms inhabit Surtsey’s basalt and fluids? (iii) does life persist in the subsurface zones crossing the presumed thermal limit for life? and (iv) can we detect actual microbial structures or biotic signatures in the basaltic cores only 50years after eruptions terminated? We used high-throughput sequencing (16S rRNA gene amplicon sequence analysis) and scanning electron microscopy (SEM) to address these questions and enable an in-depth characterization of the basalt-hosted microbial communities within the volcanic system. Microbial communities from drill core samples collected at successive depths from the subaerial and submarine basaltic tephra deposits were compared to the communities in borehole fluid samples associated with the active hydrothermal system and in fumarole samples from the surface of the island and seawater samples collected several kilometers offshore. In this study, it was of crucial importance to collect drilling fluids, sequence control samples representing potential sources of contamination, and implement computational methods to distinguish the endemic microbial taxa of the subsurface rocks from the microorganisms found in the drilling fluid and other potential contaminants. Here, we report our strategy to identify and minimize contaminants in the data sets; we describe the archaeal and bacterial taxa that are candidate residents of the oceanic crust; we discuss the microbial colonization and dissemination from the surrounding ecosystems, and we explore microbial metabolic potentials. The results provide unique data on subsurface microbial life in one of the newest and most pristine oceanic basalt environments on Earth.

## Materials and Methods

### Site Description

The volcanic island of Surtsey is located in the Vestmannaeyjar archipelago, approximately 35km from the south coast of Iceland (63°18'10.8'N; 20°36'16.9'W; Figure 1) within the southern offshore extension of Iceland’s Eastern Volcanic Zone. The ocean depth was 130m below sea level prior to eruption. After 3.5years of submarine, explosive, and effusive lava-producing eruptions of basalts from the seafloor in 1963–1967, Surtsey had a subaerial area of 2.6km^2^, and the highest point on the island was 174m above sea level. The island has now eroded to less than 1.2km^2^ and a height of 150m above sea level (Baldursson and Ingadóttir, 2007; Óskarsson et al., 2020).

The cores extracted from the SE-01 borehole in 1979 and the SE-02b borehole in 2017 indicate that the subsurface of Surtsey consists mainly of lithified tephra, mainly lapilli tuff, and minor amounts of weakly consolidated tephra and alkali olivine basalt intrusions (Jakobsson and Moore, 1982; McPhie et al., 2020; Figure 1). The principal authigenic minerals in the lapilli tuff are smectitic clay mineral (nontronite and clinochlore), analcite, phillipsite, Al-tobermorite, and anhydrite (Jakobsson and Moore, 1986; Jackson et al., 2019a; Prause et al., 2020). Alteration through palagonitization and production of authigenic cementitious minerals has progressed during the past 38years, from 1979 to 2017 (Jackson, 2020; Prause et al., 2020). *In situ* subsurface fluid temperatures have been measured annually since 1980 in SE-01, usually at 1-m intervals from the surface to the bottom of the borehole (e.g., Jakobsson and Moore, 1986; Marteinsson et al., 2015). Geochemical analyses and pH measurements of borehole fluids and pore water extracted from the 2017 drill cores are described by Kleine et al. (2020).

### Drilling Operation and Drilling Fluid

One of the principal objectives of the 2017 Surtsey Underwater volcanic System for Thermophiles, Alteration processes and Innovative concretes (SUSTAIN) drilling project at Surtsey volcano, sponsored in part by the ICDP, was to investigate microbial diversity within the basaltic tephra. All possible precautions were made to avoid microbial contamination (Jackson et al., 2019b). Water from the sea was pumped to the drill site and used as drilling fluid since no fresh water is available on the island (Figure 1). The conventional methods used to track contamination during drilling operations, such as the addition of tracer compounds in the circulating fluid, could not be applied during drilling at Surtsey due to the strict environmental protection of the island (see discussion). To avoid contamination, drilling fluid was sterilized using two filtration units with a pore size of 30μm (Pentek Big Blue, R30-BB 30 Micron cartridge filter, Lenntech, The Netherlands) and two ultraviolet sterilization devices (AQUA4ALT from WEDECO, Aquaculture systems, Xylem Water Solutions Herford GmbH, Germany) with a maximum flow rate of 1.58ls^−1^. The decontaminated drilling fluid was stored in 1000L containers before pumping into the borehole. To track potential problems with the sterilization system, 1l of drilling fluid was collected regularly at different drilling depths to assess potential microbial contaminants (Figure 1).

### Sample Collection

Four types of samples were collected for molecular investigation and comparison of microbial diversity among these sample types: drill cores from the 2017 SE-02b borehole, borehole fluids from the 1979 SE-01 borehole, steam from surface cracks of fumaroles, and seawater samples collected few kilometers from the northwest coast of Surtsey (Figure 1). A description of the samples is shown in Table 1.

**Table 1:** Sample description table: locations, sampling date, depth, temperature, and DNA concentration.

Sample ID	Sample types and categories	Sample site	Sampling date	Collection depth (m b.s.)	Collection Temp (°C)	Sample amount	DNA concentration NanoDrop (ng/μl)	260/280	DNA concentration QuBit dsDNA (ng/μl)
Sur161	Borehole fluid	SE-01	6/9/2016	164	56.56	500ml	16.14	1.41	0.16
Sur162	Borehole fluid	SE-01	6/9/2016	164	56.56	300ml	15.48	1.35	0.30
Sur163	Borehole fluid	SE-01	6/9/2016	166	54.24	500ml	26.65	1.43	Too low
Sur164	Borehole fluid	SE-01	6/9/2016	162	58.88	400ml	8.33	1.29	Too low
Sur165	Borehole fluid	SE-01	6/9/2016	70	109.17	250ml	56.12	1.56	0.09
Sur166	Borehole fluid	SE-01	6/9/2016	100	124.62	500ml	34.07	1.58	0.15
Sur167	Borehole fluid	SE-01	6/9/2016	100	124.62	250ml	38.24	1.50	0.81
Sur168	Borehole fluid	SE-01	6/9/2016	120	115.78	550ml	97.37	1.61	0.56
Sur169	Borehole fluid	SE-01	6/9/2016	160	61.27	500ml	17.59	1.05	0.13
Sur1610	Borehole fluid	SE-01	6/9/2016	160	61.27	150ml	12.31	1.21	0.11
Sur16Mix	Borehole fluid	SE-01	6/9/2016	mix	n.a.	1,400ml	52.23	1.14	0.15
Sur171	Borehole fluid	SE-01	**8/3/2017**	58	87.75	5,000ml	48.80	1.49	n.a.
Sur172	Borehole fluid	SE-01	8/3/2017	120	115.78	1,820ml	38.25	1.46	n.a.
Sur173	Borehole fluid	SE-01	8/3/2017	mix	n.a.	980ml	35.38	1.49	n.a.
Sur174	Borehole fluid	SE-01	8/3/2017	mix	n.a.	805ml	23.07	1.53	n.a.
Sur175	Borehole fluid	SE-01	8/3/2017	150	74.22	5,000ml	52.80	1.45	n.a.
Sur176	Borehole fluid	SE-01	8/8/2017	160	61.27	5,000ml	35.95	1.49	n.a.
Sur177	Borehole fluid	SE-01	8/8/2017	mix	n.a.	850ml	16.59	1.58	n.a.
C4	Drill core (DC_1)	SE-02b	8/22/2017	23*	20.00	15g	181.61	1.42	0.17
C9	Drill core (DC_1)	SE-02b	8/22/2017	35*	36.00	15g	196.47	1.44	0.14
C13	Drill core (DC_2)	SE-02b	8/22/2017	44*	59.00	15g	202.15	1.33	0.16
C17	Drill core (DC_2)	SE-02b	8/23/2017	55*	82.50	15g	137.89	1.43	0.11
C22	Drill core (DC_2)	SE-02b	8/23/2017	65	101.50	15g	115.60	1.46	0.41
C27	Drill core (DC_3)	SE-02b	8/23/2017	78	114.00	15g	204.83	1.42	0.11
C33	Drill core (DC_3)	SE-02b	8/24/2017	93	123.00	15g	202.74	1.38	0.09
C36	Drill core (DC_3)	SE-02b	8/24/2017	102	124.00	15g	202.32	1.41	0.16
C39	Drill core (DC_3)	SE-02b	8/24/2017	111	121.50	15g	197.72	1.35	0.15
C42	Drill core (DC_3)	SE-02b	8/24/2017	120	116.00	15g	57.15	1.43	0.17
C45	Drill core (DC_3)	SE-02b	8/24/2017	130	107.00	15g	171.05	1.40	0.15
C49	Drill core (DC_3)	SE-02b	8/25/2017	139	97.00	15g	168.91	1.41	0.11
C52	Drill core (DC_4)	SE-02b	8/25/2017	148	84.00	15g	180.71	1.44	0.10
C55	Drill core (DC_4)	SE-02b	8/25/2017	157	64.00	15g	97.89	1.44	0.06
C59	Drill core (DC_4)	SE-02b	8/25/2017	166	55.00	15g	148.14	1.43	0.10
C62	Drill core (DC_4)	SE-02b	8/25/2017	175	44.50	15g	178.08	1.42	0.13
C65	Drill core (DC_4)	SE-02b	8/25/2017	181	37.00	15g	203.00	1.40	0.13
Fum_1	Fumarole	63°18'19.9'N 20°36'24.7'W	8/4/2017	0	82.30	350ml	19.05	1.88	Too low
Fum_2	Fumarole	63°18'15.4'N 20°36'07.7'W	8/4/2017	0	85.60	5,000ml	8.39	2.33	Too low
SW_10	Seawater	63°28'58.8'N; 20°54'7.2'W	8/18/2017	10 (m b.s.l.)	12	1,000ml	330.37	2.00	n.a.
SW_20	Seawater	63°28'58.8'N; 20°54'7.2'W	8/18/2017	20 (m b.s.l.)	11.97	1,000ml	607.05	1.58	n.a.
SW_30	Seawater	63°28'58.8'N; 20°54'7.2'W	8/18/2017	30 (m b.s.l.)	10.81	1,000ml	449.85	1.85	n.a.
SW_50	Seawater	63°28'58.8'N; 20°54'7.2'W	8/18/2017	50 (m b.s.l.)	9.9	1,000ml	175.98	1.81	n.a.
1B0ZC	Drilling fluid	63°18'30.7'N 20°36'21.0'W	8/9/2017	1 (m b.s.l.)	10.00	1,000ml	220.69	1.98	n.a.
1B3ZC	Drilling fluid	63°18'30.7'N 20°36'21.0'W	8/10/2017	1 (m b.s.l.)	10.00	1,000ml	92.74	1.91	n.a.
1B25ZC	Drilling fluid	63°18'30.7'N 20°36'21.0'W	8/12/2017	1 (m b.s.l.)	10.00	1,000ml	103.01	2.03	n.a.
149ZC	Drilling fluid	63°18'30.7'N 20°36'21.0'W	8/16/2017	1 (m b.s.l.)	10.00	1,000ml	129.22	1.90	n.a.
1C17ZC	Drilling fluid	63°18'30.7'N 20°36'21.0'W	8/23/2017	1 (m b.s.l.)	10.00	1,000ml	118.26	1.97	n.a.
1C39ZC	Drilling fluid	63°18'30.7'N 20°36'21.0'W	8/24/2017	1 (m b.s.l.)	10.00	1,000ml	96.39	1.91	n.a.
1C51ZC	Drilling fluid	63°18'30.7'N 20°36'21.0'W	8/25/2017	1 (m b.s.l.)	10.00	1,000ml	252.97	2.05	n.a.
1C59ZC	Drilling fluid	63°18'30.7'N 20°36'21.0'W	8/24/2017	1 (m b.s.l.)	10.00	1,000ml	152.45	2.04	n.a.
Cw	Control	n.a.	n.a.	n.a.	n.a.	n.a.	27.11	1.74	Too low
Cr	Control	n.a.	n.a.	n.a.	n.a.	15g	25.05	1.69	Too low

* Drill core samples from the subaerial tuff cone, located above the sea level. Too low: for detection with Qubit fluorometer and high-sensitivity dsDNA reagents, <0.5 ng/ml. n.a.: not available. m b.s.l.: meter below sea level. m b.s.: meter below surface.

Seventeen drill core samples were collected for microbial analyses from the vertical SE-02b cored borehole that extends to 192m below surface and terminates in poorly consolidated tephra a few meters above the presumed depth of the pre-eruption seafloor (Jackson et al., 2019b). At the drill site, drill core samples were collected from every third 3-m core run for molecular analyses by cutting a 10-cm section at 70cm from the top of the core run. Immediately after sampling, each section was kept in the plastic core liner, taped at both ends, wrapped in a plastic bag, kept in liquid nitrogen on site, and at −80°C for long-term laboratory storage. Fluid samples from SE-01 borehole were collected in 2016 and in 2017, before drilling operations started, using a custom sampler made of stainless steel, as described in Marteinsson et al. (2015). The sampler was rinsed with 70% ethanol before each sampling. Eighteen fluid samples were collected from the SE-01 borehole: 18 in 2016 and seven in 2017 (Table 1). Steam from fumaroles, located on the summit of Surtur, the eastern tephra cone (~150m above sea level; 63°18'15.4'N 20°36'07.7'W) and Surtlungur, the western tephra cone (63°18'19.9'N 20°36'24.7'W) were collected in 2017 by introducing a sterile rubber hose into the outlet of the fumarole, with the other end connected to a sterile plastic container. This generated 5,350ml of condensed water from the fumaroles over 12h of continuous sampling. Four liters of seawater samples were collected 25km offshore during the drilling operation (63°28'58.8'N; 20°54'7.2'W). All water samples (drilling fluid, borehole fluid, fumarole, and seawater samples) were immediately filtrated through 0.22-μm Sterivex^™^ filters (Merck Millipore). The filters were stored in liquid nitrogen on site and at −80°C for long-term storage in the laboratory.

### DNA Extraction

#### DNA Extraction From Rock Samples

A modified PowerMax^®^ Soil DNA Isolation Kit protocol (MO BIO Laboratories, Inc.) was applied to extract the DNA from the drill core samples. Small fragments of tuff from the interior of the 17 frozen core samples from SE-02b were broken aseptically. After 2min pre-cooling on ice, 15g were cryo-ground at an impact rate of 8cycles per second for 1min using a 6,700 Freezer/Mill cryogenic grinder (SPEX). Phosphate-ethanol solution (1M phosphate buffer, 15% ethanol, pH 8.0; Direito et al., 2012) and proteinase K (20mg/ml) were added to the lysis solution provided by the kit. Samples were vortexed twice for 30s with a 1min cooling step in between instead of the bead-beating step, followed by incubations at 55°C for 1h and at 80°C for 40min. Subsequent DNA isolation was carried out according to the standard PowerMax^®^ Soil DNA isolation protocol. DNA precipitation was done overnight with isopropanol (0.7 volume) and glycogen (20mg/ml). After a washing step with 70% ethanol, DNA was resuspended in Tris buffer (10mM, pH 8) and quantified using both NanoDrop^®^ ND-1000 UV-Vis Spectrophotometer (Thermo Fisher Scientific) and a Qubit fluorometer and high-sensitivity dsDNA reagents (Invitrogen^™^) before being stored at −20°C. Attempts to estimate the microbial biomass within the drill core samples using fluorescence microscopy were unsuccessful because of the non-biological background signals that occurred during recognition of cells for counting. Two controls were carried out to test for contamination during DNA extraction of the core samples. One used sterile water (instead of the basalt powder) to test for contaminants derived from the kit and reagents. The other used 15g of basalt powder from a drill core that had been treated with 70% ethanol and heated at 180°C for 24h. Both controls resulted in amplifications with primers targeting bacterial and archaeal 16S rRNA genes, which were then sequenced.

#### DNA Extraction From Fluid Samples

DNA was extracted from Sterivex^™^ filters containing biomass from borehole water, condensed fumarole water and drilling fluid samples following a modified protocol by Neufeld et al. (2007). Sucrose EDTA Tris buffer (SET buffer: 40mM ethylenediaminetetraacetic acid (EDTA), 50mM Tris-HCl pH 9, and 0.75M sucrose) and 20mg/ml lysozyme solution were added to the Sterivex^™^ filters. Filters were incubated at 37°C for 30min. After the addition of 10% (w/v) sodium dodecyl sulfate (SDS) and proteinase K (20mg/ml), the filters were incubated with rotation at 55°C for 2h. Lysates were collected into syringes, while the filters were rinsed twice with SET buffer and the rinsed buffer was combined to the lysate. One volume of phenol:chloroform:isoamyl alcohol (PCI: 25:24:1, pH 8) was added, and the aqueous phase was transferred to a new tube after a 15-min centrifugation at 10,000×*g* at 4°C. The phenol was removed from the aqueous phase by adding 1 volume of chloroform. The cleaned aqueous phase was transferred to a new tube after a 5-min centrifugation at 10,000×*g* at 4°C, and 0.7 volume of cold isopropanol was added. After inverting the tube several times, the sample was incubated for 15min at room temperature and then overnight at −20°C. After a 20-min centrifugation at 16,000×*g* at 4°C, the DNA pellet was washed twice with 75% (v/v) ethanol, dried for 5min using a SpeedVac^™^ and 15min at room temperature, and finally resuspended in sterile Tris-HCl buffer (10mM, pH 8). DNA was quantified using a NanoDrop^®^ ND-1000 UV-Vis Spectrophotometer (Thermo Fisher Scientific) and Qubit fluorometer (Invitrogen, Quant-iT^™^ dsDNA HS) and stored at −20°C. One negative extraction control was carried out for each round of extractions by rinsing a sterile Sterivex^™^ filter with sterile laboratory-grade water.

### Partial 16S rRNA Gene Amplification and Tag Sequencing

Illumina MiSeq paired-end (2×300 base pair) tag sequencing was carried out using the Earth Microbiome Project universal primers 515f (5'-GTG CCA GCM GCC GCG GTA A-3') and 806r (5'-GGA CTA CHV GGG TWT CTA AT-3'), which amplify the V4 region of the bacterial and archaeal 16S rRNA genes (Caporaso et al., 2012). Since this primer pair does not show a high coverage for Archaea (Parada et al., 2016), specific archaeal primer sets were used with a nested PCR approach. The first-round PCR was performed to amplify the V3-V5 region of archaeal 16S rRNA gene with the primer set Parch340F (5'- CCC TAY GGG GYG CAS CAG -3'; Øvreås et al., 1997) and Arch958VR (5'- YCC GGC GTT GAV TCC AAT T -3'; Klindworth et al., 2013). Then, a second round was performed on the first PCR product to amplify the V3 region of archaeal 16S rRNA gene with the primer set Arch349F (5'- GYGCASCAGKCGMGAAW -3'; Takai and Horikoshi, 2000) and Parch519R (5'- TTACCGCGGCKGCTG -3'; Øvreås et al., 1997).

All PCR reactions were carried out in 25μl reactions with 20μl of Q5^®^ High-Fidelity PCR Master Mix (New England Biolabs, MA, United States) following the manufacturer’s amplification protocol and 5μl of DNA at 10ng/μl (NanoDrop quantification). The second round of the nested PCR used 5μl of amplified DNA from the first round. Bovine serum albumin was added to the master mix at a final concentration of 0.5ng/μl for rock samples that could not be amplified. Thermal cycling consisted of an initial denaturation step at 98°C for 30s, followed by 30cycles of denaturation at 98°C for 10s, annealing at 52°C (using the bacterial primer set) or at 55°C (using the archaeal primer sets) for 30s, and elongation at 72°C for 60s. Final elongation was set at 72°C for 2min. Amplification products were visualized on 1% (w/v) agarose gels.

Sequencing libraries were generated using the “16S Metagenomic Sequencing Library Preparation guide” from Illumina and barcodes from the Illumina Nextera^®^XT DNA Sample Preparation Kit (8cycles for index PCR). The libraries were assessed on a Qubit Fluorometer (Invitrogen, Quant-iT^™^ dsDNA HS) and a Bioanalyzer system (Agilent Technologies). After normalization and quantification, the final pooled library was loaded on a MiSeq Desktop sequencer (Illumina) and sequenced with V3 chemistry and 2×300cycles across two sequencing runs. Raw sequences have been deposited in the European Nucleotide Archive (ENA) at EMBL-EBI under accession number ERP126178.

### 16S rRNA Gene Amplicon Sequence Analysis

Bioinformatic analysis was conducted in RStudio running R version 4.0.2 (R Core Team, 2019; Rstudio, 2020). Sequence variants were inferred using the R Package DADA2 (Callahan et al., 2016) version 1.4, available at https://benjjneb.github.io/dada2/tutorial.html. The following trimming parameters were used: trimLeft=10, maxN=0, maxEE=c(2,5), truncQ=2, and truncLen=c(230,215) for the universal primer set and truncLen=c(115,110) for the archaeal primer sets. After quality screening and trimming, forward and reverse reads were merged to remove chimeric variants and singletons and to identify amplicon sequence variants (ASV). Non-target-length sequences were removed, and only amplicons of 270–275bp length were kept with the universal primer set and amplicons of 130–165bp for the archaeal primer sets. Data sets from three MiSeq sequencing runs (universal primer set) were processed separately using the same pipeline and same parameters and merged into a unique ASV table using the function “mergeSequenceTables.” The taxonomy was assigned using the function “assignTaxonomy” (minimum bootstrap confidence at 50) and the SILVA SSU database release 138 (Quast et al., 2013).

In total, all 51 samples were successfully sequenced using the universal primer set, including extraction controls and drilling fluid samples. The R package Decontam (version 1.10.0; Davis et al., 2018^1^) was used to identify contaminant ASVs in the universal primer data set. We identified 160 contaminant ASVs (Supplementary Table S1) using the Decontam prevalence method (threshold value of 0.5), based on the prevalence comparison of each sequence in true samples and negative controls (Supplementary Figure S1). In addition, sequences that were identified as chloroplast at the order level, mitochondria at the family level, Eukaryote at the kingdom level, and those that could not be identified at the kingdom level were subtracted from the data set prior to analysis, as well as putative contaminants identified by taxonomic affiliation at the genus level by a study on common contaminants from the Census of Deep Life data set (Sheik et al., 2018). Supplementary Table S2 gives a list of 95 potentially contaminant genera removed from the analyses. A total of 588,510 sequences were removed from the samples by the abovementioned procedures (Details are available in Supplementary Table S3).

From the archaeal nested PCR, 17 samples were analyzed. Sequences identified at the kingdom level as Eukaryote, Bacteria, or not assigned were removed from libraries prior to analysis, as well as sequences detected in the DNA extraction blanks (Supplementary Table S4, 26 ASVs). Using the archaeal primer sets, the number of sequences removed from the samples was 74,315 (Details available in Supplementary Table S3).

### Microbial Community Analysis

Microbial community analysis (α and β diversity, community composition, and statistical analysis) was conducted in R (R Core Team, 2019) with the Phyloseq (McMurdie and Holmes, 2013) and Vegan (Oksanen et al., 2007) packages. ANOVA and Tukey’s HSD (Honestly Significant Difference) test were conducted to evaluate the differences in α diversity values. For β diversity assessment, the data were normalized using “rarefy_even_depth” function prior to performing a non-metric multidimensional scaling (NMDS) ordination on Bray-Curtis dissimilarities. The significance of sample type variable was assess using permutational multivariate analysis of variance (PERMANOVA) using distance matrices and multilevel pairwise comparison (Martinez Arbizu, 2020). The command envfit was used to investigate the correlation between the community structure and environmental variables (depth and temperature). Finally, DESeq2 was used to identify ASVs significantly different among sample types (F, fumarole; BF, borehole fluid; DC, drill core; SW, seawater samples) and categories of drill cores (DC_1, DC_2, DC_3 and DC_4; Love et al., 2014). Details of the data analyses can be found in the Supplementary Material. Predictive functional analyses of the prokaryotic communities were performed using Phylogenetic Investigation of Communities by Reconstruction of Unobserved States 2 (PICRUSt2) on both data sets, obtained with the universal primer set and the archaeal primer sets, separately (Douglas et al., 2020). From the universal data set, a total of 2,333 PICRUSt2 predicted KEGG orthologs (enzymes) were collapsed into 425 MetaCyc pathways, while 828 predicted KEGG orthologs and 124 MetaCyc pathways were obtained from the archaeal data set. Only few MetaCyc pathways were selected to represent sulfur, nitrogen, methane, and carbon metabolism (Supplementary Figures S8, S9).

### Microscopy

For SEM images, drill core samples were crushed in a sterile mortar to obtain rock grains <0.5mm diameter, which were dehydrated by four 10-min wash steps in increasing concentrations of ethanol (30, 50, 80, and 100%). After drying, samples were placed on carbon conductive tabs (PELCO Tabs^™^, 9mm) and gold-coated. SEM used a Zeiss Auriga 40 Focused Ion Beam Field Emission Scanning Electron Microscope coupled with an energy dispersive X-ray spectroscopy analyzer (EDX) at the Institute de Physique du Globe de Paris (University Sorbonne Paris Cité, Paris, France) using two types of secondary electron detectors: In-Lens and SESI, and a backscattered electron detector: EsB. The acceleration voltage (EHT) ranged from 5 to 15kV.

## Results

### DNA Yields From Rocks

Based on Qubit quantification, DNA concentration extracted from 15g of each drill core sample yields from 0.2 to 1.36ng.g^−1^ of rocks with an average of 0.48ng.g^−1^. However, no significant correlations in DNA yield were apparent with sampling depth or *in situ* temperature (Supplementary Figure S2).

### Special Considerations for the Drilling Operation and the Drilling Fluid

The island of Surtsey is subjected to strict environmental protection and is a UNESCO World Heritage site (Baldursson and Ingadóttir, 2007). For this reason (and other reasons mentioned later in the study), the use of tracer compounds to track contamination could not be considered during the design of the 2017 SUSTAIN drilling operation. Instead, a different approach was used to assess contamination. Filtered and UV-sterilized water pumped from the sea was used as drilling fluid (see above). Aliquots of those fluids were then collected to track microbial DNA potentially contaminating the core. DNA extracted from 1l of each drilling fluid sample yields ~96 to 252ng/μL of DNA (Table 1).

The 16s rRNA gene sequencing of the drilling fluid samples thus showed, unfortunately, that the sterilization system failed (Supplementary Table S3, Supplementary Figure S3). Consequently, marine microbial taxa from the drilling fluid were introduced into the subsurface. The failure can possibly be explained by the clogging of filters and an overly high flow rate for the UV system.

**Figure 2:**
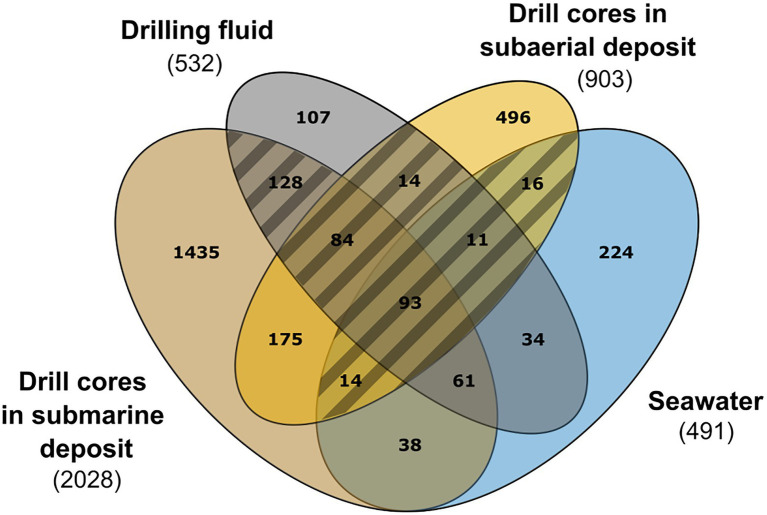
Contamination assessment by an overlap approach between drill cores from the subaerial and submarine deposits, drilling fluid, and seawater samples. Five drilling fluid samples (in grey): 1B49ZC, 1C17ZC, 1C39ZC, 1C51ZC, 1C59ZC, 4 sea samples (in blue): SW_10, SW_20, SW_30, SW_50, 4 samples from the subaerial deposit (in yellow): C4, C9, C13 and C17, and 13 samples from the submarine deposit (in light brown): C22, C27, C33, C36, C39, C42, C45, C49, C52, C55, C59, C62, C65. Hatched area: 360 ASVs considered as contaminants.

As the contamination of the samples was evidently pervasive, the data were evaluated with caution. A NMDS ordination plot conducted using Bray-Curtis dissimilarity metrics (stress value=0.124) revealed that some of the drill core samples (e.g., C45, C52, C65) showed a microbial community composition similar to the drilling fluid and seawater samples (Supplementary Figure S4). Whereas differences in microbial community structure between drill core and drilling fluid and between drill core and seawater samples proved to be significant by a Tukey HSD test (p adjusted values of 0.01 and 0.0008, respectively), a strategy was adopted to distinguish endemic microbial taxa of Surtsey subsurface rocks from the marine residents introduced to the subsurface by the drilling fluid. The distributions of individual ASVs were evaluated by a simple overlap approach using a Venn diagram to compare ASVs shared among the seawater samples, drilling fluid, drill core from the subaerial deposits, and from the submarine deposits (Figure 2).

In the subaerial tephra deposits, located above coastal sea level, meteoric water was presented before drilling. In the zone of tidal flux at ~58m b.s., temperatures up to 100°C could operate as a natural autoclave or biological barrier, preventing transfer of live microorganisms between the subaerial and submarine basaltic deposits (Ólafsson and Jakobsson, 2009; Marteinsson et al., 2015). Hence considering that no marine taxa should be detected in the subaerial deposit, all ASVs shared between the subaerial samples (C4, C9, C13, and C17; 23–55m b.s.; 20–82.5°C), the drilling fluid, and the seawater samples (Figure 2: 93+84+14+11+16+14 ASVs, Supplementary Table S6) were considered as potential marine contaminants; these sequences were removed from the data set. In a second iteration, ASVs shared only between the drill core samples located in the submarine deposits and the drilling fluid (Figure 2, Supplementary Table S6, 128 ASVs) were also considered as marine contaminants and were removed from the data set. Natural infiltration of cool seawater occurs in the subsurface of Surtsey at 144–155m b.s (Jakobsson and Moore, 1986; Jackson et al., 2019b; Kleine et al., 2020). This suggests that marine taxa detected in the submarine deposit could derive from the inflow of seawater that infiltrate the subsurface. Therefore, ASVs shared between the submarine drill core, seawater samples, and the drilling fluid were retained (Figure 2, 61 ASVs). The total number of sequences removed from the drill core samples by the abovementioned procedures was 138,913 (Supplementary Table S3), which represents on average 47% of the reads per drill core samples. The subsequent analyses were performed on the decontaminated data set, excluding the drilling fluid samples.

### Microbial Community Structure Among the Sample Types

Sequencing provided enough reads to capture the total richness of the samples as all libraries reached saturation in rarefaction curves (Supplementary Figure S5). The 41 samples were catalogued by sample type: 18 samples from borehole fluids, 17 samples from drill cores, 2 samples from fumaroles, and 4 seawater samples (Table 1, Supplementary Table S3). Amplification of the partial 16S rRNA gene using the universal primer set was successful for all samples, while only 17 samples could be amplified using the archaeal primer sets.

We obtained a total of 455,545 and 848,053 high-quality sequences using universal and archaeal 16S rRNA primer sets, respectively (Supplementary Table S3). A total of 4,222 ASVs ranging from 41 (Sur168) to 317 (SW50) ASVs per sample were obtained using the universal primer set, whereas a total of 157 ASVs ranging between 3 (Sur4a) and 41 (Sur1a) ASVs per sample were obtained using the archaeal primer sets.

#### α Diversity

**Figure 3:**
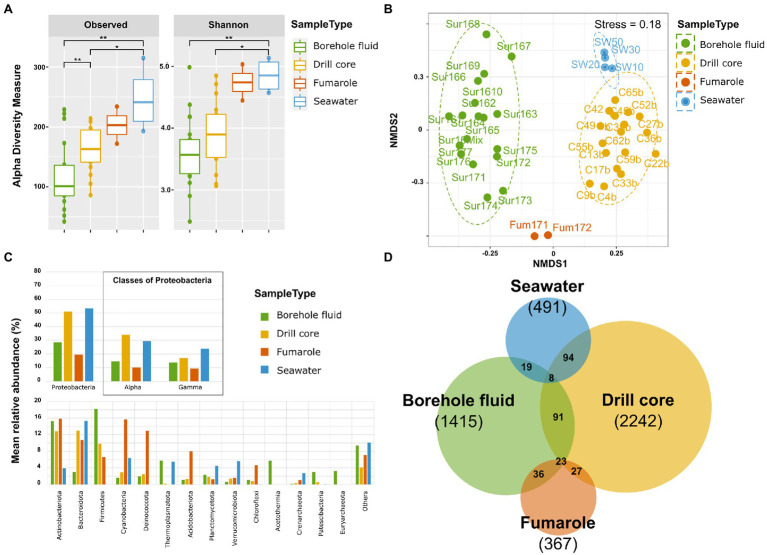
Overview of the microbial community diversity, structure, and composition of the samples types: borehole fluid, drill core, fumarole, and seawater samples. **(A)** α diversity indices: observed diversity and Shannon’s diversity indexes. Signif. codes: 0.001 “**“0.01 “*”. **(B)** β diversity assessed with non-metric multidimensional scaling ordination using Bray-Curtis dissimilarity metrics of microbial community within sample types. The stress value equaled 0.18. **(C)** Mean relative abundance of the 14 most abundant phyla. Proteobacteria are divided into classes. **(D)** Venn diagram showing the number of shared and unique ASVs among the sample types.

Analysis of variance on species richness showed significant differences between sample types (ANOVA, *F* value=9.082, Pr(>F)=0.000123). A Tukey HSD test highlighted significant differences among them between seawater samples and borehole fluid (<0.001), seawater samples and drill core (0.0339), and between drill core and borehole fluid (0.0184). Shannon diversity also differed significantly among them (ANOVA, F value=5.998, Pr(>F)=0.00195), and Tukey’s HSD test revealed significant differences between seawater samples and borehole fluid (0.00323) and between seawater samples and drill core (0.02570; Figure 3A, Supplementary Table S7). The SE-01 borehole fluids displayed a significantly lower observed diversity than the drill cores, but Shannon diversity was not significantly different, indicating that the evenness of the species present in these sample types was comparable. Observed diversity and evenness were significantly higher in the seawater samples than in drill core and borehole fluid samples.

#### β Diversity

The resulting NMDS ordination conducted using Bray-Curtis dissimilarity metrics revealed distinct microbial communities specific to each sample types, which clustered individually (Figure 3B). The stress value equaled 0.18. This was further confirmed by a PERMANOVA analysis showing that sample types differed significantly [Pr(>F)=0.001, r2=274], and a multilevel pairwise comparison showed significant differences among drill cores and borehole fluids (adjusted *p* value=0.0184), seawater samples and borehole fluids (<0.001), and between seawater samples and drill cores (0.0338). This indicates that the samples within a given sample type show microbial communities that are more similar to one another than to samples from a different sample type. The cluster of SE-01 borehole fluid samples appears wider than the drill core cluster, indicating more differences in microbial community (Figure 3B).

#### Community Composition

At the phylum level, some taxa were common across the four sample types (Figure 3C). The phylum Proteobacteria dominated all sample types, for example, whereas its relative proportion varied greatly among them. Within Proteobacteria, Alphaproteobacteria was the most abundant class followed by Gammaproteobacteria. Fumarole samples showed a higher relative proportion of Cyanobacteria (15.3%), Deinococcota (12.6%), Acidobacteriota (7.84%), and Chloroflexi (4.52%) than borehole fluids and drill cores. Borehole fluids displayed the highest occurrence of the bacterial phyla Acetothermia (5.56%) and Patescibacteria (2.26%), as well as the archaeal phyla Thermoplasmatota (5.60%) and Euryarchaeota (3.13%), in comparison with the other sample types. The class Desulfotomaculia represented more than 5% of the borehole fluid community (data not shown). Nevertheless, due to the compositional nature of the data, lineage-specific enrichments necessitate further investigation, using for example a qPCR approach (Jian et al., 2020). The drill core samples show the highest sequence variants (2,242 ASVs), followed by the borehole fluids (1,415 ASVs), seawater samples (494 ASVs), and fumarole samples (367 ASVs). Most ASVs were not shared between the sample types (Figure 3D).

### Microbial Community Composition and Structure of the Rock Samples and Influence of Depth and Temperature

**Figure 4:**
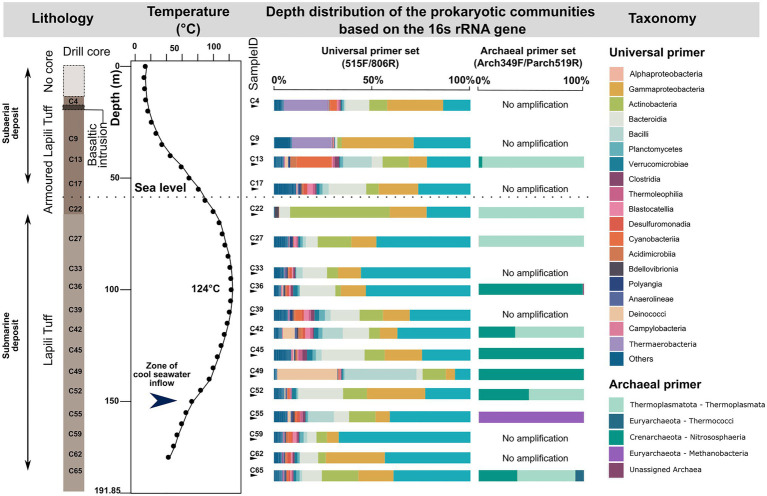
Vertical distribution of the microbial communities at the phylum or class level detected in the 17 drill core samples from the subsurface of the Surtsey volcano using universal primer set and archaeal primer sets.

The bacterial sequences from the drill core samples are classified into 35 phyla, with the ten phyla Proteobacteria, Bacteroidota, Actinobacteriota, Firmicutes, Cyanobacteria, Deinococcota, Planctomycetota, Verrucomicrobiota, Acidobacteriota, and Chloroflexi, comprising more than 95% of the sequences (Figure 4). The 29 ASVs classified as Archaea using the universal primer set fall into the phyla Thermoplasmatota, Crenarchaeota, Nanoarchaeota, and Iainarchaeota.

Archaeal sequences obtained by nested PCR from 10 of the drill cores were more diverse. The 88 unique ASVs include the phyla Crenarchaeota (mainly Nitrososphaeria), Thermoplasmatota (mainly Thermoplasmata), Euryarchaeota, Halobacterota, Hydrothermarchaeota, Nanoarchaeota, and unassigned Archaea (Figure 4). Remarkably, the sample C55 (157m b.s.; 64°C) below the submarine inflow zone is dominated by the archaeal genus *Methanobacterium,* while the sample C65 (181m b.s.; 37°C) in weakly consolidated tephra near the pre-eruption seafloor shows the highest abundance of the genus *Thermococcus* (Figure 4).

Canonical correspondence analysis (CCA) ordination (ANOVA, *p*=0.018) and envfit analyses demonstrate that both depth and *in situ* temperature are significantly correlated with microbial community structure of the drill core samples (Supplementary Figure S6). However, it is unclear which variable has the most influence on the microbial community structure, since both are linked. Observed and Shannon diversity indices showed no significant difference between the four categories of drill core samples (data not shown).

To identify the ASVs causing the dissimilarity of community structure in the CCA ordination plot, the drill core samples are grouped in four categories as follows (Supplementary Figure S6): (DC_1) drill core samples from the subaerial deposits (23–35m b.s.; 20–36°C in 2017; *n*=2, C4, C9) (DC_2) drill core samples from the zone of daily intertidal fluctuations at coastal sea level (44–65m b.s.; 59–101.5°C; *n*=3, C13, C17, C22), (DC_3) drill core samples from the submarine deposits near the hydrothermal temperature maximum (78–139m b.s.; 97–124°C in 2017; *n*=7, C27, C33, C36, C39, C42, C45, C49), and (DC_4) drill core samples from the submarine deposits, below the zone of seawater inflow (157–181m b.s.; 37–84°C in 2017; *n*=5, C52, C55, C65, C62, C59).

### Differential Abundance Analyses

**Figure 5:**
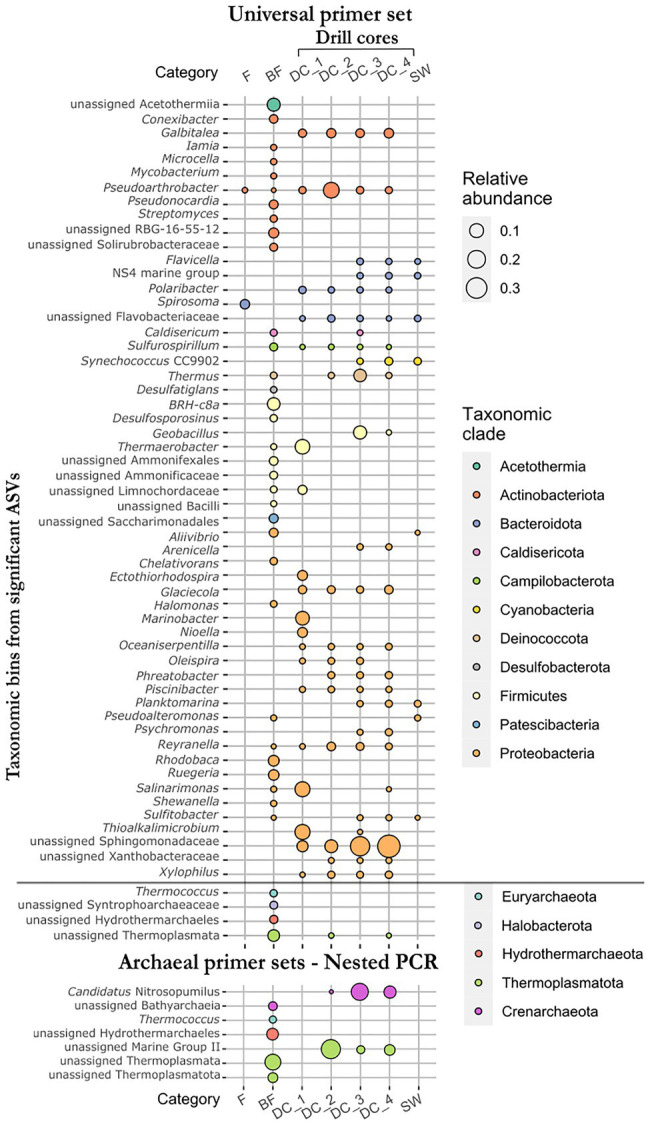
Relative abundance of taxonomic bins from all significant ASVs identified by differential abundance analyses using both universal and archaeal primer data sets. Sample types: F, fumarole (*n*=2); BF, borehole fluid (*n*=18); DC, drill core (*n*=17); SW, seawater samples (*n*=4). Categories of drill cores: DC_1, drill cores from the subaerial deposit (*n*=2); DC_2, just above and at a zone of daily intertidal fluctuations at coastal sea level (*n*=3); DC_3, in the submarine deposit at the hydrothermal temperature maximum (*n*=7); DC_4, in the submarine deposit below the zone of seawater inflow (*n*=5).

To identify differences in ASVs abundance between the four sample types (F, fumarole; BF, borehole fluid; DC, drill core; SW, seawater samples), we performed differential abundance analyses (two-by-two comparisons) using separately the universal primer and the archaeal primer data sets (Supplementary Table S5). Additionally, the same analyses were performed for the four categories of drill core samples to highlight the ASVs contributing to the dissimilarity in the CCA plot (Supplementary Figure S6). Due to the compositional features of the data, the identification of differentially abundant taxa between the different groups of samples must be assessed carefully, since it is based on a count-based method. Being aware of the limitations of this approach, a total of 95 and 26 unique ASVs were proved to be significantly represented using the universal primer data set (grouping into 59 taxonomic bins) and the archaeal primer sets (7 taxonomic bins), respectively (Figure 5), in accordance with the log fold change of the mean normalized read counts (*p*<0.01).

Compared to the seawater samples, the drill cores showed overrepresented ASVs assigned to the taxa *Caldisericum*, *Galbitalea*, *Geobacillus*, *Glaciecola*, *Oceaniserpentilla*, *Oleispira*, *Piscinibacter*, *Pseudoarthrobacter*, *Psychromonas*, *Reyranella*, *Sulfurospirillum*, *Thermaerobacter*, *Thermus*, unassigned bacteria from the Sphingomonadaceae family, and unassigned Thermoplasmata. ASVs assigned to *Aliivibrio* and *Pseudoalteromonas* were significantly underrepresented in the drill cores compared to the seawater samples. Although the latter genera are generally detected in seawater, they were significantly overrepresented in the borehole fluids collected before drilling, indicating that marine microorganisms (or their DNA) naturally infiltrate the subsurface basaltic deposits. Other overrepresented ASVs in the borehole fluids, as compared to the drill core samples, included unassigned Acetothermiia, BRH-c8a (Desulfallas-Sporotomaculum), *Ruegeria*, *Rhodobaca*, unassigned RBG-16-55-12 (Actinobacteriota), *Conexibacter*, *Desulfosporosinus*, *Desulfatiglans*, unassigned Ammonifexales, and Ammonificaceae, among others (Supplementary Table S5). Archaeal taxa that proved to be overrepresented in the borehole fluids included unassigned Thermoplasmata, Hydrothermarchaeales, Syntrophoarchaeaceae, and the genus *Thermococcus*. Using the archaeal primer sets, unassigned Thermoplasmatota and Bathyarchaeia were added to the list of overrepresented archaeal taxa detected using the universal primer set (Figure 5, Supplementary Table S5).

Comparing the four categories of drill cores, ASVs overrepresented in the drill core samples from the subaerial tuff cone (DC_1; 23–35m b.s.; 20–36°C in 2017) were assigned to the genera *Thermaerobacter*, *Thioalkalimicrobium*, *Salinarimonas*, *Marinobacter*, *Nioella*, *Ectothiorhodospira*, *Polaribacter,* and unassigned Limnochordaceae (Figure 5). No ASVs were significantly overrepresented in drill core samples from the zone of daily intertidal fluctuations (DC_2; 44–65m b.s.; 59–101.5°C in 2017) compared with the other categories of drill core samples. Although one ASV assigned to *Geobacillus* is significantly overrepresented in the drill core samples from the hydrothermal temperature maximum (DC_3; 78–139m b.s.; 97–124°C in 2017), this is similar to the drill core samples located below the zone of seawater inflow (DC_4; 157–181m b.s.; 37–84°C in 2017). These samples shared significant ASVs with the seawater samples, including *Flavicella*, NS4 marine group from the Flavobacteriaceae family, *Synechococcus* CC9902, *Planktomarina,* and *Sulfitobacter*. The presence of these taxa further suggests an infiltration of marine microorganisms associated with the infiltration of cool seawater at 145–155m depth, if we presume that no drilling fluid contamination remains.

### Possible Biotic Structures Revealed by SEM Images

To determine whether the detected DNA sequences could be derived from planktonic cells in interstitial pore fluids or, alternatively, attached to surfaces of the lapilli tuff and to investigate their organization on the basaltic substrate, SEM studies were undertaken on six drill core samples, differing in depth (C4, C9, C22, C49, C55, and C65).

**Figure 6:**
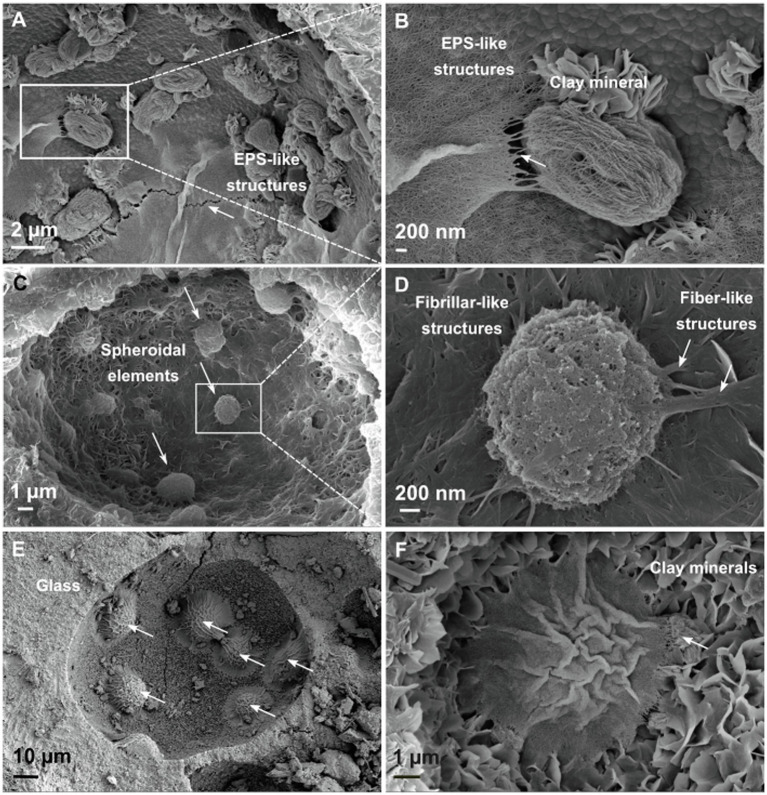
Scanning electron micrographs of Surtsey tephra surfaces in drill core samples. **(A,B)** Platey clay mineral and EPS-like structures, 65m b.s., sample C22. **(C,D)** Vesicle surfaces in altered basaltic glass are covered by a net of fibrillar-like structures that appears to be attached to spheroidal elements, 32m b.s, sample C9. **(E,F)** Wrinkled dome structures, or mounds, are covered by a network of filaments, 65m b.s, sample C22.

All six lapilli tuff samples are porous with high water absorption (Jackson et al., 2019b). Vesicles with spherical shapes 10–80μm in diameter occur in all samples, and platy clay mineral structures, with flower petal morphologies, indicate alteration of the original volcanic glass (Jakobsson and Moore, 1986; Figure 6B). The distribution of microstructures varies with sampling depth; here, we focus on instructive samples at 32m b.s (C9) and 65m b.s (C22). Some vesicle surfaces are covered by a net of thin filaments with an approximate diameter of 10nm. These features are closely intertwined with one other and could possibly correspond to extracellular polymeric substances-like (EPS) structures (Thorseth et al., 2001, 2003; Sudek et al., 2017), perhaps representing a “relic” of past biofilm activity (Figures 6A,B). In other vesicles, spheroidal elements correspond to putative microbial cells 2μm in diameter (Figure 6C) as previously described (Thorseth et al., 2001, 2003). Furthermore, these vesicles are covered by fibrillar-like microstructures (Figure 6D), that appear texturally different from the EPS-like structures (Figure 6B). They occur as nets of oriented fibers with ramifications; some seem to be attached to the spheroidal structures. Energy dispersive X-ray analysis on the spheroidal elements does not detect carbon or phosphate but, instead, detects magnesium, aluminum, and iron. They therefore seem to have an inorganic origin.

Wrinkled dome structures are observed in many vesicles at 65m b.s (Figure 6E) with a diameter size of 10–20μm. A network of intercrossed filaments covers the surface of the dome or mound (Figure 6F). Their wrinkled appearance could be explained by the sample preparation for SEM images, which includes gradual dehydration. EDX analyses investigate the chemical signature of the wrinkled dome structures (Supplementary Figure S7). The superposition of the spectra corresponding to the adjacent clay mineral(s) (Supplementary Figure S7, red area) and the wrinkled dome structures (Supplementary Figure S7, blue area) shows enrichment in carbon, oxygen, sodium, phosphate, and calcium in the crinkled ridge of the mound. These could possibly indicate elements associated with the cells and biomass of a biofilm.

## Discussion

Microbial colonization by pioneer communities can occur within months on freshly deposited erupted volcanic rocks in geothermally active environments (e.g., Konhauser et al., 2002; Kelly et al., 2014). The Surtsey geothermal system offers a highly unusual site to study rapid microbial colonization in newly formed oceanic crust at <100years and>50°C. Results of the analysis described in Figures 3, 4, and 6 suggest that diverse microbial communities have developed in the Surtsey deposits only 50years after the eruptions ended. Communities attached to the lapilli tuff of drill core samples are significantly different from communities in associated fluids (Figures 3A,B). Similar observations have previously been reported in crustal environments (Ramírez et al., 2019). The Surtsey data suggest that fumaroles, borehole fluids, drill cores, and seawater samples harbor significantly distinct microbial communities (Figures 3B,C). Yet, taxa are shared between these sample types (Figure 3D), suggesting possible transfer of microorganisms among these habitats. The hot fumarole samples from the surface of Surtsey at 82.3 and 85.6°C harbor microbial communities that are more similar to those of the drill core samples located above sea level in the subaerial deposits (e.g., samples C4, C9, 23–35m b.s.; Figure 2B). This suggests that some species could disperse through the subaerial tuff cone above sea level up to the surface through fumarole activity (Tin et al., 2011). Excluding all the drilling fluid ASVs contaminants, microbial communities detected in the seawater samples are closer in community structure to the drill core samples from the zone of seawater inflow (sample C52, 148m b.s., Figure 2B) and to the samples in proximity to the seafloor (sample C65, 181m b.s., Figure 2B). This suggests that microorganisms from the seawater surrounding the island (i) infiltrate the submarine tephra deposits, following the seawater inflow at about 148m b.s (Jakobsson and Moore, 1986; Jackson et al., 2019b; Kleine et al., 2020), and (ii) occupy the deeper zone of poorly consolidated tuff through indirect infiltration of the seawater and its circulation *via* the seafloor. The ability of some species to expand across ecosystems and adapt to new environmental conditions (Sriswasdi et al., 2017) has possibly driven the subsurface colonization of the Surtsey volcano. Species that cannot survive might serve as supply of fermentable organic molecules for the subsurface heterotrophic microorganisms (Li et al., 2020).

### Assessment of contamination by Drilling Fluid

The problem of subsurface sample contamination during drilling operations is well-known (Barton et al., 2006; Lever et al., 2006; Kieft, 2010; Santelli et al., 2010; Sheik et al., 2018) since drilling operations require the use of circulating fluid that inevitably infiltrates into the drill core. As a result, the recovery of uncontaminated rock is nearly impossible (Friese et al., 2017; Kallmeyer, 2017). Conventional methods to assess contamination include the addition of tracer compounds to the drilling fluid. These include fluorescent dyes (Pellizzari et al., 2013), perfluorocarbon tracers (PFT; Lever et al., 2006; Inagaki et al., 2016; Orcutt et al., 2017), and microsphere tracers (Kallmeyer et al., 2006; Yanagawa et al., 2013). The use of these tracer compounds is prohibited on Surtsey due to the protected environmental status of the island (Baldursson and Ingadóttir, 2007). Furthermore, their performance in the Surtsey system would have been quite problematic. Although fluorescent dyes have the advantages of high sensitivity for detection, low cost, and ease of use (Pellizzari et al., 2013), they are unstable at low pH (Zhu et al., 2005) and are also susceptible to degradation in the presence of light (Diehl and Horchak-Morris, 1987). Measurements of Surtsey subsurface borehole fluids extracted from SE-01 in 2016 showed pH values decreasing to 5 at some depths (Kleine et al., 2020). Water tanks containing the drilling fluid were exposed to sunlight during the long Icelandic summer days. The detection of PFTs must be performed immediately on fresh cores because of the high volatility of the PFTs and requires elaborate equipment (Lever et al., 2006) whose transport by helicopter to Surtsey would have been very difficult. Finally, microsphere tracers decompose under high-temperature conditions (Yanagawa et al., 2013) and would not have performed well in the Surtsey hydrothermal system, which currently exceeds 120°C at some depths (Marteinsson et al., 2015; Jackson et al., 2019b). Because of the unique circumstances of drilling on Surtsey, the filtration and UV sterilization of the drilling fluid was the most effective strategy to manage contamination. We then mitigated this strategy with a simple overlap approach that is commonly used in environmental microbiology studies to identify and remove contaminants (Sheik et al., 2018). At the ASVs level, this approach enabled the distinction between marine contaminants from the drilling fluid and possible endemic residents, providing a firm basis for the exploration of microbial life in this extreme and unusual habitat (Figure 2).

The DNA concentration in the drill core samples, measured using a QuBit fluorometer, ranged from 0.2–1.36ng.g^−1^ of basalt, which is in good correlation with other basaltic environments (Fisk et al., 2003; Herrera and Cockell, 2007). Surprisingly, DNA concentration did not correlate with variations in temperature (Supplementary Figure S2a). It does decrease with depth (Supplementary Figure S2b), as previously observed in terrestrial and marine subsurface habitats (Cockell et al., 2012; Ciobanu et al., 2014; Mcmahon and Parnell, 2014), but not significantly. The presence of contaminant DNA in the low-biomass basaltic core samples quite possibly distorts these results. Indeed, low DNA concentration exacerbates issues of external contamination (Sheik et al., 2018). When using 16S rRNA gene sequencing to analyze low-biomass samples, the amplification and sequencing of contaminant DNA are introduced during extraction and library preparation steps, and the risk of well-to-well contamination is high (Minich et al., 2019). The low biomass and the presence of external contamination also influence the sequencing results by affecting the magnitude and biological provenance of analyzed sequences. Therefore, it is of crucial importance to use negative controls and optimal decontamination approaches. The inclusion of bacterial mock communities as extraction and sequencing controls would have been beneficial for the study (Pollock et al., 2018). We emphasize that all samples were handled with extreme care during all steps of the project to minimize, identify and remove contaminants using a combination of experimental and computational methods. The lists of contaminants that have been identified based on the presence/absence of ASVs across samples, their relative abundances, and their taxonomic assignment (Supplementary Tables S1, S2 and S4) should be valuable for future microbial explorations of the oceanic crust. In addition to the low biomass and the presence of external contamination affecting the results, we should keep in mind that the compositional nature of the data has limits. Indeed, the data set reported in this study was obtained by 16S rRNA gene amplicon sequencing; thus, the data are compositional being based on relative abundances, which sum to a constant. Therefore, the analytical approaches (e.g., rarefaction, normalization) and statistical methods (e.g., ANOVA) used to study microbiome data influence the results and can potentially be are subjected to inflated false discovery rates (Gloor et al., 2017). This can lead to the lack of reproducibility among microbiome studies and misinterpretations of microbial community structures (e.g., alpha diversity).

### Putative Inhabitants of the Subsurface

Many of the taxa detected in the SE-01 borehole fluid samples match DNA sequences previously identified in hot springs, hydrothermal vents, and subsurface environments (Figure 5). For example, sequences assigned to the class Acetothermiia closely match with DNA sequences previously found in hydrothermal sediments (GenBank: FM868292). Members of this class were previously detected in anaerobic digesters, hot springs, and other deep biosphere studies (Takami et al., 2012; Jungbluth et al., 2017; Zaitseva et al., 2017; Hao et al., 2018; Korzhenkov et al., 2018). Sequences assigned to the genus BRH-c8a from the family Desulfallas-Sporotomaculum match closely with sequences retrieved from deep groundwater (LC179584) and group with the genus *Desulfotomaculum* that can be found in deep subsurface environments (Sousa et al., 2018; Watanabe et al., 2018). Other sequences belonging to the class Desulfotomaculia are assigned to Ammonifexales and Ammonificaceae; these are related to DNA sequences retrieved from petroleum reservoirs (MF470409). In addition, sequences assigned to *Desulfosporosinus* closely match sequences retrieved from coal formation waters (KC215435), while sequences assigned to *Desulfatiglans* match with sequences found in hydrothermal vents (AB294892). Among the abundant ASVs detected in the borehole fluids, an early-branching, uncultivated actinobacterial clade identified as RBG-16-55-12 in the SILVA database release 138 has been previously detected in serpentinite-hosted systems (Merino et al., 2020). Uncharacterized Thermoplasmata are also detected in serpentinite subsurface deposits (Motamedi et al., 2020), yet numerous sequences assigned to this class from the SE-01 borehole fluid samples showed less than 90% of sequence similarity with the first match on the NCBI Nucleotide collection database (AB327321). These results suggest that all the latter taxa could be endemic in the Surtsey subsurface deposits and perhaps could be common in other oceanic or continental subsurface habitats, as well. Other taxa detected in the SE-01 borehole fluid samples are usually found in seawater, including *Halomonas*, *Pseudoalteromonas*, *Sulfitobacter*, *Aliivibrio,* and *Shewanella* (Figure 5). The presence of marine microorganisms in the SE-01 borehole fluid before new drilling began in 2017 further demonstrates that the infiltration of seawater transports marine microorganisms into the subsurface and its hydrothermal system. These species are also frequently detected in cool basaltic oceanic crust (Templeton et al., 2005; Mason et al., 2007; Zhang et al., 2016), and the question of whether or not these species survive in the Surtsey hydrothermal system could depend on the influence of temperature (Edwards et al., 2012; Baquiran et al., 2016; Ramírez et al., 2019).

Scanning electron microscopy studies of instructive drill core samples reveal possible biotic structures whose size, morphology, and fabrics seem to be consistent with EPS-microcolony complexes that are attached to the basaltic substrate (Figure 6, Supplementary Figure S7; Thorseth et al., 2001, 2003; Nakagawa et al., 2006). Indeed, some taxa detected in the drill core samples could represent endemic inhabitant of the basaltic subsurface of the island, which can be divided into two distinct habitats: the subaerial tuff cone and the submarine tuff deposits. In the subaerial tuff cone (DC_1; 23–35m b.s.; 20–36°C in 2017), the taxa include the genera *Thermaerobacter*, *Thioalkalimicrobium*, *Salinaromonas*, *Marinobacter,* and *Ectothiorhodospira* (Figure 5). *Thermaerobacter* are thermophilic to extremely thermophilic bacteria found in terrestrial and oceanic subsurface environments (Spanevello et al., 2002; Takai et al., 1999). *Thioalkalimicrobium*, *Ectothiorhodospira,* and *Salinarimonas* are detected in alkaline and saline habitats such as soda lakes (Sorokin et al., 2001), saline soil (Cai et al., 2011), or salt mines (Liu et al., 2010). The presence of *Marinobacter* only in the tuff deposits above sea level is curious since it is a common lineage found in marine basaltic habitats, including ridge-flank systems and seamount (Templeton et al., 2005; Zhang et al., 2016). Nevertheless, the presence of these taxa in the subaerial deposits suggests adaptation of the microbial communities to more extreme environmental conditions, such as high temperature, high salt concentration produced by NaCl saturation from seawater evaporation or from alkaline pH produced by basaltic glass dissolution at low fluid-rock ratios (Kleine et al., 2020). No hyperthermophilic species were significantly enriched in the drill core samples from the hydrothermal temperature maximum (DC_3; 78–139m b.s.; 97–124°C in 2017 and 100–141°C in 1979), yet two thermophilic species *Thermus* and *Geobacillus* from the Thermoleovorans group were detected (Figure 5). Based on these results and the taxa detected in the SE-01 borehole fluids, it seems that microbial life may persist in subsurface deposits that have experienced temperatures >120°C, the presumed temperature for functional microbial life. The hydrothermal zone at about 100m b.s. could act as a dispersal barrier that provides an obstacle to the transfer of live cells from the zone of tidal flux and upper submarine deposits to the deeper submarine deposits (e.g., samples C27; 78m b.s. and C52; 148m b.s.). Furthermore, no significant difference exists between the observed microbial communities from the lowermost submarine deposits below the zone of cool seawater inflow (DC_4; 157–181m b.s.; 37–84°C in 2017) and the other submarine deposits (Supplementary Figure S6). Shared taxa mainly include mesophilic Proteobacteria such as unassigned Sphingomonadaceae, *Glaciecola*, *Arenicella*, *Oceaniserpentilla*, and *Piscinibacter* that are commonly found in marine habitats. In addition, *Candidatus* Nitrosopumilus and unassigned Thermoplasmata from the Marine Group II were detected in great abundance (Figure 5). Those taxa have been previously detected in marine basalts and deep seawater circulation through oceanic crust (Mason et al., 2007; Singer et al., 2015; Suzuki et al., 2020; Bergo et al., 2021). Although the deep ocean is typically enriched in archaeal cells, other marine environments usually show a dominance of bacterial lineages (Karner et al., 2001). This has been previously observed in the basaltic crust. For example, Gammaproteobacteria and Alphaproteobacteria are detected in great abundance in the seafloor basaltic glass of the East Pacific Rise (Santelli et al., 2008, 2009), the Arctic spreading ridges (Lysnes et al., 2004), altered basalts from the Hawaiian Loihi Seamount (Templeton et al., 2005; Santelli et al., 2008; Jacobson Meyers et al., 2014), and the Mid-Atlantic Ridge (Rathsack et al., 2009; Mason et al., 2010). Deltaproteobacteria, Firmicutes, Gammaproteobacteria, and Bacteroidetes are also detected in great abundance in the Juan de Fuca Ridge flank and the Costa Rica Rift (Nigro et al., 2012; Jungbluth et al., 2013, 2014). Our results support the dominance of bacterial lineages in marine basalts.

While most taxa could be identified at a low taxonomic rank, many others were not assigned to a known genus, family, or even order. For example, many sequences detected in this study fell into unknown clades, including Acetothermiia, Ammonifexales, Bacilli, RBG-16-55-12 from the phylum Actinobacteriota, Sphingomonadaceae, Limnochordaceae, and Saccharimonadales, among others. Likewise, many archaeal sequences could not be identified further than the phylum level, including unassigned Thermoplasmatota and Halobacterota, or the class level, including unassigned Thermoplasmata and Bathyarchaeia. Hence, the subsurface biosphere of Surtsey lapilli tuff and tephra, as well as fluids, could have high potential for discoveries of new microbial clades.

### Metabolic Potential

**Figure 7:**
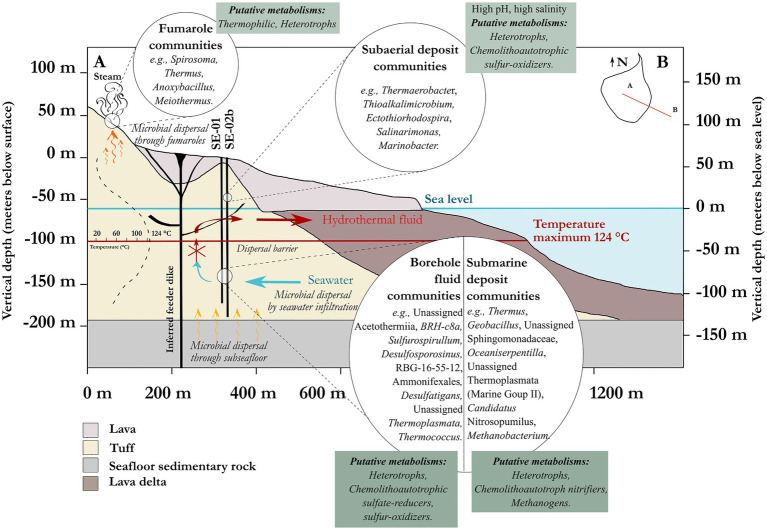
Overview of the putative metabolisms and microbial dispersion scenario of the subsurface of Surtsey volcano.

The metabolic potential of the endemic subsurface microbial communities can be discussed with some degree of certainty, while remaining mindful of the difficulties inherent to identifications based on 16S rRNA gene sequence analysis. To support our hypotheses, predictive functional analyses were performed using PICRUSt2. A few MetaCyc pathways were selected to represent the functional potential of the bacterial and archaeal communities for carbon, sulfur, nitrogen, and methane metabolism (Supplementary Figures S8, S9). Many genera reported in this study belong to taxonomic clades with known metabolisms that are involved in both heterotrophy and chemolithoautotrophy (Figure 7). The presence of putative sulfate-reducing bacteria strongly suggests a potential for active sulfate reduction, including *Desulfosporosinus*, *Desulfatiglans,* and the *Desulfotomaculia* class, among others. In addition, sulfur oxidizers were detected, such as the genera *Thioalkalimicrobium*, *Sulfurospirillum*, *Sulfurimonas*, *Ectothiorhodospira,* and *Sulfurihydrogenibium*. The observation of these bacterial taxa possibly involved in sulfate reduction and sulfur oxidation suggests an active sulfur cycle in the subsurface of Surtsey (Supplementary Figure S8), as has previously been reported in similar ecosystems (Bach and Edwards, 2003; Lever et al., 2013; Suzuki et al., 2020). This is further reinforced by the detection of archaea possibly involved in the sulfur cycle, such as *Thermococcus*, *Pyrococcus*, and *Archaeoglobus*. Also, characterized members of the Thermoplasmatales are typically involved in sulfur cycling (Barton et al., 2014; Arce-Rodríguez et al., 2019). These taxa coincide with deposits that contain sulfate minerals, principally anhydrite and gypsum (Jakobsson and Moore, 1986; Kleine et al., 2020; Prause et al., 2020).

In addition, the genus *Methanobacterium* from the order Methanobacteriales dominates the archaeal sequences of drill core sample C55, at 157m depth (Figure 3). The MetaCyc pathways detected in this sample using the archaeal data set were mainly involved in methane metabolism (e.g., methanogenesis from H_2_ and CO_2_, coenzyme B biosynthesis, coenzyme M biosynthesis I; Supplementary Figure S9). One taxon from the same order of Methanobacteriales was previously reported to dominate the SE-01 borehole fluid microbial communities sampled in 2009 at similar depth (Marteinsson et al., 2015). *Methanobacterium* spp. grows by reducing carbon dioxide to methane and uses molecular hydrogen as the electron donor (Kern et al., 2015). Hence, these taxa could play an important role as primary producers in this ecosystem, at least at certain depths. The possible occurrence of an active methane cycle is supported by the presence of other methanogens (e.g., Methanosarcinia (Syntrophoarchaeaceae), Methanomassiliicoccales (*Methanothermus*, *Methanoregula*), as well as methanotrophic (e.g., *Methylocella*, Methylacidiphilaceae), and methylotrophic (e.g., *Hansschlegelia*, *Methylopila*, Methylophilaceae (OM43 clade), *Methylophaga*) bacteria, despite their relatively low abundances. The observation of genera such as *Geobacter*, *Rhodoferax*, *Marinobacter*, *Shewanella,* and *Ferruginibacter*, as well as *Hydrogenophilus* and *Hydrogenophaga,* could indicate that iron and hydrogen are electron donors within the ecosystem, as previously reported for other subsurface habitats (Bach and Edwards, 2003; Bach, 2016; Zhang et al., 2016). In addition, putative ammonium-oxidizing archaea belonging to the Marine Group II and *Candidatus* Nitrosopumilus suggest an ability to transform nitrogen compounds as previously reported in the oceanic crust (Daae et al., 2013; Orcutt et al., 2015; Jørgensen and Zhao, 2016; Zhao et al., 2020).

## Conclusions

The 1979 and 2017 drilling projects at Surtsey volcano provide a rare opportunity to explore the subsurface microbial diversity of a very young basaltic island associated with an active hydrothermal-seawater system in newly formed oceanic crust. A cored borehole dedicated to microbiology research in the 2017 drilling operation probes a low biomass but highly diverse habitat that hosts bacterial and archaeal clades, including extremophiles, that have been previously detected in other terrestrial and marine environments. Many clades, however, fall into as-yet-unknown lineages. The 16s rRNA gene amplicon sequencing data provide insights into diverse sources of microbial colonization in newly formed oceanic crust, with potential dissemination from the deep subsurface, surrounding seawater, and surface ecosystems. The island of Surtsey may thus be regarded as a porous, sponge-like basaltic structure that absorbs cells from the surrounding environments and selects microorganisms that can adapt to the extreme environmental conditions that exist within the volcano. The data also provide a baseline for long-term observations of the microbial communities inhabiting the subsurface of Surtsey and their temporal succession amid a changing hydrothermal environment, in which poorly consolidated tephra lithify to form well-consolidated lapilli tuff. Further research, including metagenomic sequencing, will increase our knowledge of the metabolism and function of the microbiome in this very young basaltic environment.

## Data Availability Statement

The data sets (accession number ERP126178) for this study can be found in the European Nucleotide Archive (ENA) at EMBL-EBI (https://www.ebi.ac.uk/ena/browser/view/PRJEB42339).

## Author Contributions

VM (PI of the microbiology part of drilling operation), MJ, and MG conceived the study. PB, AK, VM, and members of the SUSTAIN onsite, science teams conducted field operations and sampling. PB and PV processed the samples and conducted the molecular biology experiments. PB, PV, and SK performed the data analysis. PB wrote the original draft. All authors took part in writing the manuscript.

## Funding

Sample collection and sample processing were funded with a grant of excellence from the Icelandic Science fund, ICF-RANNÍS IceSUSTAIN (163083-051). The drilling operation was funded with International Continental Scientific Drilling Program (ICDP) through the SUSTAIN project and the IceSUSTAIN grant. A doctoral student grant from RANNÍS (206582-051) was attributed to PB.

## Code Availability Statement

The pipeline used to analyze the data set reported in this manuscript can be found at https://benjjneb.github.io/dada2/tutorial.html. The code used to identify contaminants can be found at https://benjjneb.github.io/decontam/vignettes/decontam_intro.html, and the code used to build the figures can be found in the supplementary material under the section code.

## Conflict of Interest

The authors declare that the research was conducted in the absence of any commercial or financial relationships that could be construed as a potential conflict of interest.

## Publisher’s Note

All claims expressed in this article are solely those of the authors and do not necessarily represent those of their affiliated organizations, or those of the publisher, the editors and the reviewers. Any product that may be evaluated in this article, or claim that may be made by its manufacturer, is not guaranteed or endorsed by the publisher.

## Footnotes

^1^ https://github.com/benjjneb/decontam
