# Supplementary material for: Basalt-Hosted Microbial Communities in the Subsurface of the Young Volcanic Island of Surtsey, Iceland
Source: Front Microbiol. 2021 Sep 29;12:728977. doi: 10.3389/fmicb.2021.728977 (PMC8513691; doi:10.3389/fmicb.2021.728977)
Supplement: Supplementary file 1 [file Data_Sheet_1.docx]

Supplementary Material

**Table of contents**

[1 Supplementary Method 2](#_Toc80719075)

[2 Supplementary Figures and Tables 3](#_Toc80719076)

[2.1 Supplementary Figures 3](#_Toc80719077)

[2.2 Supplementary Tables 9](#_Toc80719078)

[3 Code 58](#_Toc80719079)

[3.1 Figure 2 59](#_Toc80719080)

[3.2 Figure 3a 59](#_Toc80719081)

[3.3 Figure 3b 60](#_Toc80719082)

[3.4 Figure 3c 60](#_Toc80719083)

[3.5 Figure 3d 61](#_Toc80719084)

[3.6 Figure 4. 61](#_Toc80719085)

[3.7 Figure 5. 62](#_Toc80719086)

[3.8 Figure S1. 68](#_Toc80719087)

[3.9 Figure S2. 68](#_Toc80719088)

[3.10 Figure S3. 68](#_Toc80719089)

[3.11 Figure S4. 68](#_Toc80719090)

[3.12 Figure S5. 69](#_Toc80719091)

[3.13 Figure S6. 69](#_Toc80719092)

# Supplementary Method

Correlation tests were performed using the function ‘ggscatter’ from ggpubr package with the p-value added to the plot. To compute summary statistics by groups (mean and standard deviation), the function ‘get_summary_stats’ from the rstatix R package was used.

The function ‘estimate_richness’ was used to obtain standard alpha diversity estimates, including the number of observed ASVs by samples, Shannon and InvSimpson diversity index. Differences in α diversity values between sample types were tested using the function ‘aov’ from the package stats, which perform analysis of variance (ANOVA) and Tukey's HSD (honestly significant difference) test was performed using the function ‘glht’ from the package multcomp to return what sample type differ from each other (Hothorn et al., 2008). P values <0.05 were considered statistically significant (Chambers et al., 2017).

For β diversity assessment between the sample types, the data were normalized using the ’rarefy_even_depth’ function prior to performing a Non-metric Multidimensional Scaling (NMDS) ordination on Bray-Curtis dissimilarities using the functions ‘ordinate’ and ‘plot_ordination’. Permutational Multivariate Analysis Of Variance using distance matrices (PERMANOVA) was achieved using the ‘adonis’ function with 999 permutations. Distance matrix was calculated using the function ‘distance’ (method: Bray-Curtis) and the ‘pairwise.adonis’ function from R package pairwiseAdonis was used to show significant differences among sample types (Martinez Arbizu, 2020). The function ‘stat_ellipse’ was used to compute normal confidence ellipses (Fox and Weisberg, 2011).

Bar plots were made using ggplot2 (Wickham, 2011). Venn diagram was achieved using venneuler R package. Canonical correspondence analysis (CCA) using the ‘cca’ function (with 999 permutations) was used to assess the dissimilarity between the microbial communities of the drill core samples (ASVs-level data) and ‘envfit’ function was used to assess significant environmental factors structuring the species distribution in the ordination space. Both depth and temperature were significant variables (p < 0.05).

Differential expression analysis of differentially present ASVs between sample types (fumarole, borehole fluid, drill core and seawater samples) and between categories of drill cores (DC_1, DC_2, DC_3 and DC_4) were calculated using DESeq2 approach with a False Discovery Rate (FDR) cutoff of 0.01 (Love et al., 2014). ASVs showing a p-value < 0.01 were considered indicative of statistical significance (Table S5).

In parallel, samples were agglomerated by sample types and categories of drill cores (fumarole, borehole fluid, seawater samples, DC_1, DC_2, DC_3 and DC_4) using ‘merge_samples’ function and the sample counts was transformed in taxa relative abundance using ‘transform_sample_counts’ function. Only significant ASVs identified using DESeq2 were extracted from the latter otu_table and lower taxonomic level were showed in a bubble plot with the original relative abundance of the significant ASVs (Figure 5).

Rarefaction curve was performed using the function ‘rarefy’ from vegan R package.

# Supplementary Figures and Tables

## Supplementary Figures


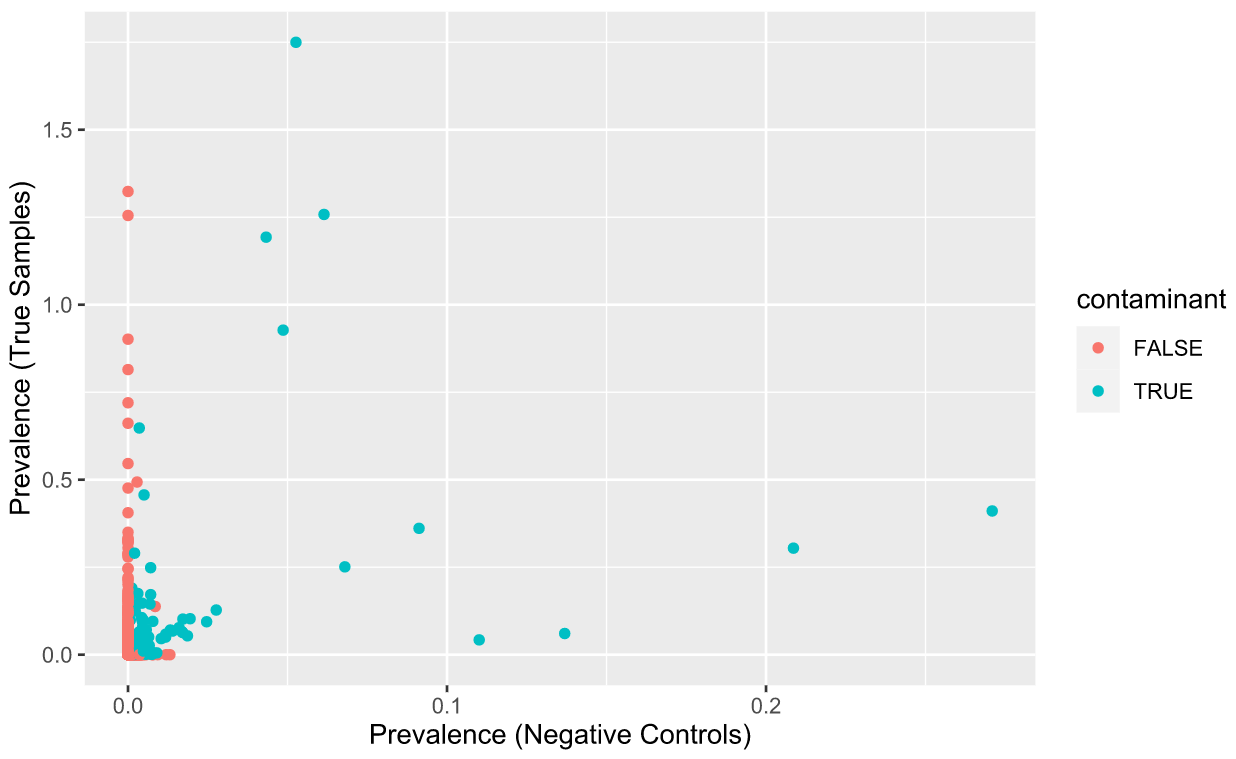


**Figure S1.** Identification plot of the taxa prevalence in positive and negative samples.


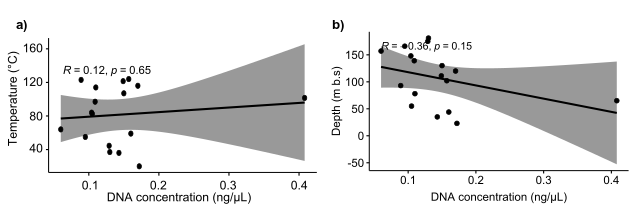


**Figure S2.** Correlation tests. DNA concentration from the 17 drill core samples using QuBit measurements (ng/µL) function of a) *in-situ* temperature and b) sampling depth (m b.s.).


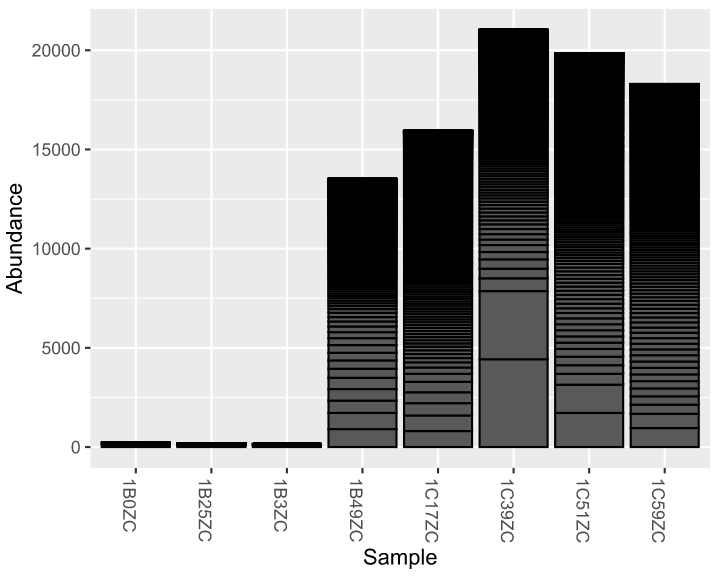


**Figure S3.** Bar plot of reads and ASVs number function of the drilling fluid samples.


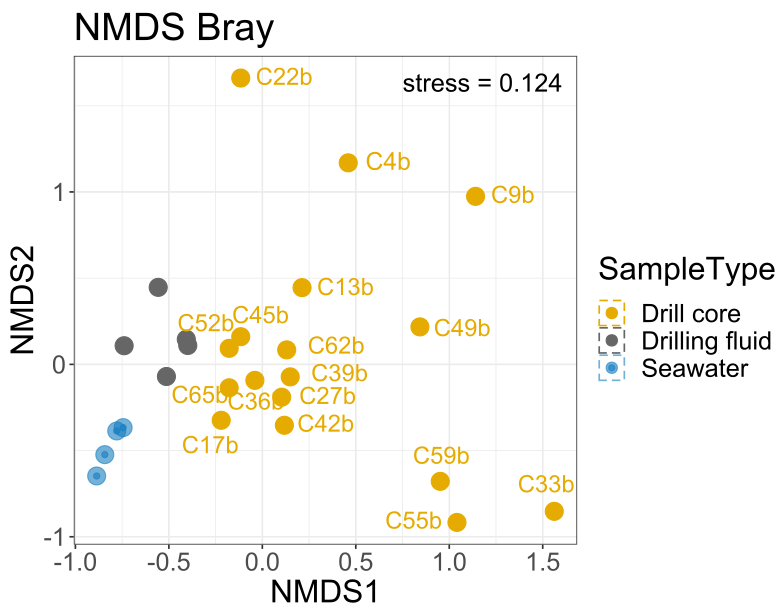


**Figure S4.** A non-metric Multidimensional Scaling (NMDS) ordination plot conducted using Bray-Curtis dissimilarity metrics (stress value = 0.124) of the drilling fluid, seawater samples and drill core samples.

**
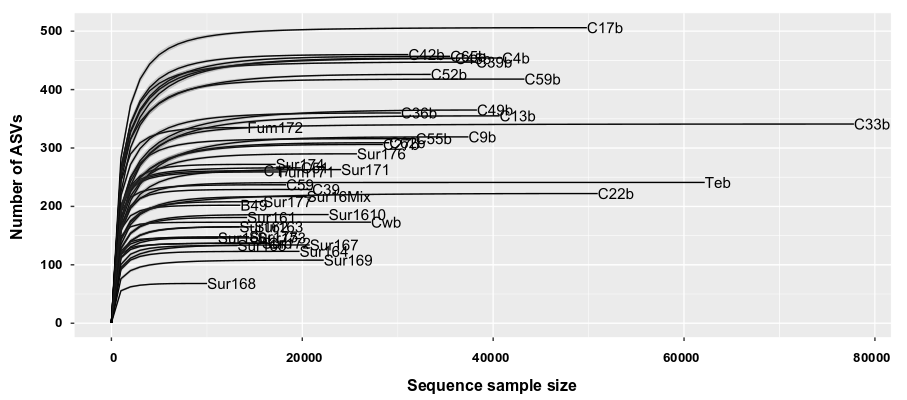
**

**Figure S5.** Rarefaction curves before data manipulation. The sequencing provided sufficient reads to capture the total richness of the samples.


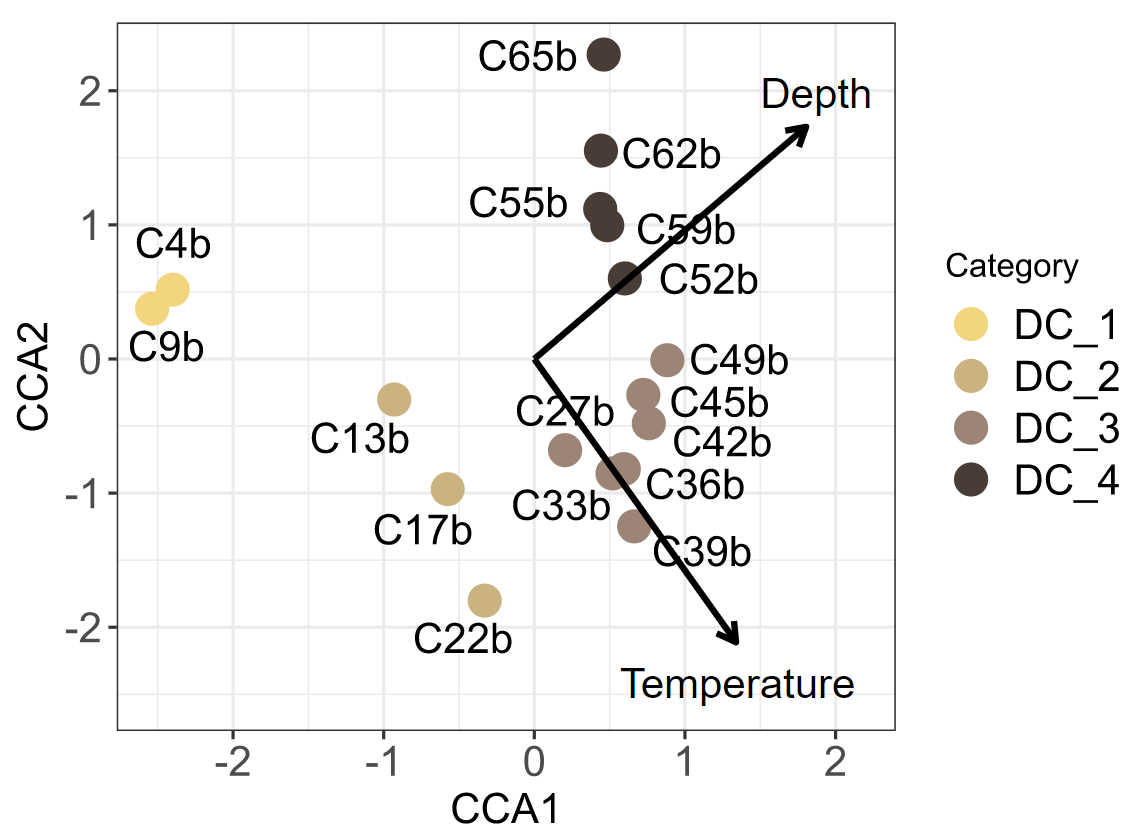


**Figure S6.** Correlation between the microbial community of the drill core samples and environmental variables using envfit. The two axes of the canonical correspondence analysis (CCA) display microbial community of the samples (dots; DC: Drill cores) and variables (arrows) with significant correlation (p < 0.05). Arrows show the level and direction of impact of the significant factors depth and temperature, which are linked.


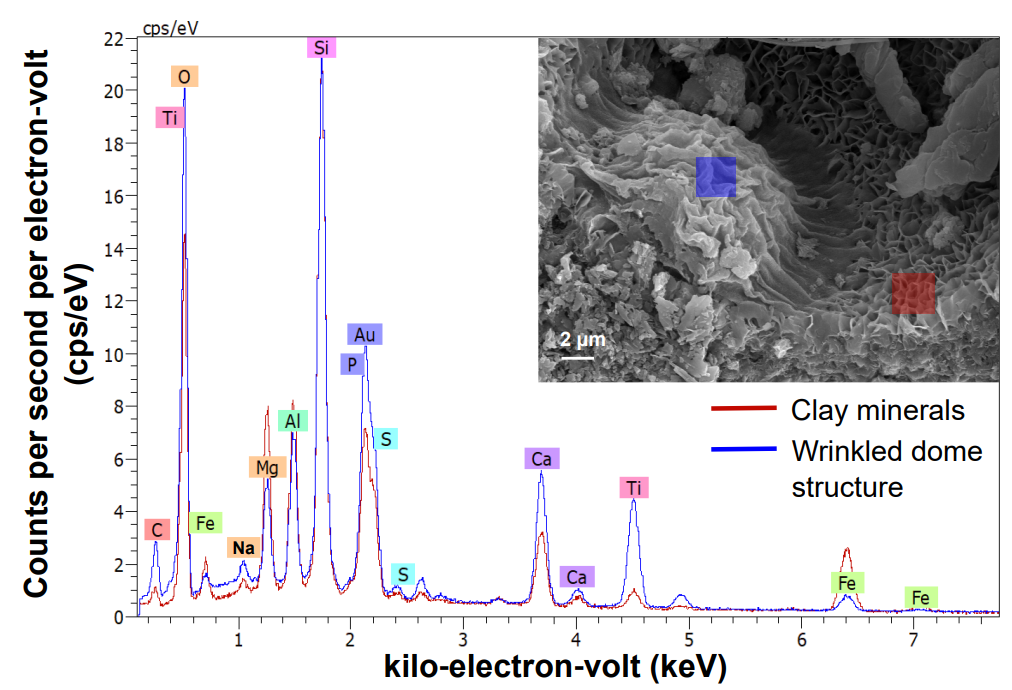


**Figure S7.** SEM-EDX analyses from wrinkled dome structure and altered vesicle surface, 65 m b.s, Surtsey drill core sample C22. The SEM-SE image shows a wrinkled dome structure (blue square) and adjacent clay mineral(s) (red square) subjected to EDX analysis, which indicates an enrichment of C, O, Na, P and Ca in the net of filaments covering the mound. cps/Ev: counts per second per electron‐volt, keV: kilo‐electron‐volt.


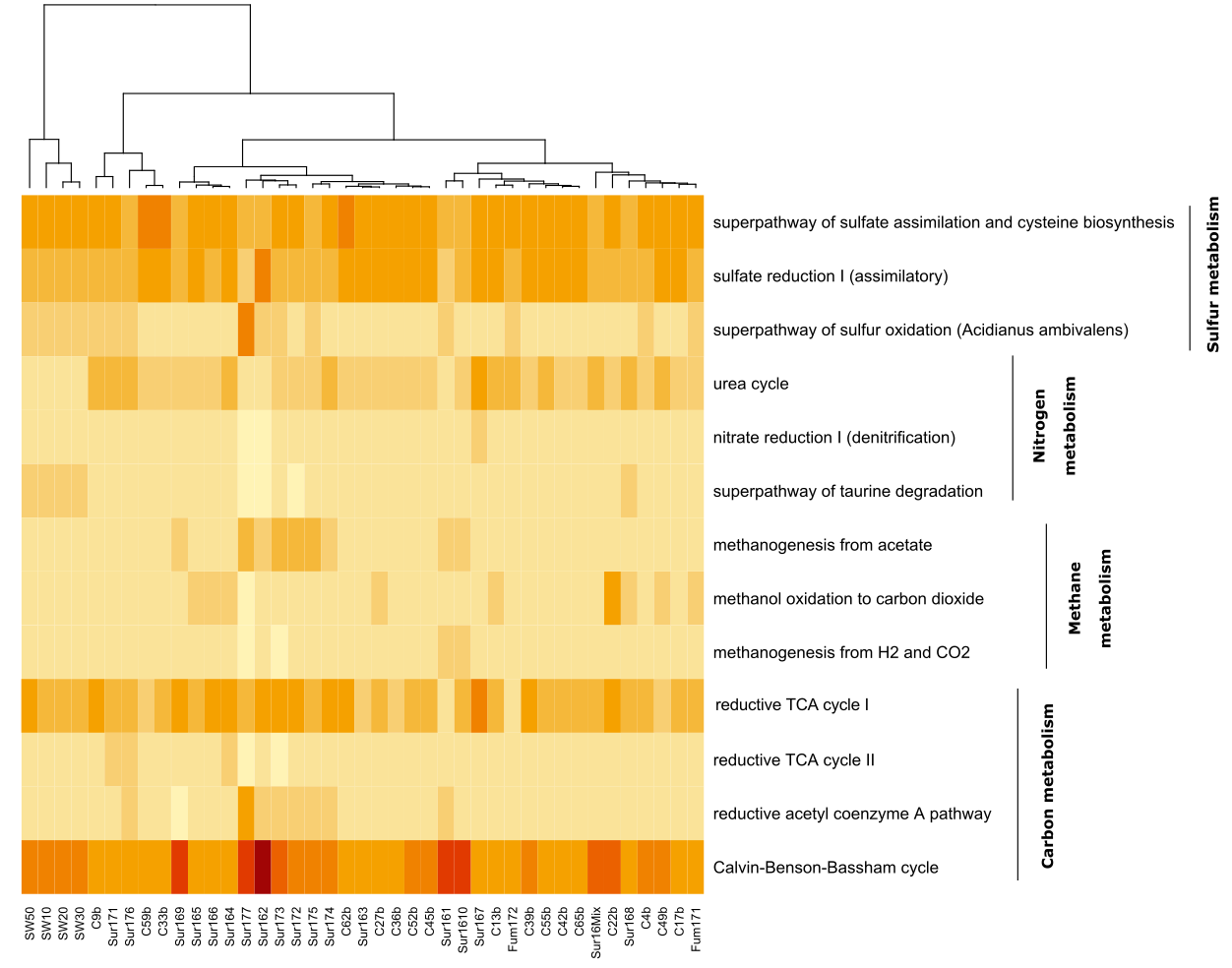


**Figure S8.** Predicted pathways for sulfur, carbon, nitrogen and methane metabolism from the universal dataset (PICRUSt2).


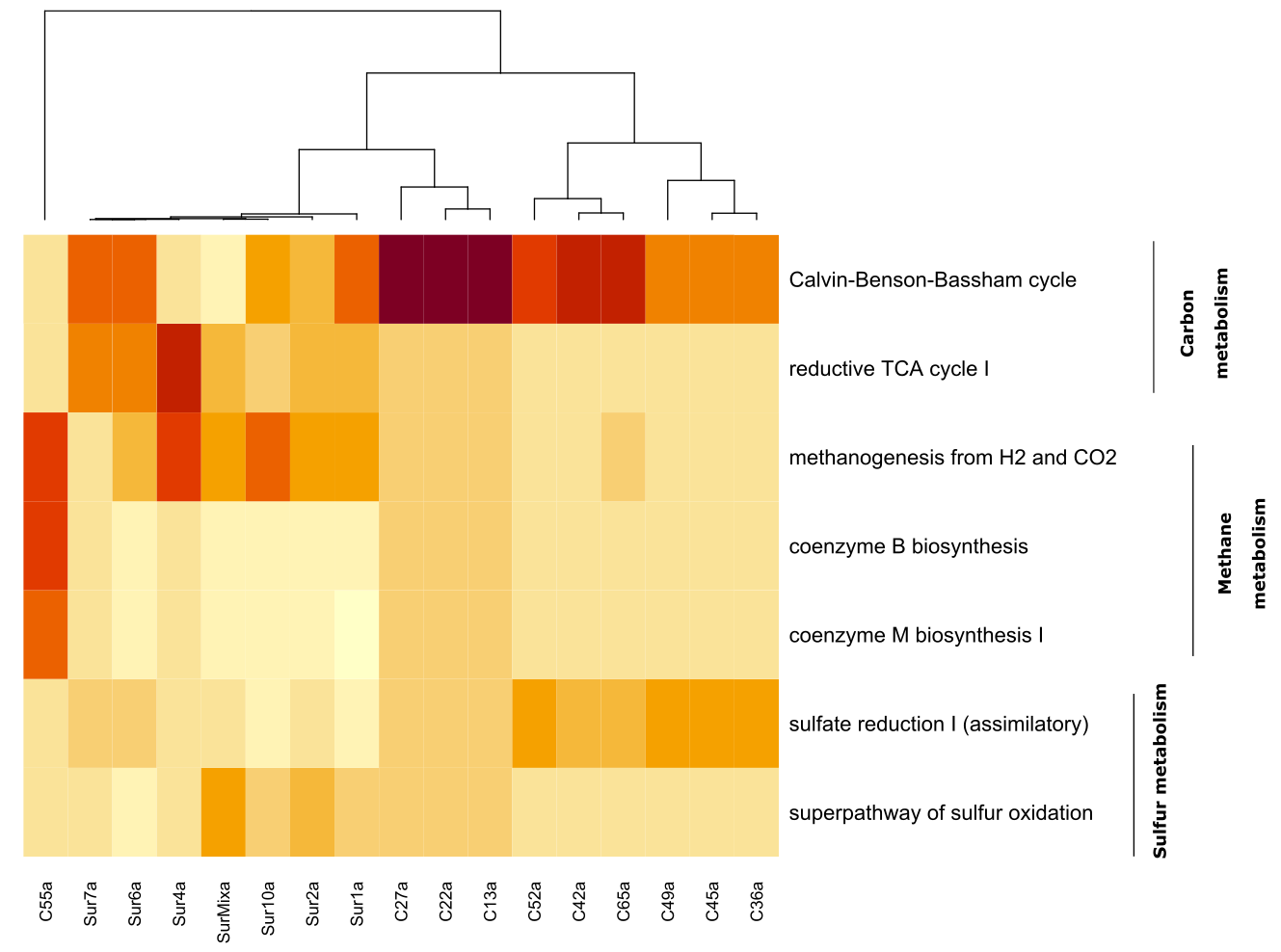


**Figure S9.** Predicted pathways for sulfur, carbon and methane metabolism from the archaeal dataset (PICRUSt2).

## Supplementary Tables

**Table S1.** Contaminant ASVs identified using Decontam prevalence method (threshold value of 0.5 [160]). NA., Not assigned.

| Kingdom | Phylum | Class | Order | Family | Genus |
| --- | --- | --- | --- | --- | --- |
| Bacteria | Firmicutes | Clostridia | Clostridiales | Clostridiaceae | Clostridium sensu stricto 10 |
| Bacteria | Campilobacterota | Campylobacteria | Campylobacterales | Sulfurimonadaceae | Sulfuricurvum |
| Bacteria | Proteobacteria | Alphaproteobacteria | Sphingomonadales | Sphingomonadaceae | Sphingomonas |
| Bacteria | Firmicutes | Negativicutes | Veillonellales-Selenomonadales | Veillonellaceae | Veillonella |
| Bacteria | Bacteroidota | Bacteroidia | Cytophagales | Hymenobacteraceae | Hymenobacter |
| Bacteria | Proteobacteria | Gammaproteobacteria | Pseudomonadales | Moraxellaceae | Acinetobacter |
| Bacteria | Caldisericota | Caldisericia | Caldisericales | Caldisericaceae | Caldisericum |
| Bacteria | Firmicutes | Bacilli | Lactobacillales | Streptococcaceae | Streptococcus |
| Bacteria | Proteobacteria | Gammaproteobacteria | Pseudomonadales | Moraxellaceae | Acinetobacter |
| Bacteria | Actinobacteriota | Actinobacteria | Micrococcales | Micrococcaceae | Rothia |
| Bacteria | Proteobacteria | Gammaproteobacteria | Burkholderiales | Comamonadaceae | Curvibacter |
| Bacteria | Firmicutes | Negativicutes | Veillonellales-Selenomonadales | Veillonellaceae | Veillonella |
| Bacteria | Chloroflexi | Gitt-GS-136 | NA | NA | NA |
| Bacteria | Campilobacterota | Campylobacteria | Campylobacterales | Campylobacteraceae | Campylobacter |
| Bacteria | Bacteroidota | Bacteroidia | Sphingobacteriales | Sphingobacteriaceae | Pedobacter |
| Bacteria | Proteobacteria | Gammaproteobacteria | Burkholderiales | Comamonadaceae | Pelomonas |
| Bacteria | Proteobacteria | Gammaproteobacteria | Vibrionales | Vibrionaceae | Photobacterium |
| Bacteria | Firmicutes | Bacilli | Lactobacillales | Streptococcaceae | Streptococcus |
| Bacteria | Actinobacteriota | Actinobacteria | Actinomycetales | Actinomycetaceae | Actinomyces |
| Bacteria | Actinobacteriota | Actinobacteria | Corynebacteriales | Corynebacteriaceae | Corynebacterium |
| Bacteria | Proteobacteria | Gammaproteobacteria | Enterobacterales | NA | NA |
| Bacteria | Firmicutes | Bacilli | Staphylococcales | Staphylococcaceae | Staphylococcus |
| Bacteria | Armatimonadota | Fimbriimonadia | Fimbriimonadales | Fimbriimonadaceae | NA |
| Bacteria | Firmicutes | Bacilli | Lactobacillales | Carnobacteriaceae | Carnobacterium |
| Bacteria | Proteobacteria | Gammaproteobacteria | Pasteurellales | Pasteurellaceae | Haemophilus |
| Bacteria | Proteobacteria | Gammaproteobacteria | Enterobacterales | Enterobacteriaceae | Raoultella |
| Bacteria | Actinobacteriota | Actinobacteria | Corynebacteriales | Nocardiaceae | Rhodococcus |
| Bacteria | Proteobacteria | Alphaproteobacteria | Rhizobiales | Beijerinckiaceae | Methylobacterium-Methylorubrum |
| Bacteria | Proteobacteria | Gammaproteobacteria | Vibrionales | Vibrionaceae | Photobacterium |
| Bacteria | Fusobacteriota | Fusobacteriia | Fusobacteriales | Leptotrichiaceae | Leptotrichia |
| Bacteria | Actinobacteriota | Actinobacteria | Micrococcales | Micrococcaceae | Micrococcus |
| Bacteria | Actinobacteriota | Actinobacteria | Propionibacteriales | Propionibacteriaceae | Cutibacterium |
| Archaea | Crenarchaeota | Nitrososphaeria | Nitrosopumilales | Nitrosopumilaceae | NA |
| Bacteria | Campilobacterota | Campylobacteria | Campylobacterales | Campylobacteraceae | Campylobacter |
| Bacteria | Actinobacteriota | Actinobacteria | Micrococcales | Microbacteriaceae | Microbacterium |
| Bacteria | Actinobacteriota | Actinobacteria | Propionibacteriales | Propionibacteriaceae | Cutibacterium |
| Bacteria | Proteobacteria | Alphaproteobacteria | Rhizobiales | Xanthobacteraceae | Afipia |
| Bacteria | Proteobacteria | Gammaproteobacteria | Enterobacterales | Enterobacteriaceae | Klebsiella |
| Bacteria | Firmicutes | Bacilli | Lactobacillales | Enterococcaceae | Enterococcus |
| Bacteria | Cyanobacteria | Vampirivibrionia | Obscuribacterales | Obscuribacteraceae | NA |
| Bacteria | Proteobacteria | Gammaproteobacteria | Pseudomonadales | Moraxellaceae | Acinetobacter |
| Bacteria | Proteobacteria | Alphaproteobacteria | Sphingomonadales | Sphingomonadaceae | Sphingomonas |
| Bacteria | Deinococcota | Deinococci | Deinococcales | Deinococcaceae | Deinococcus |
| Bacteria | Actinobacteriota | Actinobacteria | Propionibacteriales | Nocardioidaceae | Aeromicrobium |
| Bacteria | Proteobacteria | Gammaproteobacteria | Pseudomonadales | Moraxellaceae | Acinetobacter |
| Bacteria | Proteobacteria | Gammaproteobacteria | Pseudomonadales | Moraxellaceae | Enhydrobacter |
| Bacteria | Actinobacteriota | Actinobacteria | Corynebacteriales | Corynebacteriaceae | Corynebacterium |
| Bacteria | Proteobacteria | Gammaproteobacteria | Pseudomonadales | Pseudomonadaceae | Pseudomonas |
| Bacteria | Firmicutes | Bacilli | Lactobacillales | Streptococcaceae | Streptococcus |
| Bacteria | Proteobacteria | Alphaproteobacteria | Sphingomonadales | Sphingomonadaceae | Sphingomonas |
| Bacteria | Proteobacteria | Gammaproteobacteria | Burkholderiales | Oxalobacteraceae | Massilia |
| Bacteria | Cyanobacteria | Cyanobacteriia | Chloroplast | NA | NA |
| Bacteria | Proteobacteria | Gammaproteobacteria | Burkholderiales | Comamonadaceae | NA |
| Bacteria | Desulfobacterota | Desulfuromonadia | Geobacterales | Geobacteraceae | Geobacter |
| Bacteria | Proteobacteria | Gammaproteobacteria | Pseudomonadales | Pseudomonadaceae | Pseudomonas |
| Bacteria | Proteobacteria | Gammaproteobacteria | Gammaproteobacteria Incertae Sedis | Unknown Family | Acidibacter |
| Bacteria | Acidobacteriota | Blastocatellia | Pyrinomonadales | Pyrinomonadaceae | RB41 |
| Bacteria | Bacteroidota | Bacteroidia | Flavobacteriales | Weeksellaceae | Cloacibacterium |
| Bacteria | Bacteroidota | Bacteroidia | Flavobacteriales | Weeksellaceae | Cloacibacterium |
| Bacteria | Bacteroidota | Bacteroidia | Bacteroidales | Paludibacteraceae | Microbacter |
| Bacteria | Proteobacteria | Gammaproteobacteria | Pseudomonadales | Moraxellaceae | Acinetobacter |
| Bacteria | Proteobacteria | Gammaproteobacteria | Burkholderiales | Comamonadaceae | Polaromonas |
| Bacteria | Proteobacteria | Alphaproteobacteria | Rhizobiales | Beijerinckiaceae | Methylobacterium-Methylorubrum |
| Bacteria | Acidobacteriota | Blastocatellia | NA | NA | NA |
| Bacteria | Actinobacteriota | Actinobacteria | Micrococcales | Bogoriellaceae | Georgenia |
| Bacteria | Firmicutes | Bacilli | Lactobacillales | Lactobacillaceae | Lactobacillus |
| Bacteria | Proteobacteria | Alphaproteobacteria | Rhizobiales | Rhizobiaceae | Allorhizobium-Neorhizobium-Pararhizobium-Rhizobium |
| Bacteria | Firmicutes | Bacilli | Lactobacillales | Lactobacillaceae | Lactobacillus |
| Bacteria | Bacteroidota | Bacteroidia | Flavobacteriales | Flavobacteriaceae | Flavobacterium |
| Bacteria | Proteobacteria | Alphaproteobacteria | Rhizobiales | Xanthobacteraceae | Afipia |
| Bacteria | Proteobacteria | Gammaproteobacteria | Salinisphaerales | Solimonadaceae | Nevskia |
| Bacteria | Proteobacteria | Alphaproteobacteria | Caulobacterales | Caulobacteraceae | Caulobacter |
| Bacteria | Proteobacteria | Alphaproteobacteria | Rhizobiales | Beijerinckiaceae | Methylobacterium-Methylorubrum |
| Bacteria | Actinobacteriota | Actinobacteria | Actinomycetales | Actinomycetaceae | Actinomyces |
| Bacteria | Firmicutes | Clostridia | Eubacteriales | Eubacteriaceae | Acetobacterium |
| Bacteria | Proteobacteria | Alphaproteobacteria | Rhizobiales | Beijerinckiaceae | Methylobacterium-Methylorubrum |
| Bacteria | Actinobacteriota | Actinobacteria | Corynebacteriales | Corynebacteriaceae | Lawsonella |
| Bacteria | Bacteroidota | Bacteroidia | Chitinophagales | Chitinophagaceae | Vibrionimonas |
| Bacteria | Proteobacteria | Gammaproteobacteria | Burkholderiales | Burkholderiaceae | Burkholderia-Caballeronia-Paraburkholderia |
| Bacteria | Actinobacteriota | Actinobacteria | Micrococcales | Micrococcaceae | Rothia |
| Bacteria | Proteobacteria | Alphaproteobacteria | Rhizobiales | Xanthobacteraceae | Bradyrhizobium |
| Bacteria | Proteobacteria | Gammaproteobacteria | Alteromonadales | Alteromonadaceae | Alteromonas |
| Bacteria | Proteobacteria | Gammaproteobacteria | Xanthomonadales | Xanthomonadaceae | Pseudoxanthomonas |
| Bacteria | Firmicutes | Bacilli | Staphylococcales | Gemellaceae | Gemella |
| Bacteria | Firmicutes | Desulfitobacteriia | Desulfitobacteriales | Desulfitobacteriaceae | Desulfosporosinus |
| Bacteria | Firmicutes | Bacilli | Lactobacillales | Streptococcaceae | Streptococcus |
| Bacteria | Proteobacteria | Gammaproteobacteria | Pseudomonadales | Pseudomonadaceae | Pseudomonas |
| Bacteria | Proteobacteria | Alphaproteobacteria | Rhizobiales | Hyphomicrobiaceae | Pedomicrobium |
| Bacteria | Proteobacteria | Alphaproteobacteria | Caulobacterales | Caulobacteraceae | Brevundimonas |
| Bacteria | Proteobacteria | Gammaproteobacteria | Enterobacterales | Enterobacteriaceae | Escherichia-Shigella |
| Bacteria | Proteobacteria | Gammaproteobacteria | Burkholderiales | Comamonadaceae | NA |
| Bacteria | Proteobacteria | Alphaproteobacteria | Caulobacterales | Caulobacteraceae | Caulobacter |
| Bacteria | Actinobacteriota | Actinobacteria | Micrococcales | Micrococcaceae | Rothia |
| Bacteria | Firmicutes | Bacilli | Lactobacillales | Lactobacillaceae | Lactobacillus |
| Bacteria | Proteobacteria | Alphaproteobacteria | Caulobacterales | Caulobacteraceae | NA |
| Bacteria | Firmicutes | Bacilli | Bacillales | Bacillaceae | Bacillus |
| Bacteria | Proteobacteria | Gammaproteobacteria | Pseudomonadales | Pseudomonadaceae | Pseudomonas |
| Bacteria | Proteobacteria | Gammaproteobacteria | Xanthomonadales | Xanthomonadaceae | Stenotrophomonas |
| Bacteria | Proteobacteria | Gammaproteobacteria | Aeromonadales | Aeromonadaceae | Aeromonas |
| Bacteria | Proteobacteria | Alphaproteobacteria | Sphingomonadales | Sphingomonadaceae | Sphingomonas |
| Bacteria | Proteobacteria | Gammaproteobacteria | Burkholderiales | Burkholderiaceae | Ralstonia |
| Bacteria | Bacteroidota | Bacteroidia | Bacteroidales | Porphyromonadaceae | Porphyromonas |
| Bacteria | Firmicutes | Bacilli | Lactobacillales | Carnobacteriaceae | Granulicatella |
| Bacteria | Firmicutes | Bacilli | Staphylococcales | Gemellaceae | Gemella |
| Bacteria | Proteobacteria | Gammaproteobacteria | Burkholderiales | Burkholderiaceae | Ralstonia |
| Bacteria | Proteobacteria | Gammaproteobacteria | Acidithiobacillales | Acidithiobacillaceae | KCM-B-112 |
| Bacteria | Firmicutes | Bacilli | Lactobacillales | Streptococcaceae | Streptococcus |
| Bacteria | Acidobacteriota | Blastocatellia | NA | NA | NA |
| Bacteria | Firmicutes | Bacilli | Bacillales | Bacillaceae | Anoxybacillus |
| Bacteria | Proteobacteria | Alphaproteobacteria | Rhizobiales | Beijerinckiaceae | Methylobacterium-Methylorubrum |
| Bacteria | Proteobacteria | Gammaproteobacteria | Burkholderiales | Neisseriaceae | Neisseria |
| Bacteria | Firmicutes | Bacilli | Lactobacillales | Streptococcaceae | Lactococcus |
| Bacteria | Firmicutes | Bacilli | Bacillales | Bacillaceae | Bacillus |
| Bacteria | Proteobacteria | Gammaproteobacteria | Pseudomonadales | Pseudomonadaceae | Pseudomonas |
| Bacteria | Actinobacteriota | Actinobacteria | Corynebacteriales | Corynebacteriaceae | Corynebacterium |
| Bacteria | Proteobacteria | Alphaproteobacteria | Caulobacterales | Caulobacteraceae | Brevundimonas |
| Bacteria | Proteobacteria | Gammaproteobacteria | Pseudomonadales | Moraxellaceae | Acinetobacter |
| Bacteria | Firmicutes | Bacilli | Bacillales | Bacillaceae | Bacillus |
| Bacteria | Proteobacteria | Alphaproteobacteria | Caulobacterales | Caulobacteraceae | NA |
| Bacteria | Proteobacteria | Gammaproteobacteria | Enterobacterales | NA | NA |
| Bacteria | Proteobacteria | Gammaproteobacteria | Enterobacterales | Enterobacteriaceae | NA |
| Bacteria | Bacteroidota | Bacteroidia | Chitinophagales | Chitinophagaceae | Sediminibacterium |
| Bacteria | Proteobacteria | Gammaproteobacteria | Burkholderiales | Comamonadaceae | Tepidimonas |
| Bacteria | Proteobacteria | Alphaproteobacteria | Rhizobiales | Hyphomicrobiaceae | Pedomicrobium |
| Bacteria | Myxococcota | Polyangia | Polyangiales | Polyangiaceae | Pajaroellobacter |
| Bacteria | Deinococcota | Deinococci | Thermales | Thermaceae | Thermus |
| Bacteria | Proteobacteria | Gammaproteobacteria | Burkholderiales | Comamonadaceae | Paucibacter |
| Bacteria | Proteobacteria | Gammaproteobacteria | Xanthomonadales | Xanthomonadaceae | Stenotrophomonas |
| Bacteria | Cyanobacteria | Cyanobacteriia | Chloroplast | NA | NA |
| Bacteria | Desulfobacterota | Desulfuromonadia | Geobacterales | Geobacteraceae | Geobacter |
| Bacteria | Proteobacteria | Alphaproteobacteria | Sphingomonadales | Sphingomonadaceae | Sphingomonas |
| Bacteria | Bacteroidota | Bacteroidia | Chitinophagales | Chitinophagaceae | Vibrionimonas |
| Bacteria | Proteobacteria | Gammaproteobacteria | Burkholderiales | Burkholderiaceae | Ralstonia |
| Bacteria | Proteobacteria | Gammaproteobacteria | Pseudomonadales | Moraxellaceae | Enhydrobacter |
| Bacteria | Proteobacteria | Gammaproteobacteria | JG36-TzT-191 | NA | NA |
| Bacteria | Proteobacteria | Gammaproteobacteria | Pseudomonadales | Moraxellaceae | Acinetobacter |
| Bacteria | Proteobacteria | Alphaproteobacteria | Caulobacterales | Caulobacteraceae | Caulobacter |
| Bacteria | Proteobacteria | Alphaproteobacteria | Sphingomonadales | Sphingomonadaceae | Sphingomonas |
| Bacteria | Firmicutes | Bacilli | Lactobacillales | Streptococcaceae | Streptococcus |
| Bacteria | Proteobacteria | Alphaproteobacteria | Rickettsiales | Mitochondria | NA |
| Bacteria | Proteobacteria | Gammaproteobacteria | Burkholderiales | Comamonadaceae | Polaromonas |
| Bacteria | Firmicutes | Bacilli | Lactobacillales | Lactobacillaceae | Lactobacillus |
| Bacteria | Proteobacteria | Alphaproteobacteria | Rhizobiales | Xanthobacteraceae | Bradyrhizobium |
| Bacteria | Proteobacteria | Gammaproteobacteria | Enterobacterales | Enterobacteriaceae | Escherichia-Shigella |
| Bacteria | Actinobacteriota | Actinobacteria | Micrococcales | Micrococcaceae | Rothia |
| Bacteria | Proteobacteria | Gammaproteobacteria | Xanthomonadales | Xanthomonadaceae | Stenotrophomonas |
| Bacteria | Actinobacteriota | Thermoleophilia | Solirubrobacterales | Solirubrobacteraceae | Patulibacter |
| Bacteria | Actinobacteriota | Actinobacteria | Corynebacteriales | Corynebacteriaceae | Lawsonella |
| Bacteria | Proteobacteria | Gammaproteobacteria | Pasteurellales | Pasteurellaceae | Haemophilus |
| Bacteria | Firmicutes | Clostridia | Eubacteriales | Eubacteriaceae | Acetobacterium |
| Bacteria | Proteobacteria | Gammaproteobacteria | Cellvibrionales | Porticoccaceae | C1-B045 |
| Bacteria | Proteobacteria | Gammaproteobacteria | Xanthomonadales | Xanthomonadaceae | Stenotrophomonas |
| Bacteria | Proteobacteria | Gammaproteobacteria | Pseudomonadales | Moraxellaceae | Psychrobacter |
| Bacteria | Bacteroidota | Bacteroidia | Sphingobacteriales | Sphingobacteriaceae | Mucilaginibacter |
| Bacteria | Campilobacterota | Campylobacteria | Campylobacterales | Sulfurimonadaceae | Sulfuricurvum |
| Bacteria | Proteobacteria | Gammaproteobacteria | Xanthomonadales | Xanthomonadaceae | Stenotrophomonas |
| Bacteria | Proteobacteria | Gammaproteobacteria | Burkholderiales | Neisseriaceae | Neisseria |
| Bacteria | Armatimonadota | Armatimonadia | Armatimonadales | NA | NA |
| Bacteria | Actinobacteriota | Actinobacteria | Micrococcales | Micrococcaceae | Micrococcus |
| Bacteria | Proteobacteria | Alphaproteobacteria | Rhizobiales | Beijerinckiaceae | Methylocella |

**Table S2.** Potential contaminant genera removed from analyses using universal primer set identified by taxonomic affiliation based on the Census of Deep Life dataset [95] (Sheik et al., 2018).

| Phylum | Class | Contaminant genera |
| --- | --- | --- |
| Proteobacteria | Gammaproteobacteria | Acinetobacter |
| Actinobacteriota | Actinobacteria | Actinomyces |
| Actinobacteria | Actinobacteria | Aeromicrobium |
| Proteobacteria | Alphaproteobacteria | Afipia |
| Proteobacteria | Gammaproteobacteria | Aquabacterium |
| Actinobacteria | Actinobacteria | Arthrobacter |
| Proteobacteria | Alphaproteobacteria | Asticcacaulis |
| Proteobacteria | Alphaproteobacteria | Aurantimonas |
| Proteobacteria | Gammaproteobacteria | Azoarcus |
| Firmicutes | Bacilli | Bacillus |
| Proteobacteria | Alphaproteobacteria | Bosea |
| Proteobacteria | Alphaproteobacteria | Bradyrhizobium |
| Firmicutes | Bacilli | Brevibacillus |
| Actinobacteria | Actinobacteria | Brevibacterium |
| Proteobacteria | Alphaproteobacteria | Brevundimonas |
| Firmicutes | Bacilli | Brochothrix |
| Proteobacteria | Gammaproteobacteria | Burkholderia-Caballeronia-Paraburkholderia |
| Campilobacterota | Campylobacteria | Campylobacter |
| Proteobacteria | Alphaproteobacteria | Caulobacter |
| Bacteroidetes | Bacteroidia | Chryseobacterium |
| Bacteroidota | Bacteroidia | Cloacibacterium |
| Proteobacteria | Gammaproteobacteria | Comamonas |
| Actinobacteria | Actinobacteria | Corynebacterium |
| Proteobacteria | Gammaproteobacteria | Cupriavidus |
| Actinobacteria | Actinobacteria | Curtobacterium |
| Proteobacteria | Gammaproteobacteria | Curvibacter |
| Actinobacteria | Actinobacteria | Cutibacterium |
| Deinococcus-Thermus | Deinococcus | Deinococcus |
| Proteobacteria | Gammaproteobacteria | Delftia |
| Actinobacteria | Actinobacteria | Dermabacter |
| Proteobacteria | Alphaproteobacteria | Devosia |
| Actinobacteria | Actinobacteria | Dietzia |
| Proteobacteria | Gammaproteobacteria | Duganella |
| Bacteroidetes | Bacteroidia | Dyadobacter |
| Proteobacteria | Gammaproteobacteria | Enhydrobacter |
| Firmicutes | Bacilli | Enterococcus |
| Proteobacteria | Gammaproteobacteria | Escherichia-Shigella |
| Firmicutes | Bacilli | Facklamia |
| Bacteroidetes | Bacteroidia | Flavobacterium |
| Fusobacteria | Fusobacteriia | Fusobacterium |
| Proteobacteria | Gammaproteobacteria | Haemophilus |
| Proteobacteria | Gammaproteobacteria | Hafnia-Obesumbacterium |
| Proteobacteria | Alphaproteobacteria | Hoefleae |
| Bacteroidota | Bacteroidia | Hymenobacter |
| Proteobacteria | Gammaproteobacteria | Janthinobacterium |
| Proteobacteria | Gammaproteobacteria | Kingella |
| Proteobacteria | Gammaproteobacteria | Klebsiella |
| Actinobacteria | Actinobacteria | Kocuria |
| Firmicutes | Bacilli | Lactobacillus |
| Firmicutes | Bacilli | Lactococcus |
| Firmicutes | Bacilli | Leuconostoc |
| Proteobacteria | Gammaproteobacteria | Limnobacter |
| Firmicutes | Bacilli | Macrococcus |
| Proteobacteria | Gammaproteobacteria | Massilia |
| Proteobacteria | Alphaproteobacteria | Mesorhizobium |
| Proteobacteria | Alphaproteobacteria | Methylobacterium |
| Proteobacteria | Alphaproteobacteria | Methylobacterium-Methylorubrum |
| Proteobacteria | Gammaproteobacteria | Methylophilus |
| Proteobacteria | Gammaproteobacteria | Methyloversatilis |
| Actinobacteria | Actinobacteria | Microbacterium |
| Actinobacteria | Actinobacteria | Micrococcus |
| Actinobacteria | Actinobacteria | Microlunatus |
| Proteobacteria | Gammaproteobacteria | Morganella |
| Proteobacteria | Gammaproteobacteria | Neisseria |
| Proteobacteria | Gammaproteobacteria | Nevskia |
| Proteobacteria | Alphaproteobacteria | Novosphingobium |
| Firmicutes | Bacilli | Paenibacillus |
| Proteobacteria | Alphaproteobacteria | Paracoccus |
| Actinobacteria | Thermoleophilia | Patulibacter |
| Bacteroidetes | Bacteroidia | Pedobacter |
| Proteobacteria | Alphaproteobacteria | Pedomicrobium |
| Proteobacteria | Gammaproteobacteria | Pelomonas |
| Proteobacteria | Alphaproteobacteria | Phyllobacterium |
| Proteobacteria | Gammaproteobacteria | Polaromonas |
| Bacteroidetes | Bacteroidia | Prevotella |
| Proteobacteria | Gammaproteobacteria | Proteus |
| Proteobacteria | Gammaproteobacteria | Pseudomonas |
| Proteobacteria | Gammaproteobacteria | Pseudoxanthomonas |
| Proteobacteria | Gammaproteobacteria | Psychrobacter |
| Proteobacteria | Gammaproteobacteria | Ralstonia |
| Proteobacteria | Gammaproteobacteria | Raoultella |
| Actinobacteria | Actinobacteria | Rhodococcus |
| Proteobacteria | Alphaproteobacteria | Roseomonas |
| Actinobacteriota | Actinobacteria | Rothia |
| Proteobacteria | Gammaproteobacteria | Schlegelella |
| Proteobacteria | Gammaproteobacteria | Serratia |
| Proteobacteria | Alphaproteobacteria | Sphingobium |
| Proteobacteria | Alphaproteobacteria | Sphingomonas |
| Firmicutes | Bacilli | Staphylococcus |
| Proteobacteria | Gammaproteobacteria | Stenotrophomonas |
| Firmicutes | Bacilli | Streptococcus |
| Proteobacteria | Gammaproteobacteria | Undibacterium |
| Proteobacteria | Gammaproteobacteria | Variovorax |
| Firmicutes | Negativicutes | Veillonella |
| Proteobacteria | Gammaproteobacteria | Xanthomonas |

**Table S3.** Quality filtration results obtained on paired-end reads and number of sequences per library before and after contaminant removal using universal and archaeal primer sets. n.d., not detected

| Samples names | Bacterial 16S rRNA encoding gene tag sequencing Prokaryotic primer set (515F/806R) | | | | | | | Archaeal 16S rRNA encoding gene tag sequencing Second round nested PCR (Parch340F/Arch958VR) | | | | |
| --- | --- | --- | --- | --- | --- | --- | --- | --- | --- | --- | --- | --- |
|  | Paired-end reads | High quality paired-end reads | Merged paired-end reads | High quality merged paired-end reads | Total Reads Per Library after contaminant removal | % of reads kept after contaminant removal | Total Reads Per Library after simple overlap approach (203+128+16+14 ASVs removed) | Paired-end reads | High quality paired-end reads | Merged paired-end reads | High quality merged paired-end reads | Total Reads Per Library after contaminant removal |
| Sur161/Sur1a | 21631 | 17085 | 15002 | 14221 | 10031 | 70.54 | 10031 | 114382 | 62942 | 18858 | 7901 | 7796 |
| Sur162/Sur2a | 21393 | 15859 | 14919 | 13414 | 7334 | 54.67 | 7334 | 95411 | 46267 | 11205 | 7565 | 3354 |
| Sur163 | 25688 | 19760 | 17950 | 14980 | 4779 | 31.90 | 4779 | n.d. | n.d. | n.d. | n.d. | n.d. |
| Sur164/Sur4a | 30114 | 22488 | 21424 | 19714 | 2231 | 11.32 | 2231 | 67387 | 51387 | 4619 | 510 | 443 |
| Sur165 | 22659 | 15444 | 14300 | 13209 | 2760 | 20.89 | 2760 | n.d. | n.d. | n.d. | n.d. | n.d. |
| Sur166/Sur6a | 19351 | 14414 | 12152 | 11157 | 2078 | 18.63 | 2078 | 81432 | 34955 | 8417 | 1067 | 104 |
| Sur167/Sur7a | 36566 | 32137 | 27847 | 20780 | 10085 | 48.53 | 10085 | 118294 | 52571 | 19707 | 2251 | 420 |
| Sur168 | 19767 | 13662 | 12166 | 10028 | 6831 | 68.12 | 6831 | n.d. | n.d. | n.d. | n.d. | n.d. |
| Sur169 | 38089 | 24743 | 23535 | 22249 | 2082 | 9.36 | 2082 | n.d. | n.d. | n.d. | n.d. | n.d. |
| Sur1610/Sur10a | 41805 | 26159 | 24205 | 22764 | 11850 | 52.06 | 11850 | 66947 | 52799 | 1737 | 1392 | 1265 |
| Sur16Mix/SurMixa | 33455 | 23270 | 21369 | 20458 | 14612 | 71.42 | 14612 | 75781 | 62652 | 2103 | 1390 | 1080 |
| Sur171 | 38372 | 31761 | 27411 | 24072 | 20592 | 85.54 | 20592 | n.d. | n.d. | n.d. | n.d. | n.d. |
| Sur172 | 32615 | 21133 | 19288 | 15772 | 4410 | 27.96 | 4410 | n.d. | n.d. | n.d. | n.d. | n.d. |
| Sur173 | 30901 | 19845 | 18390 | 15271 | 3822 | 25.03 | 3822 | n.d. | n.d. | n.d. | n.d. | n.d. |
| Sur174 | 35403 | 22030 | 19437 | 17208 | 5683 | 33.03 | 5683 | n.d. | n.d. | n.d. | n.d. | n.d. |
| Sur175 | 39480 | 18477 | 17198 | 14431 | 4391 | 30.43 | 4391 | n.d. | n.d. | n.d. | n.d. | n.d. |
| Sur176 | 37538 | 28955 | 26977 | 25728 | 17261 | 67.09 | 17261 | n.d. | n.d. | n.d. | n.d. | n.d. |
| Sur177 | 23844 | 18532 | 16980 | 15905 | 11124 | 69.94 | 11124 | n.d. | n.d. | n.d. | n.d. | n.d. |
| C4b | 149656 | 84792 | 57363 | 40929 | 25003 | 61.09 | 9940 | n.d. | n.d. | n.d. | n.d. | n.d. |
| C9b | 114949 | 75183 | 56362 | 37412 | 28629 | 76.52 | 24322 | n.d. | n.d. | n.d. | n.d. | n.d. |
| C13b/C13a | 129735 | 89304 | 61233 | 40664 | 11568 | 28.45 | 5996 | 168555 | 165030 | 105492 | 66668 | 64659 |
| C17b | 159730 | 82983 | 64113 | 49818 | 21877 | 43.91 | 8516 | n.d. | n.d. | n.d. | n.d. | n.d. |
| C22b/C22a | 283587 | 210686 | 148894 | 50956 | 28813 | 56.54 | 9386 | 245823 | 238266 | 134452 | 70119 | 57342 |
| C27b/C27a | 63290 | 48329 | 36064 | 28444 | 8390 | 29.50 | 3509 | 247246 | 242498 | 172822 | 111418 | 104662 |
| C33b | 337419 | 132411 | 111671 | 77798 | 13228 | 17.00 | 13084 | n.d. | n.d. | n.d. | n.d. | n.d. |
| C36b/C36a | 99015 | 55755 | 39835 | 30332 | 15525 | 51.18 | 4645 | 190782 | 186639 | 119813 | 94530 | 70944 |
| C39b | 111360 | 61910 | 45179 | 38208 | 15476 | 40.50 | 7348 | n.d. | n.d. | n.d. | n.d. | n.d. |
| C42b/C42a | 94538 | 49486 | 36940 | 31119 | 14241 | 45.76 | 6903 | 225883 | 222331 | 169459 | 104806 | 100922 |
| C45b/C45a | 113506 | 63278 | 43725 | 35998 | 16428 | 45.64 | 4849 | 160039 | 155988 | 94250 | 65761 | 65732 |
| C49b/C49a | 128346 | 76497 | 56166 | 38297 | 17116 | 44.69 | 12662 | 214906 | 210783 | 143938 | 100753 | 90559 |
| C52b/C52a | 128314 | 66994 | 44278 | 33464 | 18879 | 56.42 | 4725 | 153922 | 151905 | 120432 | 70990 | 69414 |
| C55b/C55a | 107505 | 52731 | 41709 | 31878 | 6425 | 20.15 | 5789 | 195721 | 190273 | 118658 | 111775 | 111742 |
| C59b | 124719 | 72974 | 55567 | 43280 | 16214 | 37.46 | 13321 | n.d. | n.d. | n.d. | n.d. | n.d. |
| C62b | 122583 | 52047 | 38703 | 29108 | 8555 | 29.39 | 3586 | n.d. | n.d. | n.d. | n.d. | n.d. |
| C65b/C65a | 130328 | 70225 | 48424 | 35497 | 17436 | 49.12 | 6309 | 206634 | 203494 | 154504 | 103472 | 97615 |
| Fum171 | 28388 | 23961 | 18546 | 17541 | 10269 | 58.54 | 10269 | n.d. | n.d. | n.d. | n.d. | n.d. |
| Fum172 | 24748 | 19757 | 14612 | 14247 | 6758 | 47.43 | 6758 | n.d. | n.d. | n.d. | n.d. | n.d. |
| 1B0ZC | 338 | 302 | 235 | 235 | 235 | 100.00 | 235 | n.d. | n.d. | n.d. | n.d. | n.d. |
| 1B3ZC | 420 | 390 | 192 | 192 | 135 | 70.31 | 135 | n.d. | n.d. | n.d. | n.d. | n.d. |
| 1B25ZC | 257 | 239 | 166 | 166 | 160 | 96.39 | 160 | n.d. | n.d. | n.d. | n.d. | n.d. |
| 149ZC | 20681 | 19936 | 15105 | 13518 | 11945 | 88.36 | 11945 | n.d. | n.d. | n.d. | n.d. | n.d. |
| 1C17ZC | 23095 | 22089 | 16899 | 15941 | 14129 | 88.63 | 14129 | n.d. | n.d. | n.d. | n.d. | n.d. |
| 1C39ZC | 30785 | 29563 | 23845 | 21030 | 19887 | 94.56 | 19887 | n.d. | n.d. | n.d. | n.d. | n.d. |
| 1C51ZC | 30704 | 29714 | 22904 | 19847 | 19012 | 95.79 | 19012 | n.d. | n.d. | n.d. | n.d. | n.d. |
| 1C59ZC | 30326 | 29111 | 21041 | 18301 | 17691 | 96.67 | 17691 | n.d. | n.d. | n.d. | n.d. | n.d. |
| SW_10 | 97945 | 75539 | 53295 | 36219 | 32384 | 89.41 | 32384 | n.d. | n.d. | n.d. | n.d. | n.d. |
| SW_20 | 100020 | 76789 | 57226 | 41002 | 38218 | 93.21 | 38218 | n.d. | n.d. | n.d. | n.d. | n.d. |
| SW_30 | 92617 | 71046 | 51516 | 39387 | 37973 | 96.41 | 37973 | n.d. | n.d. | n.d. | n.d. | n.d. |
| SW_50 | 104804 | 81269 | 59967 | 43973 | 43097 | 98.01 | 43097 | n.d. | n.d. | n.d. | n.d. | n.d. |
| Crb | 124802 | 28915 | 22072 | 16378 | 0 | 0.00 | 0 | n.d. | n.d. | n.d. | n.d. | n.d. |
| Cwb | 121130 | 36145 | 30836 | 27195 | 0 | 0.00 | 0 | n.d. | n.d. | n.d. | n.d. | n.d. |
| **TOTAL** |  |  |  |  |  |  | **455545** |  |  |  |  | **848053** |

**Table S4.** Contaminant ASVs removed from analyses using archaeal primer set [26] that were identified using the extraction blank. NA., Not assigned.

| Kingdom | Phylum | Class | Order | Family | Genus |
| --- | --- | --- | --- | --- | --- |
| Archaea | Crenarchaeota | Nitrososphaeria | Caldiarchaeales | Geothermarchaeaceae | NA |
| Archaea | Hydrothermarchaeota | Hydrothermarchaeia | Hydrothermarchaeales | NA | NA |
| Archaea | Crenarchaeota | Thermoprotei | Desulfurococcales | NA | NA |
| Archaea | Crenarchaeota | Thermoprotei | Desulfurococcales | NA | NA |
| Archaea | Crenarchaeota | Thermoprotei | Desulfurococcales | NA | NA |
| Archaea | Hydrothermarchaeota | Hydrothermarchaeia | Hydrothermarchaeales | NA | NA |
| Archaea | Crenarchaeota | Nitrososphaeria | Caldiarchaeales | Geothermarchaeaceae | NA |
| Archaea | Crenarchaeota | Nitrososphaeria | Caldiarchaeales | Geothermarchaeaceae | NA |
| Archaea | Crenarchaeota | Nitrososphaeria | Caldiarchaeales | Geothermarchaeaceae | NA |
| Archaea | Hydrothermarchaeota | Hydrothermarchaeia | Hydrothermarchaeales | NA | NA |
| Archaea | Hydrothermarchaeota | Hydrothermarchaeia | Hydrothermarchaeales | NA | NA |
| Archaea | Crenarchaeota | Nitrososphaeria | Caldiarchaeales | Geothermarchaeaceae | NA |
| Archaea | Crenarchaeota | Thermoprotei | Desulfurococcales | NA | NA |
| Archaea | Crenarchaeota | Thermoprotei | Thermoproteales | Thermofilaceae | Thermofilum |
| Archaea | Crenarchaeota | Thermoprotei | Thermoproteales | Thermofilaceae | Thermofilum |
| Archaea | NA | NA | NA | NA | NA |
| Archaea | Crenarchaeota | NA | NA | NA | NA |
| Archaea | Crenarchaeota | Thermoprotei | Desulfurococcales | NA | NA |
| Archaea | Crenarchaeota | Thermoprotei | Desulfurococcales | NA | NA |
| Archaea | NA | NA | NA | NA | NA |
| Archaea | NA | NA | NA | NA | NA |
| Archaea | Crenarchaeota | Thermoprotei | Desulfurococcales | NA | NA |
| Archaea | Crenarchaeota | Thermoprotei | Desulfurococcales | NA | NA |
| Archaea | Crenarchaeota | Thermoprotei | Desulfurococcales | NA | NA |
| Archaea | Crenarchaeota | Thermoprotei | Desulfurococcales | NA | NA |
| Archaea | Crenarchaeota | Thermoprotei | Desulfurococcales | NA | NA |

**Table S5.** Significant ASVs identified with DESeq2 [95 unique ASVs using the universal primer set; 26 unique ASVs using the archaeal primer sets].

| baseMean | log2FoldChange | lfcSE | stat | pvalue | padj | Kingdom | Phylum | Class | Order | Family | Genus |
| --- | --- | --- | --- | --- | --- | --- | --- | --- | --- | --- | --- |
| **Universal primer set:** | | | | | | | | | | | |
| **Borehole fluid vs Drill core [70 ASVs]** | | | | | | | | | | | |
| 21.87 | -23.97 | 3.03 | -7.91 | 2.48E-15 | 1.72E-14 | Bacteria | Proteobacteria | Gammaproteobacteria | Oceanospirillales | Saccharospirillaceae | Oleispira |
| 20.89 | -23.88 | 3.03 | -7.89 | 3.03E-15 | 2.03E-14 | Bacteria | Proteobacteria | Gammaproteobacteria | Alteromonadales | Alteromonadaceae | Glaciecola |
| 14.57 | -23.82 | 3.00 | -7.94 | 2.03E-15 | 1.51E-14 | Bacteria | Proteobacteria | Gammaproteobacteria | Oceanospirillales | Saccharospirillaceae | Oceaniserpentilla |
| 34.71 | -23.48 | 2.21 | -10.63 | 2.14E-26 | 1.62E-24 | Bacteria | Cyanobacteria | Cyanobacteriia | Synechococcales | Cyanobiaceae | Synechococcus CC9902 |
| 14.36 | -23.42 | 3.16 | -7.41 | 1.29E-13 | 6.69E-13 | Bacteria | Proteobacteria | Gammaproteobacteria | Alteromonadales | Psychromonadaceae | Psychromonas |
| 14.50 | -23.40 | 3.16 | -7.40 | 1.34E-13 | 6.79E-13 | Bacteria | Firmicutes | Bacilli | Bacillales | Bacillaceae | Geobacillus |
| 12.42 | -23.22 | 3.16 | -7.34 | 2.08E-13 | 9.88E-13 | Bacteria | Proteobacteria | Gammaproteobacteria | Burkholderiales | Comamonadaceae | Piscinibacter |
| 20.41 | -22.75 | 2.33 | -9.76 | 1.63E-22 | 5.31E-21 | Bacteria | Bacteroidota | Bacteroidia | Flavobacteriales | Flavobacteriaceae | NA |
| 20.78 | -22.72 | 2.69 | -8.45 | 3.04E-17 | 2.89E-16 | Bacteria | Proteobacteria | Alphaproteobacteria | Rhodobacterales | Rhodobacteraceae | Planktomarina |
| 17.57 | -22.61 | 2.33 | -9.71 | 2.79E-22 | 7.96E-21 | Bacteria | Bacteroidota | Bacteroidia | Flavobacteriales | Flavobacteriaceae | NS4 marine group |
| 487.76 | -12.58 | 1.28 | -9.80 | 1.12E-22 | 4.27E-21 | Bacteria | Proteobacteria | Alphaproteobacteria | Sphingomonadales | Sphingomonadaceae | NA |
| 366.90 | -12.17 | 1.28 | -9.50 | 2.04E-21 | 5.16E-20 | Bacteria | Proteobacteria | Alphaproteobacteria | Sphingomonadales | Sphingomonadaceae | NA |
| 66.97 | -9.72 | 1.79 | -5.43 | 5.61E-08 | 2.28E-07 | Bacteria | Actinobacteriota | Actinobacteria | Micrococcales | Microbacteriaceae | Galbitalea |
| 47.25 | -9.21 | 2.10 | -4.39 | 1.13E-05 | 4.50E-05 | Bacteria | Proteobacteria | Gammaproteobacteria | Alteromonadales | Alteromonadaceae | Glaciecola |
| 47.03 | -9.19 | 2.34 | -3.94 | 8.24E-05 | 0.000303115 | Bacteria | Actinobacteriota | Actinobacteria | Micrococcales | Micrococcaceae | Pseudarthrobacter |
| 35.45 | -8.80 | 2.41 | -3.65 | 0.000266169 | 0.000936377 | Bacteria | Actinobacteriota | Actinobacteria | Micrococcales | Microbacteriaceae | Galbitalea |
| 29.62 | -8.54 | 2.07 | -4.12 | 3.81E-05 | 0.00014464 | Bacteria | Proteobacteria | Gammaproteobacteria | Burkholderiales | Comamonadaceae | Xylophilus |
| 27.29 | -8.42 | 1.93 | -4.37 | 1.23E-05 | 4.84E-05 | Bacteria | Proteobacteria | Alphaproteobacteria | Rhizobiales | Rhizobiales Incertae Sedis | Phreatobacter |
| 17.13 | -7.59 | 2.51 | -3.02 | 0.002504267 | 0.008156756 | Bacteria | Bacteroidota | Bacteroidia | Flavobacteriales | Flavobacteriaceae | Flavicella |
| 34.12 | -7.53 | 1.78 | -4.22 | 2.40E-05 | 9.26E-05 | Bacteria | Proteobacteria | Alphaproteobacteria | Reyranellales | Reyranellaceae | Reyranella |
| 12.74 | -7.32 | 2.36 | -3.11 | 0.001892401 | 0.006345108 | Bacteria | Bacteroidota | Bacteroidia | Flavobacteriales | Flavobacteriaceae | Polaribacter |
| 21.13 | 7.66 | 2.45 | 3.12 | 0.001803238 | 0.006136392 | Bacteria | Actinobacteriota | Thermoleophilia | Solirubrobacterales | Solirubrobacteraceae | NA |
| 92.04 | 8.01 | 2.65 | 3.03 | 0.00247062 | 0.008156756 | Archaea | Thermoplasmatota | Thermoplasmata | NA | NA | NA |
| 38.96 | 8.54 | 2.34 | 3.65 | 0.00026695 | 0.000936377 | Bacteria | Actinobacteriota | Thermoleophilia | Solirubrobacterales | Solirubrobacteraceae | Conexibacter |
| 143.87 | 8.56 | 2.51 | 3.41 | 0.000647127 | 0.00223553 | Archaea | Thermoplasmatota | Thermoplasmata | NA | NA | NA |
| 45.42 | 8.76 | 2.21 | 3.97 | 7.22E-05 | 0.000269725 | Bacteria | Proteobacteria | Alphaproteobacteria | Rhodobacterales | Rhodobacteraceae | Rhodobaca |
| 47.19 | 8.82 | 2.32 | 3.79 | 0.000147663 | 0.000534399 | Bacteria | Actinobacteriota | Actinobacteria | Pseudonocardiales | Pseudonocardiaceae | Pseudonocardia |
| 6.17 | 21.85 | 3.16 | 6.91 | 4.98E-12 | 2.07E-11 | Bacteria | Proteobacteria | Alphaproteobacteria | Rhodobacterales | Rhodobacteraceae | Ruegeria |
| 7.73 | 22.26 | 3.16 | 7.04 | 1.97E-12 | 8.33E-12 | Archaea | Euryarchaeota | Thermococci | Thermococcales | Thermococcaceae | Thermococcus |
| 9.61 | 22.61 | 3.16 | 7.15 | 8.97E-13 | 3.86E-12 | Bacteria | Proteobacteria | Gammaproteobacteria | Oceanospirillales | Halomonadaceae | Halomonas |
| 11.48 | 22.69 | 3.16 | 7.17 | 7.36E-13 | 3.23E-12 | Bacteria | Proteobacteria | Alphaproteobacteria | Rhodobacterales | Rhodobacteraceae | Ruegeria |
| 7.86 | 22.85 | 3.16 | 7.22 | 5.19E-13 | 2.32E-12 | Bacteria | Actinobacteriota | Actinobacteria | Corynebacteriales | Mycobacteriaceae | Mycobacterium |
| 7.36 | 23.07 | 3.16 | 7.29 | 3.05E-13 | 1.39E-12 | Bacteria | Proteobacteria | Alphaproteobacteria | Rhizobiales | Rhizobiaceae | Chelativorans |
| 8.70 | 23.17 | 3.02 | 7.66 | 1.85E-14 | 1.07E-13 | Bacteria | Proteobacteria | Alphaproteobacteria | Rhizobiales | Beijerinckiaceae | Salinarimonas |
| 9.04 | 23.21 | 3.16 | 7.34 | 2.19E-13 | 1.02E-12 | Bacteria | Actinobacteriota | Acidimicrobiia | Microtrichales | Iamiaceae | Iamia |
| 11.58 | 23.33 | 3.16 | 7.37 | 1.67E-13 | 8.10E-13 | Bacteria | Firmicutes | Desulfitobacteriia | Desulfitobacteriales | Desulfitobacteriaceae | Desulfosporosinus |
| 15.39 | 23.34 | 3.16 | 7.38 | 1.62E-13 | 8.04E-13 | Bacteria | Actinobacteriota | RBG-16-55-12 | NA | NA | NA |
| 78.81 | 23.45 | 2.93 | 8.00 | 1.20E-15 | 1.01E-14 | Bacteria | Firmicutes | Desulfotomaculia | Ammonifexales | NA | NA |
| 12.76 | 23.51 | 3.04 | 7.74 | 9.87E-15 | 5.92E-14 | Bacteria | Proteobacteria | Gammaproteobacteria | Alteromonadales | Shewanellaceae | Shewanella |
| 16.26 | 23.52 | 3.16 | 7.43 | 1.06E-13 | 5.63E-13 | Bacteria | Desulfobacterota | Desulfobacteria | Desulfatiglandales | Desulfatiglandaceae | Desulfatiglans |
| 12.59 | 23.58 | 3.04 | 7.75 | 9.20E-15 | 5.67E-14 | Bacteria | Actinobacteriota | Actinobacteria | Micrococcales | Microbacteriaceae | Microcella |
| 13.74 | 23.75 | 3.16 | 7.51 | 6.02E-14 | 3.27E-13 | Bacteria | Actinobacteriota | Thermoleophilia | Solirubrobacterales | Solirubrobacteraceae | Conexibacter |
| 9.00 | 23.96 | 3.16 | 7.57 | 3.65E-14 | 2.03E-13 | Bacteria | Firmicutes | Desulfotomaculia | Desulfotomaculales | Desulfallas-Sporotomaculum | BRH-c8a |
| 17.55 | 23.97 | 3.07 | 7.82 | 5.34E-15 | 3.38E-14 | Bacteria | Acetothermia | Acetothermiia | NA | NA | NA |
| 23.86 | 24.19 | 3.08 | 7.86 | 3.87E-15 | 2.52E-14 | Archaea | Hydrothermarchaeota | Hydrothermarchaeia | Hydrothermarchaeales | NA | NA |
| 20.77 | 24.23 | 3.16 | 7.66 | 1.87E-14 | 1.07E-13 | Bacteria | Actinobacteriota | RBG-16-55-12 | NA | NA | NA |
| 25.20 | 24.40 | 3.08 | 7.93 | 2.25E-15 | 1.61E-14 | Bacteria | Actinobacteriota | RBG-16-55-12 | NA | NA | NA |
| 154.21 | 24.42 | 2.61 | 9.37 | 7.29E-21 | 1.66E-19 | Bacteria | Acetothermia | Acetothermiia | NA | NA | NA |
| 28.80 | 24.54 | 2.86 | 8.57 | 9.97E-18 | 1.04E-16 | Bacteria | Actinobacteriota | Actinobacteria | Pseudonocardiales | Pseudonocardiaceae | Pseudonocardia |
| 19.48 | 24.72 | 2.84 | 8.71 | 3.12E-18 | 4.18E-17 | Archaea | Halobacterota | Methanosarcinia | Methanosarciniales | Syntrophoarchaeaceae | NA |
| 34.31 | 24.75 | 3.10 | 7.97 | 1.55E-15 | 1.26E-14 | Archaea | Hydrothermarchaeota | Hydrothermarchaeia | Hydrothermarchaeales | NA | NA |
| 50.20 | 24.84 | 3.12 | 7.96 | 1.76E-15 | 1.39E-14 | Bacteria | Firmicutes | Desulfotomaculia | Ammonifexales | Ammonificaceae | NA |
| 40.67 | 24.84 | 2.92 | 8.51 | 1.71E-17 | 1.69E-16 | Bacteria | Patescibacteria | Saccharimonadia | Saccharimonadales | NA | NA |
| 26.10 | 24.86 | 2.85 | 8.72 | 2.86E-18 | 4.08E-17 | Bacteria | Actinobacteriota | Thermoleophilia | Solirubrobacterales | Solirubrobacteraceae | NA |
| 37.08 | 24.95 | 2.97 | 8.41 | 4.27E-17 | 3.89E-16 | Bacteria | Proteobacteria | Gammaproteobacteria | Vibrionales | Vibrionaceae | Aliivibrio |
| 31.17 | 24.99 | 2.88 | 8.68 | 4.13E-18 | 4.71E-17 | Bacteria | Proteobacteria | Alphaproteobacteria | Rhodobacterales | Rhodobacteraceae | Rhodobaca |
| 33.19 | 25.10 | 2.69 | 9.32 | 1.19E-20 | 2.46E-19 | Bacteria | Actinobacteriota | RBG-16-55-12 | NA | NA | NA |
| 32.54 | 25.11 | 3.16 | 7.94 | 2.05E-15 | 1.51E-14 | Bacteria | Firmicutes | Desulfotomaculia | Desulfotomaculales | Desulfallas-Sporotomaculum | BRH-c8a |
| 35.72 | 25.14 | 2.87 | 8.75 | 2.16E-18 | 3.29E-17 | Bacteria | Actinobacteriota | RBG-16-55-12 | NA | NA | NA |
| 26.83 | 25.17 | 3.09 | 8.15 | 3.68E-16 | 3.23E-15 | Bacteria | Acetothermia | Acetothermiia | NA | NA | NA |
| 62.63 | 25.19 | 2.94 | 8.57 | 1.01E-17 | 1.04E-16 | Bacteria | Patescibacteria | Saccharimonadia | Saccharimonadales | NA | NA |
| 48.15 | 25.39 | 2.93 | 8.68 | 4.02E-18 | 4.71E-17 | Bacteria | Firmicutes | Desulfotomaculia | Desulfotomaculales | Desulfallas-Sporotomaculum | BRH-c8a |
| 54.44 | 25.55 | 2.79 | 9.17 | 4.62E-20 | 8.09E-19 | Bacteria | Proteobacteria | Gammaproteobacteria | Vibrionales | Vibrionaceae | Aliivibrio |
| 107.81 | 26.47 | 2.58 | 10.25 | 1.17E-24 | 6.66E-23 | Bacteria | Acetothermia | Acetothermiia | NA | NA | NA |
| 23.39 | 27.24 | 3.09 | 8.82 | 1.19E-18 | 1.93E-17 | Bacteria | Actinobacteriota | Actinobacteria | Streptomycetales | Streptomycetaceae | Streptomyces |
| 25.95 | 27.45 | 3.16 | 8.68 | 4.10E-18 | 4.71E-17 | Archaea | Halobacterota | Methanosarcinia | Methanosarciniales | Syntrophoarchaeaceae | NA |
| 16.80 | 28.06 | 2.84 | 9.87 | 5.87E-23 | 2.68E-21 | Bacteria | Proteobacteria | Alphaproteobacteria | Rhizobiales | Rhizobiaceae | Chelativorans |
| 11.30 | 29.31 | 3.16 | 9.26 | 2.00E-20 | 3.79E-19 | Bacteria | Firmicutes | Desulfotomaculia | Desulfotomaculales | Desulfallas-Sporotomaculum | BRH-c8a |
| 11.95 | 29.99 | 2.69 | 11.14 | 7.98E-29 | 9.10E-27 | Bacteria | Proteobacteria | Gammaproteobacteria | Alteromonadales | Pseudoalteromonadaceae | Pseudoalteromonas |
| 20.95 | 30.00 | 2.65 | 11.33 | 9.07E-30 | 2.07E-27 | Bacteria | Firmicutes | Bacilli | NA | NA | NA |
| **Borehole fluid vs Fumarole [16 ASVs]** | | | | | | | | | | | |
| 154.21 | 30.00 | 5.82 | 5.15 | 2.55102E-07 | 7.27041E-05 | Bacteria | Acetothermia | Acetothermiia | NA | NA | NA |
| 143.87 | 24.36 | 5.64 | 4.32 | 1.54007E-05 | 0.002194606 | Archaea | Thermoplasmatota | Thermoplasmata | NA | NA | NA |
| 107.81 | 24.11 | 5.77 | 4.18 | 2.91873E-05 | 0.002772796 | Bacteria | Acetothermia | Acetothermiia | NA | NA | NA |
| 33.19 | 22.41 | 6.01 | 3.73 | 0.000193313 | 0.009842092 | Bacteria | Actinobacteriota | RBG-16-55-12 | NA | NA | NA |
| 7.78 | -25.66 | 6.90 | -3.72 | 0.00020096 | 0.009842092 | Bacteria | Bacteroidota | Bacteroidia | Cytophagales | Spirosomaceae | Spirosoma |
| 54.44 | 23.04 | 6.21 | 3.71 | 0.000207202 | 0.009842092 | Bacteria | Proteobacteria | Gammaproteobacteria | Vibrionales | Vibrionaceae | Aliivibrio |
| 26.10 | 22.05 | 6.36 | 3.47 | 0.000521095 | 0.009933149 | Bacteria | Actinobacteriota | Thermoleophilia | Solirubrobacterales | Solirubrobacteraceae | NA |
| 92.04 | 21.27 | 5.93 | 3.59 | 0.000333318 | 0.009933149 | Archaea | Thermoplasmatota | Thermoplasmata | NA | NA | NA |
| 48.15 | 22.94 | 6.52 | 3.52 | 0.000431405 | 0.009933149 | Bacteria | Firmicutes | Desulfotomaculia | Desulfotomaculales | Desulfallas-Sporotomaculum | BRH-c8a |
| 31.17 | 22.33 | 6.42 | 3.48 | 0.000501017 | 0.009933149 | Bacteria | Proteobacteria | Alphaproteobacteria | Rhodobacterales | Rhodobacteraceae | Rhodobaca |
| 20.95 | 20.75 | 5.91 | 3.51 | 0.00044445 | 0.009933149 | Bacteria | Firmicutes | Bacilli | NA | NA | NA |
| 28.80 | 22.06 | 6.38 | 3.46 | 0.000540435 | 0.009933149 | Bacteria | Actinobacteriota | Actinobacteria | Pseudonocardiales | Pseudonocardiaceae | Pseudonocardia |
| 78.81 | 23.48 | 6.52 | 3.60 | 0.000318751 | 0.009933149 | Bacteria | Firmicutes | Desulfotomaculia | Ammonifexales | NA | NA |
| 35.72 | 22.49 | 6.40 | 3.51 | 0.000443077 | 0.009933149 | Bacteria | Actinobacteriota | RBG-16-55-12 | NA | NA | NA |
| 16.80 | 21.88 | 6.34 | 3.45 | 0.00055765 | 0.009933149 | Bacteria | Proteobacteria | Alphaproteobacteria | Rhizobiales | Rhizobiaceae | Chelativorans |
| 62.63 | 23.69 | 6.54 | 3.62 | 0.000294835 | 0.009933149 | Bacteria | Patescibacteria | Saccharimonadia | Saccharimonadales | NA | NA |
| **Borehole fluid vs Seawater samples [53 ASVs]** | | | | | | | | | | | |
| 143.87 | 29.96 | 4.17 | 7.19 | 6.46E-13 | 1.89E-09 | Archaea | Thermoplasmatota | Thermoplasmata | NA | NA | NA |
| 92.04 | 30.00 | 4.39 | 6.84 | 7.88E-12 | 9.72E-09 | Archaea | Thermoplasmatota | Thermoplasmata | NA | NA | NA |
| 34.71 | -24.20 | 3.56 | -6.81 | 9.94E-12 | 9.72E-09 | Bacteria | Cyanobacteria | Cyanobacteriia | Synechococcales | Cyanobiaceae | Synechococcus CC9902 |
| 107.81 | 26.39 | 4.27 | 6.19 | 6.15E-10 | 3.61E-07 | Bacteria | Acetothermia | Acetothermiia | NA | NA | NA |
| 78.81 | 30.00 | 4.83 | 6.21 | 5.16E-10 | 3.61E-07 | Bacteria | Firmicutes | Desulfotomaculia | Ammonifexales | NA | NA |
| 20.41 | -23.12 | 3.76 | -6.16 | 7.45E-10 | 3.64E-07 | Bacteria | Bacteroidota | Bacteroidia | Flavobacteriales | Flavobacteriaceae | NA |
| 17.57 | -22.92 | 3.75 | -6.10 | 1.03E-09 | 4.32E-07 | Bacteria | Bacteroidota | Bacteroidia | Flavobacteriales | Flavobacteriaceae | NS4 marine group |
| 9.10 | 29.75 | 5.03 | 5.91 | 3.42E-09 | 1.25E-06 | Bacteria | Campilobacterota | Campylobacteria | Campylobacterales | Sulfurospirillaceae | Sulfurospirillum |
| 154.21 | 24.82 | 4.30 | 5.77 | 8.12E-09 | 2.46E-06 | Bacteria | Acetothermia | Acetothermiia | NA | NA | NA |
| 7.86 | 30.00 | 5.21 | 5.76 | 8.38E-09 | 2.46E-06 | Bacteria | Actinobacteriota | Actinobacteria | Corynebacteriales | Mycobacteriaceae | Mycobacterium |
| 40.67 | 26.86 | 4.81 | 5.58 | 2.36E-08 | 6.28E-06 | Bacteria | Patescibacteria | Saccharimonadia | Saccharimonadales | NA | NA |
| 9.24 | 28.86 | 5.21 | 5.54 | 2.99E-08 | 6.76E-06 | Bacteria | Campilobacterota | Campylobacteria | Campylobacterales | Sulfurospirillaceae | Sulfurospirillum |
| 33.19 | 24.67 | 4.45 | 5.55 | 2.90E-08 | 6.76E-06 | Bacteria | Actinobacteriota | RBG-16-55-12 | NA | NA | NA |
| 20.78 | -23.57 | 4.35 | -5.42 | 6.05E-08 | 1.27E-05 | Bacteria | Proteobacteria | Alphaproteobacteria | Rhodobacterales | Rhodobacteraceae | Planktomarina |
| 35.72 | 24.92 | 4.74 | 5.26 | 1.43E-07 | 2.80E-05 | Bacteria | Actinobacteriota | RBG-16-55-12 | NA | NA | NA |
| 26.10 | 24.62 | 4.70 | 5.24 | 1.63E-07 | 2.82E-05 | Bacteria | Actinobacteriota | Thermoleophilia | Solirubrobacterales | Solirubrobacteraceae | NA |
| 62.63 | 25.41 | 4.84 | 5.25 | 1.54E-07 | 2.82E-05 | Bacteria | Patescibacteria | Saccharimonadia | Saccharimonadales | NA | NA |
| 48.15 | 25.20 | 4.82 | 5.23 | 1.74E-07 | 2.83E-05 | Bacteria | Firmicutes | Desulfotomaculia | Desulfotomaculales | Desulfallas-Sporotomaculum | BRH-c8a |
| 31.17 | 24.73 | 4.75 | 5.21 | 1.91E-07 | 2.96E-05 | Bacteria | Proteobacteria | Alphaproteobacteria | Rhodobacterales | Rhodobacteraceae | Rhodobaca |
| 28.80 | 24.49 | 4.72 | 5.19 | 2.11E-07 | 3.10E-05 | Bacteria | Actinobacteriota | Actinobacteria | Pseudonocardiales | Pseudonocardiaceae | Pseudonocardia |
| 16.80 | 24.12 | 4.69 | 5.14 | 2.70E-07 | 3.77E-05 | Bacteria | Proteobacteria | Alphaproteobacteria | Rhizobiales | Rhizobiaceae | Chelativorans |
| 19.48 | 23.80 | 4.68 | 5.09 | 3.67E-07 | 4.90E-05 | Archaea | Halobacterota | Methanosarcinia | Methanosarciniales | Syntrophoarchaeaceae | NA |
| 32.12 | 23.26 | 4.61 | 5.05 | 4.43E-07 | 5.66E-05 | Bacteria | Actinobacteriota | Actinobacteria | Micrococcales | Micrococcaceae | Pseudarthrobacter |
| 20.95 | 21.84 | 4.37 | 5.00 | 5.81E-07 | 7.10E-05 | Bacteria | Firmicutes | Bacilli | NA | NA | NA |
| 50.20 | 25.56 | 5.14 | 4.97 | 6.59E-07 | 7.73E-05 | Bacteria | Firmicutes | Desulfotomaculia | Ammonifexales | Ammonificaceae | NA |
| 23.86 | 25.12 | 5.07 | 4.96 | 7.17E-07 | 8.09E-05 | Archaea | Hydrothermarchaeota | Hydrothermarchaeia | Hydrothermarchaeales | NA | NA |
| 25.20 | 25.02 | 5.07 | 4.94 | 7.98E-07 | 8.67E-05 | Bacteria | Actinobacteriota | RBG-16-55-12 | NA | NA | NA |
| 15.69 | 24.25 | 4.96 | 4.89 | 1.02E-06 | 0.00010721 | Bacteria | Proteobacteria | Alphaproteobacteria | Reyranellales | Reyranellaceae | Reyranella |
| 23.39 | 24.83 | 5.09 | 4.88 | 1.06E-06 | 0.00010754 | Bacteria | Actinobacteriota | Actinobacteria | Streptomycetales | Streptomycetaceae | Streptomyces |
| 25.95 | 25.27 | 5.21 | 4.85 | 1.22E-06 | 0.000119585 | Archaea | Halobacterota | Methanosarcinia | Methanosarciniales | Syntrophoarchaeaceae | NA |
| 22.72 | 23.69 | 4.92 | 4.81 | 1.49E-06 | 0.000141293 | Bacteria | Deinococcota | Deinococci | Thermales | Thermaceae | Thermus |
| 34.31 | 24.27 | 5.11 | 4.75 | 2.03E-06 | 0.00018374 | Archaea | Hydrothermarchaeota | Hydrothermarchaeia | Hydrothermarchaeales | NA | NA |
| 32.54 | 24.72 | 5.21 | 4.75 | 2.07E-06 | 0.00018374 | Bacteria | Firmicutes | Desulfotomaculia | Desulfotomaculales | Desulfallas-Sporotomaculum | BRH-c8a |
| 17.55 | 23.92 | 5.05 | 4.74 | 2.17E-06 | 0.000186931 | Bacteria | Acetothermia | Acetothermiia | NA | NA | NA |
| 12.59 | 23.48 | 5.01 | 4.69 | 2.78E-06 | 0.000232879 | Bacteria | Actinobacteriota | Actinobacteria | Micrococcales | Microbacteriaceae | Microcella |
| 12.76 | 23.35 | 5.00 | 4.67 | 3.03E-06 | 0.00024692 | Bacteria | Proteobacteria | Gammaproteobacteria | Alteromonadales | Shewanellaceae | Shewanella |
| 7.73 | 24.17 | 5.21 | 4.64 | 3.46E-06 | 0.000274099 | Archaea | Euryarchaeota | Thermococci | Thermococcales | Thermococcaceae | Thermococcus |
| 20.77 | 24.00 | 5.21 | 4.61 | 4.06E-06 | 0.000313181 | Bacteria | Actinobacteriota | RBG-16-55-12 | NA | NA | NA |
| 8.70 | 22.88 | 4.98 | 4.59 | 4.37E-06 | 0.000320067 | Bacteria | Proteobacteria | Alphaproteobacteria | Rhizobiales | Beijerinckiaceae | Salinarimonas |
| 16.26 | 23.93 | 5.21 | 4.59 | 4.34E-06 | 0.000320067 | Bacteria | Desulfobacterota | Desulfobacteria | Desulfatiglandales | Desulfatiglandaceae | Desulfatiglans |
| 15.39 | 23.81 | 5.21 | 4.57 | 4.84E-06 | 0.000346446 | Bacteria | Actinobacteriota | RBG-16-55-12 | NA | NA | NA |
| 9.00 | 23.75 | 5.21 | 4.56 | 5.12E-06 | 0.000357622 | Bacteria | Firmicutes | Desulfotomaculia | Desulfotomaculales | Desulfallas-Sporotomaculum | BRH-c8a |
| 13.74 | 23.66 | 5.21 | 4.54 | 5.56E-06 | 0.000378571 | Bacteria | Actinobacteriota | Thermoleophilia | Solirubrobacterales | Solirubrobacteraceae | Conexibacter |
| 11.58 | 23.63 | 5.21 | 4.54 | 5.68E-06 | 0.000378571 | Bacteria | Firmicutes | Desulfitobacteriia | Desulfitobacteriales | Desulfitobacteriaceae | Desulfosporosinus |
| 6.25 | 23.40 | 5.21 | 4.49 | 7.05E-06 | 0.000459354 | Bacteria | Caldisericota | Caldisericia | Caldisericales | Caldisericaceae | Caldisericum |
| 11.30 | 23.17 | 5.21 | 4.45 | 8.62E-06 | 0.000538155 | Bacteria | Firmicutes | Desulfotomaculia | Desulfotomaculales | Desulfallas-Sporotomaculum | BRH-c8a |
| 6.17 | 23.17 | 5.21 | 4.45 | 8.62E-06 | 0.000538155 | Bacteria | Proteobacteria | Alphaproteobacteria | Rhodobacterales | Rhodobacteraceae | Ruegeria |
| 7.36 | 23.07 | 5.21 | 4.43 | 9.41E-06 | 0.000563271 | Bacteria | Proteobacteria | Alphaproteobacteria | Rhizobiales | Rhizobiaceae | Chelativorans |
| 26.83 | 22.54 | 5.09 | 4.43 | 9.32E-06 | 0.000563271 | Bacteria | Acetothermia | Acetothermiia | NA | NA | NA |
| 9.61 | 22.93 | 5.21 | 4.40 | 1.07E-05 | 0.00062755 | Bacteria | Proteobacteria | Gammaproteobacteria | Oceanospirillales | Halomonadaceae | Halomonas |
| 9.04 | 22.82 | 5.21 | 4.38 | 1.17E-05 | 0.000675324 | Bacteria | Actinobacteriota | Acidimicrobiia | Microtrichales | Iamiaceae | Iamia |
| 11.48 | 22.14 | 5.21 | 4.25 | 2.12E-05 | 0.001195436 | Bacteria | Proteobacteria | Alphaproteobacteria | Rhodobacterales | Rhodobacteraceae | Ruegeria |
| 40.78 | 19.81 | 5.21 | 3.80 | 0.000144445 | 0.007993547 | Bacteria | Firmicutes | Thermaerobacteria | Thermaerobacterales | Thermaerobacteraceae | Thermaerobacter |
| **Drill core vs Seawater samples [22 ASVs]** | | | | | | | | | | | |
| 11.95 | -30.69 | 4.37 | -7.02 | 2.29E-12 | 6.53E-10 | Bacteria | Proteobacteria | Gammaproteobacteria | Alteromonadales | Pseudoalteromonadaceae | Pseudoalteromonas |
| 487.76 | 14.39 | 2.21 | 6.52 | 6.98E-11 | 9.94E-09 | Bacteria | Proteobacteria | Alphaproteobacteria | Sphingomonadales | Sphingomonadaceae | NA |
| 32.12 | 29.52 | 4.62 | 6.39 | 1.62E-10 | 1.54E-08 | Bacteria | Actinobacteriota | Actinobacteria | Micrococcales | Micrococcaceae | Pseudarthrobacter |
| 366.90 | 13.97 | 2.20 | 6.35 | 2.17E-10 | 1.54E-08 | Bacteria | Proteobacteria | Alphaproteobacteria | Sphingomonadales | Sphingomonadaceae | NA |
| 20.89 | 28.89 | 5.02 | 5.76 | 8.41E-09 | 3.90E-07 | Bacteria | Proteobacteria | Gammaproteobacteria | Alteromonadales | Alteromonadaceae | Glaciecola |
| 21.87 | 28.98 | 5.02 | 5.77 | 7.75E-09 | 3.90E-07 | Bacteria | Proteobacteria | Gammaproteobacteria | Oceanospirillales | Saccharospirillaceae | Oleispira |
| 15.69 | 28.58 | 4.98 | 5.74 | 9.59E-09 | 3.90E-07 | Bacteria | Proteobacteria | Alphaproteobacteria | Reyranellales | Reyranellaceae | Reyranella |
| 12.42 | 29.63 | 5.23 | 5.66 | 1.52E-08 | 5.40E-07 | Bacteria | Proteobacteria | Gammaproteobacteria | Burkholderiales | Comamonadaceae | Piscinibacter |
| 9.10 | 28.51 | 5.06 | 5.63 | 1.78E-08 | 5.65E-07 | Bacteria | Campilobacterota | Campylobacteria | Campylobacterales | Sulfurospirillaceae | Sulfurospirillum |
| 14.36 | 28.83 | 5.23 | 5.51 | 3.62E-08 | 1.03E-06 | Bacteria | Proteobacteria | Gammaproteobacteria | Alteromonadales | Psychromonadaceae | Psychromonas |
| 14.50 | 27.85 | 5.23 | 5.32 | 1.03E-07 | 2.67E-06 | Bacteria | Firmicutes | Bacilli | Bacillales | Bacillaceae | Geobacillus |
| 22.72 | 26.19 | 4.95 | 5.29 | 1.19E-07 | 2.83E-06 | Bacteria | Deinococcota | Deinococci | Thermales | Thermaceae | Thermus |
| 14.57 | 25.43 | 4.97 | 5.11 | 3.14E-07 | 6.89E-06 | Bacteria | Proteobacteria | Gammaproteobacteria | Oceanospirillales | Saccharospirillaceae | Oceaniserpentilla |
| 143.87 | 21.40 | 4.21 | 5.08 | 3.72E-07 | 7.57E-06 | Archaea | Thermoplasmatota | Thermoplasmata | NA | NA | NA |
| 92.04 | 21.99 | 4.43 | 4.96 | 6.93E-07 | 1.32E-05 | Archaea | Thermoplasmatota | Thermoplasmata | NA | NA | NA |
| 40.78 | 25.39 | 5.23 | 4.85 | 1.23E-06 | 2.18E-05 | Bacteria | Firmicutes | Thermaerobacteria | Thermaerobacterales | Thermaerobacteraceae | Thermaerobacter |
| 9.24 | 24.95 | 5.25 | 4.76 | 1.98E-06 | 3.32E-05 | Bacteria | Campilobacterota | Campylobacteria | Campylobacterales | Sulfurospirillaceae | Sulfurospirillum |
| 54.44 | -18.63 | 4.55 | -4.10 | 4.21E-05 | 0.000666998 | Bacteria | Proteobacteria | Gammaproteobacteria | Vibrionales | Vibrionaceae | Aliivibrio |
| 37.08 | -19.10 | 4.84 | -3.94 | 8.04E-05 | 0.001206637 | Bacteria | Proteobacteria | Gammaproteobacteria | Vibrionales | Vibrionaceae | Aliivibrio |
| 66.97 | 11.52 | 3.01 | 3.83 | 0.000129237 | 0.00184162 | Bacteria | Actinobacteriota | Actinobacteria | Micrococcales | Microbacteriaceae | Galbitalea |
| 34.12 | 10.54 | 3.00 | 3.51 | 0.000451281 | 0.006124533 | Bacteria | Proteobacteria | Alphaproteobacteria | Reyranellales | Reyranellaceae | Reyranella |
| 6.25 | 17.74 | 5.26 | 3.38 | 0.000736807 | 0.009545003 | Bacteria | Caldisericota | Caldisericia | Caldisericales | Caldisericaceae | Caldisericum |
| **Fumarole vs Drill core [14 ASVs]** | | | | | | | | | | | |
| 17.57 | -27.68 | 5.24 | -5.28 | 1.26E-07 | 1.44E-05 | Bacteria | Bacteroidota | Bacteroidia | Flavobacteriales | Flavobacteriaceae | NS4 marine group |
| 34.71 | -26.69 | 4.98 | -5.36 | 8.17E-08 | 1.44E-05 | Bacteria | Cyanobacteria | Cyanobacteriia | Synechococcales | Cyanobiaceae | Synechococcus CC9902 |
| 20.41 | -27.00 | 5.24 | -5.15 | 2.57E-07 | 1.95E-05 | Bacteria | Bacteroidota | Bacteroidia | Flavobacteriales | Flavobacteriaceae | NA |
| 487.76 | -12.83 | 3.00 | -4.28 | 1.91E-05 | 0.00087013 | Bacteria | Proteobacteria | Alphaproteobacteria | Sphingomonadales | Sphingomonadaceae | NA |
| 20.78 | -26.03 | 6.02 | -4.32 | 1.56E-05 | 0.00087013 | Bacteria | Proteobacteria | Alphaproteobacteria | Rhodobacterales | Rhodobacteraceae | Planktomarina |
| 366.90 | -12.42 | 2.99 | -4.15 | 3.36E-05 | 0.001095069 | Bacteria | Proteobacteria | Alphaproteobacteria | Sphingomonadales | Sphingomonadaceae | NA |
| 7.78 | 28.93 | 6.92 | 4.18 | 2.93E-05 | 0.001095069 | Bacteria | Bacteroidota | Bacteroidia | Cytophagales | Spirosomaceae | Spirosoma |
| 21.87 | -26.36 | 6.77 | -3.90 | 9.75E-05 | 0.002777361 | Bacteria | Proteobacteria | Gammaproteobacteria | Oceanospirillales | Saccharospirillaceae | Oleispira |
| 20.89 | -26.09 | 6.76 | -3.86 | 0.000113707 | 0.002880566 | Bacteria | Proteobacteria | Gammaproteobacteria | Alteromonadales | Alteromonadaceae | Glaciecola |
| 12.42 | -26.04 | 7.05 | -3.69 | 0.000223746 | 0.004547461 | Bacteria | Proteobacteria | Gammaproteobacteria | Burkholderiales | Comamonadaceae | Piscinibacter |
| 14.36 | -25.77 | 7.05 | -3.65 | 0.000259285 | 0.004547461 | Bacteria | Proteobacteria | Gammaproteobacteria | Alteromonadales | Psychromonadaceae | Psychromonas |
| 14.57 | -24.51 | 6.70 | -3.66 | 0.000255411 | 0.004547461 | Bacteria | Proteobacteria | Gammaproteobacteria | Oceanospirillales | Saccharospirillaceae | Oceaniserpentilla |
| 14.50 | -25.81 | 7.05 | -3.66 | 0.000254299 | 0.004547461 | Bacteria | Firmicutes | Bacilli | Bacillales | Bacillaceae | Geobacillus |
| 32.12 | -21.33 | 6.23 | -3.43 | 0.000612032 | 0.009967386 | Bacteria | Actinobacteriota | Actinobacteria | Micrococcales | Micrococcaceae | Pseudarthrobacter |
| **Fumarole vs Seawater samples [3 ASVs]** | | | | | | | | | | | |
| 34.71 | -27.42 | 5.70 | -4.81 | 1.54E-06 | 0.004504291 | Bacteria | Cyanobacteria | Cyanobacteriia | Synechococcales | Cyanobiaceae | Synechococcus CC9902 |
| 17.57 | -27.99 | 6.01 | -4.66 | 3.20E-06 | 0.004699087 | Bacteria | Bacteroidota | Bacteroidia | Flavobacteriales | Flavobacteriaceae | NS4 marine group |
| 20.41 | -27.38 | 6.01 | -4.55 | 5.26E-06 | 0.005146681 | Bacteria | Bacteroidota | Bacteroidia | Flavobacteriales | Flavobacteriaceae | NA |
| **DC_1 vs DC_2 [13 ASVs]** | | | | | | | | | | | |
| 125.22 | 26.87 | 5.56 | 4.84 | 1.33E-06 | 0.001939702 | Bacteria | Proteobacteria | Gammaproteobacteria | Alteromonadales | Marinobacteraceae | Marinobacter |
| 170.80 | 25.52 | 5.56 | 4.59 | 4.37E-06 | 0.001939702 | Bacteria | Proteobacteria | Alphaproteobacteria | Rhizobiales | Beijerinckiaceae | Salinarimonas |
| 179.72 | 25.26 | 5.56 | 4.55 | 5.48E-06 | 0.001939702 | Bacteria | Proteobacteria | Gammaproteobacteria | Thiomicrospirales | Thiomicrospiraceae | Thioalkalimicrobium |
| 133.28 | 25.63 | 5.56 | 4.61 | 3.98E-06 | 0.001939702 | Bacteria | Proteobacteria | Alphaproteobacteria | Rhizobiales | Beijerinckiaceae | Salinarimonas |
| 133.85 | 25.51 | 5.56 | 4.59 | 4.39E-06 | 0.001939702 | Bacteria | Proteobacteria | Gammaproteobacteria | Thiomicrospirales | Thiomicrospiraceae | Thioalkalimicrobium |
| 45.91 | 24.62 | 5.56 | 4.43 | 9.37E-06 | 0.002474141 | Bacteria | Proteobacteria | Alphaproteobacteria | Rhodobacterales | Rhodobacteraceae | Nioella |
| 62.10 | 24.57 | 5.56 | 4.42 | 9.78E-06 | 0.002474141 | Bacteria | Proteobacteria | Gammaproteobacteria | Alteromonadales | Marinobacteraceae | Marinobacter |
| 55.05 | 24.34 | 5.56 | 4.38 | 1.18E-05 | 0.002619487 | Bacteria | Firmicutes | Limnochordia | Limnochordales | Limnochordaceae | NA |
| 58.20 | 23.97 | 5.56 | 4.31 | 1.60E-05 | 0.002856264 | Bacteria | Proteobacteria | Gammaproteobacteria | Ectothiorhodospirales | Ectothiorhodospiraceae | Ectothiorhodospira |
| 40.78 | 23.96 | 5.56 | 4.31 | 1.61E-05 | 0.002856264 | Bacteria | Proteobacteria | Gammaproteobacteria | Ectothiorhodospirales | Ectothiorhodospiraceae | Ectothiorhodospira |
| 30.80 | 23.62 | 5.56 | 4.25 | 2.13E-05 | 0.003423319 | Bacteria | Proteobacteria | Alphaproteobacteria | Rhodobacterales | Rhodobacteraceae | Nioella |
| 22.18 | 23.18 | 5.56 | 4.17 | 3.02E-05 | 0.004117957 | Bacteria | Bacteroidota | Bacteroidia | Flavobacteriales | Flavobacteriaceae | Polaribacter |
| 44.33 | 23.22 | 5.56 | 4.18 | 2.93E-05 | 0.004117957 | Bacteria | Proteobacteria | Gammaproteobacteria | Alteromonadales | Marinobacteraceae | Marinobacter |
| **DC_1 vs DC_3 [21 ASVs]** | | | | | | | | | | | |
| 170.80 | 29.43 | 4.83 | 6.09 | 1.11E-09 | 2.07E-07 | Bacteria | Proteobacteria | Alphaproteobacteria | Rhizobiales | Beijerinckiaceae | Salinarimonas |
| 133.85 | 29.28 | 4.83 | 6.06 | 1.36E-09 | 2.07E-07 | Bacteria | Proteobacteria | Gammaproteobacteria | Thiomicrospirales | Thiomicrospiraceae | Thioalkalimicrobium |
| 125.22 | 28.11 | 4.83 | 5.82 | 5.94E-09 | 4.54E-07 | Bacteria | Proteobacteria | Gammaproteobacteria | Alteromonadales | Marinobacteraceae | Marinobacter |
| 133.28 | 28.32 | 4.83 | 5.86 | 4.58E-09 | 4.54E-07 | Bacteria | Proteobacteria | Alphaproteobacteria | Rhizobiales | Beijerinckiaceae | Salinarimonas |
| 58.20 | 27.50 | 4.83 | 5.69 | 1.26E-08 | 7.69E-07 | Bacteria | Proteobacteria | Gammaproteobacteria | Ectothiorhodospirales | Ectothiorhodospiraceae | Ectothiorhodospira |
| 55.05 | 27.11 | 4.83 | 5.61 | 2.00E-08 | 7.98E-07 | Bacteria | Firmicutes | Limnochordia | Limnochordales | Limnochordaceae | NA |
| 45.91 | 27.08 | 4.83 | 5.60 | 2.09E-08 | 7.98E-07 | Bacteria | Proteobacteria | Alphaproteobacteria | Rhodobacterales | Rhodobacteraceae | Nioella |
| 62.10 | 27.28 | 4.83 | 5.65 | 1.64E-08 | 7.98E-07 | Bacteria | Proteobacteria | Gammaproteobacteria | Alteromonadales | Marinobacteraceae | Marinobacter |
| 40.78 | 26.73 | 4.83 | 5.53 | 3.16E-08 | 1.08E-06 | Bacteria | Proteobacteria | Gammaproteobacteria | Ectothiorhodospirales | Ectothiorhodospiraceae | Ectothiorhodospira |
| 44.33 | 26.55 | 4.83 | 5.50 | 3.88E-08 | 1.19E-06 | Bacteria | Proteobacteria | Gammaproteobacteria | Alteromonadales | Marinobacteraceae | Marinobacter |
| 30.80 | 26.27 | 4.83 | 5.44 | 5.41E-08 | 1.51E-06 | Bacteria | Proteobacteria | Alphaproteobacteria | Rhodobacterales | Rhodobacteraceae | Nioella |
| 22.18 | 25.93 | 4.83 | 5.37 | 8.04E-08 | 2.05E-06 | Bacteria | Bacteroidota | Bacteroidia | Flavobacteriales | Flavobacteriaceae | Polaribacter |
| 235.28 | -23.52 | 4.98 | -4.72 | 2.35E-06 | 5.53E-05 | Bacteria | Firmicutes | Bacilli | Bacillales | Bacillaceae | Geobacillus |
| 34.81 | -19.64 | 4.42 | -4.44 | 8.88E-06 | 0.000194167 | Bacteria | Bacteroidota | Bacteroidia | Flavobacteriales | Flavobacteriaceae | Flavicella |
| 20.04 | -19.24 | 4.98 | -3.86 | 0.000113333 | 0.002311997 | Bacteria | Proteobacteria | Gammaproteobacteria | Arenicellales | Arenicellaceae | Arenicella |
| 31.71 | -18.33 | 4.83 | -3.79 | 0.000148953 | 0.002848717 | Bacteria | Proteobacteria | Alphaproteobacteria | Reyranellales | Reyranellaceae | Reyranella |
| 26.05 | -18.73 | 4.98 | -3.76 | 0.000170847 | 0.003075248 | Bacteria | Proteobacteria | Gammaproteobacteria | Alteromonadales | Psychromonadaceae | Psychromonas |
| 20.51 | -18.63 | 4.98 | -3.74 | 0.000185677 | 0.003156502 | Bacteria | Proteobacteria | Alphaproteobacteria | Rhodobacterales | Rhodobacteraceae | Sulfitobacter |
| 9.98 | -18.12 | 4.98 | -3.64 | 0.000276345 | 0.004450606 | Bacteria | Firmicutes | Bacilli | Bacillales | Bacillaceae | Geobacillus |
| 17.86 | -17.58 | 4.98 | -3.53 | 0.000419387 | 0.006416625 | Bacteria | Proteobacteria | Alphaproteobacteria | Rhizobiales | Xanthobacteraceae | NA |
| **DC_1 vs DC_4 [20 ASVs]** | | | | | | | | | | | |
| 179.72 | 29.54 | 5.06 | 5.84 | 5.25E-09 | 8.35E-07 | Bacteria | Proteobacteria | Gammaproteobacteria | Thiomicrospirales | Thiomicrospiraceae | Thioalkalimicrobium |
| 133.28 | 28.22 | 5.06 | 5.58 | 2.45E-08 | 1.30E-06 | Bacteria | Proteobacteria | Alphaproteobacteria | Rhizobiales | Beijerinckiaceae | Salinarimonas |
| 133.85 | 28.24 | 5.06 | 5.58 | 2.38E-08 | 1.30E-06 | Bacteria | Proteobacteria | Gammaproteobacteria | Thiomicrospirales | Thiomicrospiraceae | Thioalkalimicrobium |
| 58.20 | 27.40 | 5.06 | 5.41 | 6.14E-08 | 1.95E-06 | Bacteria | Proteobacteria | Gammaproteobacteria | Ectothiorhodospirales | Ectothiorhodospiraceae | Ectothiorhodospira |
| 125.22 | 27.45 | 5.06 | 5.43 | 5.76E-08 | 1.95E-06 | Bacteria | Proteobacteria | Gammaproteobacteria | Alteromonadales | Marinobacteraceae | Marinobacter |
| 55.05 | 27.07 | 5.06 | 5.35 | 8.79E-08 | 2.00E-06 | Bacteria | Firmicutes | Limnochordia | Limnochordales | Limnochordaceae | NA |
| 62.10 | 27.15 | 5.06 | 5.37 | 8.08E-08 | 2.00E-06 | Bacteria | Proteobacteria | Gammaproteobacteria | Alteromonadales | Marinobacteraceae | Marinobacter |
| 45.91 | 26.64 | 5.06 | 5.26 | 1.41E-07 | 2.49E-06 | Bacteria | Proteobacteria | Alphaproteobacteria | Rhodobacterales | Rhodobacteraceae | Nioella |
| 40.78 | 26.64 | 5.06 | 5.26 | 1.41E-07 | 2.49E-06 | Bacteria | Proteobacteria | Gammaproteobacteria | Ectothiorhodospirales | Ectothiorhodospiraceae | Ectothiorhodospira |
| 30.80 | 26.24 | 5.06 | 5.19 | 2.14E-07 | 3.41E-06 | Bacteria | Proteobacteria | Alphaproteobacteria | Rhodobacterales | Rhodobacteraceae | Nioella |
| 44.33 | 26.15 | 5.06 | 5.17 | 2.37E-07 | 3.43E-06 | Bacteria | Proteobacteria | Gammaproteobacteria | Alteromonadales | Marinobacteraceae | Marinobacter |
| 22.18 | 25.69 | 5.06 | 5.08 | 3.83E-07 | 5.08E-06 | Bacteria | Bacteroidota | Bacteroidia | Flavobacteriales | Flavobacteriaceae | Polaribacter |
| 48.37 | 23.55 | 4.75 | 4.96 | 6.96E-07 | 8.51E-06 | Bacteria | Proteobacteria | Gammaproteobacteria | Oceanospirillales | Saccharospirillaceae | Oleispira |
| 34.81 | -19.33 | 4.59 | -4.21 | 2.58E-05 | 0.000293226 | Bacteria | Bacteroidota | Bacteroidia | Flavobacteriales | Flavobacteriaceae | Flavicella |
| 26.05 | -19.68 | 5.18 | -3.80 | 0.000146544 | 0.001553369 | Bacteria | Proteobacteria | Gammaproteobacteria | Alteromonadales | Psychromonadaceae | Psychromonas |
| 20.51 | -19.37 | 5.18 | -3.74 | 0.000185936 | 0.001847741 | Bacteria | Proteobacteria | Alphaproteobacteria | Rhodobacterales | Rhodobacteraceae | Sulfitobacter |
| 17.86 | -18.34 | 5.18 | -3.54 | 0.000402398 | 0.003763605 | Bacteria | Proteobacteria | Alphaproteobacteria | Rhizobiales | Xanthobacteraceae | NA |
| 20.04 | -18.08 | 5.18 | -3.49 | 0.000486846 | 0.004300475 | Bacteria | Proteobacteria | Gammaproteobacteria | Arenicellales | Arenicellaceae | Arenicella |
| 9.98 | -17.19 | 5.19 | -3.31 | 0.000918291 | 0.007684642 | Bacteria | Firmicutes | Bacilli | Bacillales | Bacillaceae | Geobacillus |
| 31.71 | -16.26 | 5.03 | -3.23 | 0.001229375 | 0.00977353 | Bacteria | Proteobacteria | Alphaproteobacteria | Reyranellales | Reyranellaceae | Reyranella |
| **DC_2 vs DC_3 [6 ASVs]** | | | | | | | | | | | |
| 34.81 | -33.43 | 3.78 | -8.83 | 1.03E-18 | 1.82E-15 | Bacteria | Bacteroidota | Bacteroidia | Flavobacteriales | Flavobacteriaceae | Flavicella |
| 9.98 | -33.64 | 4.27 | -7.87 | 3.45E-15 | 3.05E-12 | Bacteria | Firmicutes | Bacilli | Bacillales | Bacillaceae | Geobacillus |
| 20.04 | -32.05 | 4.27 | -7.50 | 6.19E-14 | 3.65E-11 | Bacteria | Proteobacteria | Gammaproteobacteria | Arenicellales | Arenicellaceae | Arenicella |
| 235.28 | -31.30 | 4.27 | -7.33 | 2.30E-13 | 1.02E-10 | Bacteria | Firmicutes | Bacilli | Bacillales | Bacillaceae | Geobacillus |
| 26.05 | -30.22 | 4.27 | -7.07 | 1.51E-12 | 5.35E-10 | Bacteria | Proteobacteria | Gammaproteobacteria | Alteromonadales | Psychromonadaceae | Psychromonas |
| 20.51 | -28.96 | 4.27 | -6.78 | 1.20E-11 | 3.55E-09 | Bacteria | Proteobacteria | Alphaproteobacteria | Rhodobacterales | Rhodobacteraceae | Sulfitobacter |
| **DC_2 vs DC_4 [6 ASVs]** | | | | | | | | | | | |
| 34.81 | -33.11 | 3.98 | -8.31 | 9.60E-17 | 1.70E-13 | Bacteria | Bacteroidota | Bacteroidia | Flavobacteriales | Flavobacteriaceae | Flavicella |
| 9.98 | -32.70 | 4.51 | -7.26 | 3.93E-13 | 3.48E-10 | Bacteria | Firmicutes | Bacilli | Bacillales | Bacillaceae | Geobacillus |
| 26.05 | -31.16 | 4.50 | -6.92 | 4.45E-12 | 2.63E-09 | Bacteria | Proteobacteria | Gammaproteobacteria | Alteromonadales | Psychromonadaceae | Psychromonas |
| 20.04 | -30.90 | 4.50 | -6.86 | 6.86E-12 | 3.04E-09 | Bacteria | Proteobacteria | Gammaproteobacteria | Arenicellales | Arenicellaceae | Arenicella |
| 20.51 | -29.70 | 4.50 | -6.60 | 4.17E-11 | 1.48E-08 | Bacteria | Proteobacteria | Alphaproteobacteria | Rhodobacterales | Rhodobacteraceae | Sulfitobacter |
| 48.37 | 24.26 | 4.17 | 5.82 | 5.83E-09 | 1.72E-06 | Bacteria | Proteobacteria | Gammaproteobacteria | Oceanospirillales | Saccharospirillaceae | Oleispira |
| **DC_3 vs DC_4 [4 ASVs]** | | | | | | | | | | | |
| 235.28 | 36.91 | 3.60 | 10.25 | 1.17E-24 | 2.06E-21 | Bacteria | Firmicutes | Bacilli | Bacillales | Bacillaceae | Geobacillus |
| 48.37 | 23.77 | 3.38 | 7.03 | 2.11E-12 | 1.87E-09 | Bacteria | Proteobacteria | Gammaproteobacteria | Oceanospirillales | Saccharospirillaceae | Oleispira |
| 179.72 | 20.67 | 3.61 | 5.72 | 1.06E-08 | 6.29E-06 | Bacteria | Proteobacteria | Gammaproteobacteria | Thiomicrospirales | Thiomicrospiraceae | Thioalkalimicrobium |
| 170.80 | -17.56 | 3.66 | -4.80 | 1.57E-06 | 0.000694532 | Bacteria | Proteobacteria | Alphaproteobacteria | Rhizobiales | Beijerinckiaceae | Salinarimonas |
| **Archaeal primer sets:** | | | | | | | | | | | |
| **DC_2 vs DC_3 [17 ASVs]** | | | | | | | | | | | |
| 5997.76 | 22.11 | 3.11 | 7.10 | 1.20E-12 | 1.01E-10 | Archaea | Crenarchaeota | Nitrososphaeria | Nitrosopumilales | Nitrosopumilaceae | Candidatus Nitrosopumilus |
| 757.70 | 28.48 | 4.83 | 5.90 | 3.74E-09 | 1.57E-07 | Archaea | Thermoplasmatota | Thermoplasmata | Marine Group II | NA | NA |
| 402.22 | 27.36 | 4.83 | 5.66 | 1.49E-08 | 4.16E-07 | Archaea | Thermoplasmatota | Thermoplasmata | Marine Group II | NA | NA |
| 243.35 | 26.91 | 4.83 | 5.57 | 2.55E-08 | 5.35E-07 | Archaea | Thermoplasmatota | Thermoplasmata | Marine Group II | NA | NA |
| 117.57 | 25.97 | 4.83 | 5.38 | 7.65E-08 | 1.28E-06 | Archaea | Thermoplasmatota | Thermoplasmata | Marine Group II | NA | NA |
| 73.65 | 25.57 | 4.83 | 5.29 | 1.21E-07 | 1.69E-06 | Archaea | Thermoplasmatota | Thermoplasmata | Marine Group II | NA | NA |
| 182.68 | -25.56 | 4.98 | -5.13 | 2.90E-07 | 3.48E-06 | Archaea | Thermoplasmatota | Thermoplasmata | NA | NA | NA |
| 1023.46 | 24.37 | 4.83 | 5.05 | 4.53E-07 | 4.76E-06 | Archaea | Thermoplasmatota | Thermoplasmata | Marine Group II | NA | NA |
| 36.95 | -24.30 | 4.98 | -4.88 | 1.09E-06 | 1.01E-05 | Archaea | Crenarchaeota | Bathyarchaeia | NA | NA | NA |
| 33.16 | -24.12 | 4.98 | -4.84 | 1.29E-06 | 1.05E-05 | Archaea | Thermoplasmatota | Thermoplasmata | NA | NA | NA |
| 504.49 | 23.32 | 4.83 | 4.83 | 1.38E-06 | 1.05E-05 | Archaea | Thermoplasmatota | Thermoplasmata | Marine Group II | NA | NA |
| 38.44 | -23.95 | 4.98 | -4.81 | 1.54E-06 | 1.08E-05 | Archaea | Thermoplasmatota | Thermoplasmata | NA | NA | NA |
| 27.35 | -23.53 | 4.98 | -4.72 | 2.34E-06 | 1.51E-05 | Archaea | Crenarchaeota | Bathyarchaeia | NA | NA | NA |
| 24.06 | -23.26 | 4.98 | -4.67 | 3.05E-06 | 1.83E-05 | Archaea | Euryarchaeota | Thermococci | Thermococcales | Thermococcaceae | Thermococcus |
| 1476.38 | 20.07 | 4.41 | 4.55 | 5.30E-06 | 2.97E-05 | Archaea | Crenarchaeota | Nitrososphaeria | Nitrosopumilales | Nitrosopumilaceae | Candidatus Nitrosopumilus |
| 745.24 | 19.34 | 4.27 | 4.53 | 5.78E-06 | 3.03E-05 | Archaea | Crenarchaeota | Nitrososphaeria | Nitrosopumilales | Nitrosopumilaceae | Candidatus Nitrosopumilus |
| 530.54 | 18.38 | 4.24 | 4.33 | 1.49E-05 | 7.38E-05 | Archaea | Crenarchaeota | Nitrososphaeria | Nitrosopumilales | Nitrosopumilaceae | Candidatus Nitrosopumilus |
| **DC_2 vs DC_3 [5 ASVs]** | | | | | | | | | | | |
| 757.70 | 39.90 | 5.06 | 7.89 | 3.12E-15 | 2.62E-13 | Archaea | Thermoplasmatota | Thermoplasmata | Marine Group II | NA | NA |
| 243.35 | 34.42 | 5.06 | 6.80 | 1.02E-11 | 4.30E-10 | Archaea | Thermoplasmatota | Thermoplasmata | Marine Group II | NA | NA |
| 402.22 | 33.75 | 5.06 | 6.67 | 2.57E-11 | 7.19E-10 | Archaea | Thermoplasmatota | Thermoplasmata | Marine Group II | NA | NA |
| 117.57 | 29.84 | 5.06 | 5.90 | 3.69E-09 | 6.20E-08 | Archaea | Thermoplasmatota | Thermoplasmata | Marine Group II | NA | NA |
| 73.65 | 29.88 | 5.06 | 5.91 | 3.50E-09 | 6.20E-08 | Archaea | Thermoplasmatota | Thermoplasmata | Marine Group II | NA | NA |
| **DC_2 vs DC_4 [5 ASVs]** | | | | | | | | | | | |
| 757.70 | 35.50 | 5.56 | 6.39 | 1.66E-10 | 1.40E-08 | Archaea | Thermoplasmatota | Thermoplasmata | Marine Group II | NA | NA |
| 402.22 | 34.83 | 5.56 | 6.27 | 3.65E-10 | 1.53E-08 | Archaea | Thermoplasmatota | Thermoplasmata | Marine Group II | NA | NA |
| 243.35 | 34.08 | 5.56 | 6.13 | 8.55E-10 | 2.39E-08 | Archaea | Thermoplasmatota | Thermoplasmata | Marine Group II | NA | NA |
| 117.57 | 33.01 | 5.56 | 5.94 | 2.82E-09 | 5.93E-08 | Archaea | Thermoplasmatota | Thermoplasmata | Marine Group II | NA | NA |
| 73.65 | 30.75 | 5.56 | 5.53 | 3.11E-08 | 5.23E-07 | Archaea | Thermoplasmatota | Thermoplasmata | Marine Group II | NA | NA |
| **DC_3 vs BF [12 ASVs]** | | | | | | | | | | | |
| 5997.76 | 28.97 | 2.33 | 12.42 | 2.09E-35 | 1.75E-33 | Archaea | Crenarchaeota | Nitrososphaeria | Nitrosopumilales | Nitrosopumilaceae | Candidatus Nitrosopumilus |
| 1476.38 | 27.04 | 3.26 | 8.29 | 1.13E-16 | 4.21E-15 | Archaea | Crenarchaeota | Nitrososphaeria | Nitrosopumilales | Nitrosopumilaceae | Candidatus Nitrosopumilus |
| 745.24 | 26.07 | 3.16 | 8.26 | 1.50E-16 | 4.21E-15 | Archaea | Crenarchaeota | Nitrososphaeria | Nitrosopumilales | Nitrosopumilaceae | Candidatus Nitrosopumilus |
| 530.54 | 25.64 | 3.14 | 8.16 | 3.28E-16 | 6.89E-15 | Archaea | Crenarchaeota | Nitrososphaeria | Nitrosopumilales | Nitrosopumilaceae | Candidatus Nitrosopumilus |
| 182.68 | -27.07 | 3.60 | -7.52 | 5.56E-14 | 9.34E-13 | Archaea | Thermoplasmatota | Thermoplasmata | NA | NA | NA |
| 1023.46 | 26.28 | 3.57 | 7.37 | 1.76E-13 | 2.46E-12 | Archaea | Thermoplasmatota | Thermoplasmata | Marine Group II | NA | NA |
| 504.49 | 25.39 | 3.57 | 7.12 | 1.10E-12 | 1.32E-11 | Archaea | Thermoplasmatota | Thermoplasmata | Marine Group II | NA | NA |
| 27.35 | -24.22 | 3.60 | -6.72 | 1.77E-11 | 1.63E-10 | Archaea | Crenarchaeota | Bathyarchaeia | NA | NA | NA |
| 33.16 | -24.24 | 3.60 | -6.73 | 1.70E-11 | 1.63E-10 | Archaea | Thermoplasmatota | Thermoplasmata | NA | NA | NA |
| 38.44 | -24.17 | 3.60 | -6.71 | 1.94E-11 | 1.63E-10 | Archaea | Thermoplasmatota | Thermoplasmata | NA | NA | NA |
| 36.95 | -23.77 | 3.60 | -6.60 | 4.12E-11 | 3.14E-10 | Archaea | Crenarchaeota | Bathyarchaeia | NA | NA | NA |
| 24.06 | -23.61 | 3.60 | -6.56 | 5.56E-11 | 3.89E-10 | Archaea | Euryarchaeota | Thermococci | Thermococcales | Thermococcaceae | Thermococcus |
| **DC_4 vs BF [21 ASVs]** | | | | | | | | | | | |
| 5997.76 | 26.23 | 2.71 | 9.69 | 3.32E-22 | 1.50E-20 | Archaea | Crenarchaeota | Nitrososphaeria | Nitrosopumilales | Nitrosopumilaceae | Candidatus Nitrosopumilus |
| 182.68 | -28.37 | 4.27 | -6.64 | 3.08E-11 | 6.92E-10 | Archaea | Thermoplasmatota | Thermoplasmata | NA | NA | NA |
| 745.24 | 23.63 | 3.69 | 6.40 | 1.52E-10 | 2.29E-09 | Archaea | Crenarchaeota | Nitrososphaeria | Nitrosopumilales | Nitrosopumilaceae | Candidatus Nitrosopumilus |
| 1476.38 | 23.65 | 3.81 | 6.20 | 5.68E-10 | 5.11E-09 | Archaea | Crenarchaeota | Nitrososphaeria | Nitrosopumilales | Nitrosopumilaceae | Candidatus Nitrosopumilus |
| 530.54 | 22.86 | 3.67 | 6.23 | 4.71E-10 | 5.11E-09 | Archaea | Crenarchaeota | Nitrososphaeria | Nitrosopumilales | Nitrosopumilaceae | Candidatus Nitrosopumilus |
| 38.44 | -25.85 | 4.27 | -6.05 | 1.43E-09 | 9.21E-09 | Archaea | Thermoplasmatota | Thermoplasmata | NA | NA | NA |
| 36.95 | -25.92 | 4.27 | -6.07 | 1.30E-09 | 9.21E-09 | Archaea | Crenarchaeota | Bathyarchaeia | NA | NA | NA |
| 33.16 | -25.36 | 4.27 | -5.94 | 2.91E-09 | 1.64E-08 | Archaea | Thermoplasmatota | Thermoplasmata | NA | NA | NA |
| 24.06 | -25.26 | 4.27 | -5.91 | 3.38E-09 | 1.69E-08 | Archaea | Euryarchaeota | Thermococci | Thermococcales | Thermococcaceae | Thermococcus |
| 27.35 | -25.03 | 4.27 | -5.86 | 4.64E-09 | 2.09E-08 | Archaea | Crenarchaeota | Bathyarchaeia | NA | NA | NA |
| 1023.46 | 23.74 | 4.18 | 5.68 | 1.31E-08 | 5.36E-08 | Archaea | Thermoplasmatota | Thermoplasmata | Marine Group II | NA | NA |
| 504.49 | 22.46 | 4.18 | 5.38 | 7.50E-08 | 2.81E-07 | Archaea | Thermoplasmatota | Thermoplasmata | Marine Group II | NA | NA |
| 147.09 | -13.62 | 4.27 | -3.19 | 0.00142464 | 0.004931448 | Archaea | Thermoplasmatota | NA | NA | NA | NA |
| 127.58 | -13.42 | 4.27 | -3.14 | 0.001680821 | 0.00540264 | Archaea | Thermoplasmatota | Thermoplasmata | NA | NA | NA |
| 113.67 | -13.25 | 4.27 | -3.10 | 0.001919082 | 0.005757246 | Archaea | Thermoplasmatota | Thermoplasmata | NA | NA | NA |
| 89.56 | -12.91 | 4.27 | -3.02 | 0.002512326 | 0.007065918 | Archaea | Hydrothermarchaeota | Hydrothermarchaeia | Hydrothermarchaeales | NA | NA |
| 73.38 | -12.62 | 4.27 | -2.95 | 0.003131881 | 0.007829702 | Archaea | Hydrothermarchaeota | Hydrothermarchaeia | Hydrothermarchaeales | NA | NA |
| 76.18 | -12.67 | 4.27 | -2.97 | 0.003005953 | 0.007829702 | Archaea | Hydrothermarchaeota | Hydrothermarchaeia | Hydrothermarchaeales | NA | NA |
| 352.18 | 12.17 | 4.18 | 2.91 | 0.003576005 | 0.008469485 | Archaea | Thermoplasmatota | Thermoplasmata | Marine Group II | NA | NA |
| 56.45 | -12.24 | 4.27 | -2.87 | 0.004159835 | 0.00935963 | Archaea | Hydrothermarchaeota | Hydrothermarchaeia | Hydrothermarchaeales | NA | NA |
| 24.32 | -11.03 | 3.89 | -2.83 | 0.004604425 | 0.009866625 | Archaea | Euryarchaeota | Thermococci | Thermococcales | Thermococcaceae | Thermococcus |

**Table S6**. Putative contaminant ASVs removed from analyses using universal primer set [336] that were identify by overlap approach among seawater samples, drilling fluid, drill core from the subaerial deposit and from the submarine deposit (See Figure 2). NA., Not assigned.

| Kingdom | Phylum | Class | Order | Family | Genus |
| --- | --- | --- | --- | --- | --- |
| **202 ASVs (93 + 84 + 14 + 11) shared between drilling fluid and drill cores from the subaerial deposit.** | | | | | |
| Bacteria | Proteobacteria | Alphaproteobacteria | SAR11 clade | Clade I | Clade Ia |
| Archaea | Thermoplasmatota | Thermoplasmata | Marine Group II | NA | NA |
| Bacteria | Proteobacteria | Alphaproteobacteria | Micavibrionales | NA | NA |
| Bacteria | Proteobacteria | Gammaproteobacteria | Thiotrichales | Thiotrichaceae | NA |
| Bacteria | Bacteroidota | Bacteroidia | Flavobacteriales | NS9 marine group | NA |
| Bacteria | Planctomycetota | Planctomycetes | Pirellulales | Pirellulaceae | Rubripirellula |
| Bacteria | Proteobacteria | Alphaproteobacteria | SAR11 clade | Clade II | NA |
| Bacteria | Proteobacteria | Gammaproteobacteria | Alteromonadales | Psychromonadaceae | Psychromonas |
| Bacteria | Proteobacteria | Gammaproteobacteria | Alteromonadales | Pseudoalteromonadaceae | Pseudoalteromonas |
| Bacteria | Proteobacteria | Gammaproteobacteria | Steroidobacterales | Woeseiaceae | Woeseia |
| Bacteria | Planctomycetota | OM190 | NA | NA | NA |
| Bacteria | Bacteroidota | Bacteroidia | Flavobacteriales | Flavobacteriaceae | NS5 marine group |
| Bacteria | Proteobacteria | Gammaproteobacteria | Cellvibrionales | Halieaceae | OM60(NOR5) clade |
| Bacteria | Proteobacteria | Alphaproteobacteria | Rhodobacterales | Rhodobacteraceae | Amylibacter |
| Bacteria | Proteobacteria | Alphaproteobacteria | Micavibrionales | NA | NA |
| Bacteria | Cyanobacteria | Cyanobacteriia | Synechococcales | Cyanobiaceae | Synechococcus CC9902 |
| Bacteria | Bacteroidota | Bacteroidia | Flavobacteriales | Flavobacteriaceae | Winogradskyella |
| Bacteria | Proteobacteria | Alphaproteobacteria | Rhodobacterales | Rhodobacteraceae | Litoreibacter |
| Bacteria | Proteobacteria | Gammaproteobacteria | Oceanospirillales | Saccharospirillaceae | Oleibacter |
| Bacteria | Proteobacteria | Gammaproteobacteria | Oceanospirillales | Saccharospirillaceae | Oleibacter |
| Bacteria | Proteobacteria | Alphaproteobacteria | Parvibaculales | OCS116 clade | NA |
| Bacteria | Proteobacteria | Gammaproteobacteria | Alteromonadales | Pseudoalteromonadaceae | Psychrosphaera |
| Bacteria | Proteobacteria | Gammaproteobacteria | Alteromonadales | Alteromonadaceae | Alteromonas |
| Bacteria | Proteobacteria | Alphaproteobacteria | SAR11 clade | Clade IV | NA |
| Bacteria | Fusobacteriota | Fusobacteriia | Fusobacteriales | Fusobacteriaceae | Psychrilyobacter |
| Bacteria | Bacteroidota | Bacteroidia | Flavobacteriales | Flavobacteriaceae | Olleya |
| Bacteria | Bacteroidota | Bacteroidia | Flavobacteriales | Flavobacteriaceae | NS4 marine group |
| Bacteria | Verrucomicrobiota | Verrucomicrobiae | Verrucomicrobiales | Rubritaleaceae | Roseibacillus |
| Bacteria | Proteobacteria | Gammaproteobacteria | SAR86 clade | NA | NA |
| Bacteria | Proteobacteria | Gammaproteobacteria | Cellvibrionales | Halieaceae | OM60(NOR5) clade |
| Bacteria | Bacteroidota | Bacteroidia | Flavobacteriales | Flavobacteriaceae | Formosa |
| Bacteria | Bacteroidota | Bacteroidia | Flavobacteriales | Flavobacteriaceae | Flavicella |
| Archaea | Crenarchaeota | Nitrososphaeria | Nitrosopumilales | Nitrosopumilaceae | Candidatus Nitrosopumilus |
| Bacteria | Proteobacteria | Gammaproteobacteria | Alteromonadales | Alteromonadaceae | Alteromonas |
| Bacteria | Actinobacteriota | Acidimicrobiia | Actinomarinales | Actinomarinaceae | Candidatus Actinomarina |
| Bacteria | Proteobacteria | Gammaproteobacteria | Oceanospirillales | Pseudohongiellaceae | Pseudohongiella |
| Bacteria | Bacteroidota | Bacteroidia | Flavobacteriales | Cryomorphaceae | Vicingus |
| Bacteria | Bacteroidota | Bacteroidia | Flavobacteriales | Flavobacteriaceae | Formosa |
| Bacteria | Bacteroidota | Bacteroidia | Flavobacteriales | Cryomorphaceae | NA |
| Archaea | Crenarchaeota | Nitrososphaeria | Nitrosopumilales | Nitrosopumilaceae | Candidatus Nitrosopumilus |
| Bacteria | Proteobacteria | Gammaproteobacteria | NA | NA | NA |
| Bacteria | Proteobacteria | Gammaproteobacteria | Arenicellales | Arenicellaceae | Arenicella |
| Archaea | Thermoplasmatota | Thermoplasmata | Marine Group II | NA | NA |
| Bacteria | Bacteroidota | Bacteroidia | Flavobacteriales | Flavobacteriaceae | NS5 marine group |
| Bacteria | Proteobacteria | Gammaproteobacteria | Thiotrichales | Thiotrichaceae | Leucothrix |
| Bacteria | Bacteroidota | Bacteroidia | Flavobacteriales | Cryomorphaceae | Vicingus |
| Bacteria | Bacteroidota | Bacteroidia | Flavobacteriales | NS9 marine group | NA |
| Bacteria | Bacteroidota | Bacteroidia | Flavobacteriales | Flavobacteriaceae | NS5 marine group |
| Bacteria | Verrucomicrobiota | Verrucomicrobiae | Opitutales | Puniceicoccaceae | Lentimonas |
| Bacteria | Proteobacteria | Gammaproteobacteria | Alteromonadales | Alteromonadaceae | Aliiglaciecola |
| Bacteria | Proteobacteria | Alphaproteobacteria | Rhodobacterales | Rhodobacteraceae | Pseudophaeobacter |
| Bacteria | Bacteroidota | Bacteroidia | Flavobacteriales | Flavobacteriaceae | Formosa |
| Bacteria | Marinimicrobia (SAR406 clade) | NA | NA | NA | NA |
| Bacteria | Proteobacteria | Alphaproteobacteria | SAR11 clade | Clade I | Clade Ia |
| Bacteria | Proteobacteria | Gammaproteobacteria | Granulosicoccales | Granulosicoccaceae | Granulosicoccus |
| Bacteria | Proteobacteria | Gammaproteobacteria | Cellvibrionales | Porticoccaceae | SAR92 clade |
| Bacteria | Bacteroidota | Bacteroidia | Flavobacteriales | Flavobacteriaceae | Aurantivirga |
| Archaea | Crenarchaeota | Nitrososphaeria | Nitrosopumilales | Nitrosopumilaceae | Candidatus Nitrosopumilus |
| Bacteria | Verrucomicrobiota | Verrucomicrobiae | Verrucomicrobiales | Rubritaleaceae | Persicirhabdus |
| Bacteria | Proteobacteria | Gammaproteobacteria | Thiotrichales | Thiotrichaceae | Leucothrix |
| Bacteria | Proteobacteria | Alphaproteobacteria | Micavibrionales | NA | NA |
| Bacteria | Proteobacteria | Alphaproteobacteria | Micavibrionales | Micavibrionaceae | NA |
| Bacteria | Bacteroidota | Bacteroidia | Flavobacteriales | NS9 marine group | NA |
| Bacteria | Actinobacteriota | Acidimicrobiia | Microtrichales | Microtrichaceae | Sva0996 marine group |
| Bacteria | Bacteroidota | Bacteroidia | Flavobacteriales | Cryomorphaceae | NA |
| Bacteria | Bacteroidota | Bacteroidia | Sphingobacteriales | NS11-12 marine group | NA |
| Bacteria | Proteobacteria | Gammaproteobacteria | SAR86 clade | NA | NA |
| Bacteria | Proteobacteria | Gammaproteobacteria | Burkholderiales | Methylophilaceae | OM43 clade |
| Bacteria | Proteobacteria | Gammaproteobacteria | Thiomicrospirales | Thioglobaceae | SUP05 cluster |
| Bacteria | Proteobacteria | Alphaproteobacteria | Rhodobacterales | Rhodobacteraceae | Sulfitobacter |
| Bacteria | Proteobacteria | Alphaproteobacteria | Parvibaculales | PS1 clade | NA |
| Bacteria | Proteobacteria | Alphaproteobacteria | Rhizobiales | Devosiaceae | Maritalea |
| Bacteria | Proteobacteria | Gammaproteobacteria | Ectothiorhodospirales | Ectothiorhodospiraceae | NA |
| Bacteria | Proteobacteria | Gammaproteobacteria | Vibrionales | Vibrionaceae | Aliivibrio |
| Bacteria | Planctomycetota | Phycisphaerae | Phycisphaerales | Phycisphaeraceae | CL500-3 |
| Bacteria | Proteobacteria | Gammaproteobacteria | Alteromonadales | Colwelliaceae | Colwellia |
| Bacteria | Bacteroidota | Bacteroidia | Flavobacteriales | Flavobacteriaceae | Nonlabens |
| Bacteria | Verrucomicrobiota | Verrucomicrobiae | Verrucomicrobiales | Rubritaleaceae | Rubritalea |
| Bacteria | Proteobacteria | Gammaproteobacteria | Alteromonadales | Colwelliaceae | Thalassotalea |
| Bacteria | Verrucomicrobiota | Verrucomicrobiae | Verrucomicrobiales | Rubritaleaceae | Rubritalea |
| Bacteria | Bacteroidota | Bacteroidia | Flavobacteriales | Flavobacteriaceae | Polaribacter |
| Bacteria | Proteobacteria | Alphaproteobacteria | Rhodobacterales | Rhodobacteraceae | Sulfitobacter |
| Bacteria | Cyanobacteria | Cyanobacteriia | Synechococcales | Cyanobiaceae | Synechococcus CC9902 |
| Bacteria | Proteobacteria | Alphaproteobacteria | SAR11 clade | Clade I | Clade Ia |
| Bacteria | Proteobacteria | Gammaproteobacteria | SAR86 clade | NA | NA |
| Bacteria | Verrucomicrobiota | Verrucomicrobiae | Opitutales | Puniceicoccaceae | Lentimonas |
| Bacteria | Proteobacteria | Gammaproteobacteria | Alteromonadales | Alteromonadaceae | Aliiglaciecola |
| Bacteria | Proteobacteria | Gammaproteobacteria | Alteromonadales | Pseudoalteromonadaceae | NA |
| Bacteria | Proteobacteria | Gammaproteobacteria | Alteromonadales | Pseudoalteromonadaceae | Psychrosphaera |
| Bacteria | Proteobacteria | Alphaproteobacteria | Rhodobacterales | Rhodobacteraceae | NA |
| Bacteria | Proteobacteria | Gammaproteobacteria | Alteromonadales | Psychromonadaceae | Psychromonas |
| Bacteria | Proteobacteria | Alphaproteobacteria | SAR11 clade | Clade I | Clade Ia |
| Bacteria | Actinobacteriota | Acidimicrobiia | Microtrichales | Microtrichaceae | Sva0996 marine group |
| Bacteria | Bacteroidota | Bacteroidia | Flavobacteriales | Flavobacteriaceae | NS2b marine group |
| Bacteria | Proteobacteria | Gammaproteobacteria | Burkholderiales | Nitrosomonadaceae | IS-44 |
| Bacteria | Bacteroidota | Bacteroidia | Flavobacteriales | Crocinitomicaceae | Fluviicola |
| Bacteria | Proteobacteria | Gammaproteobacteria | Cellvibrionales | Halieaceae | Pseudohaliea |
| Bacteria | Proteobacteria | Alphaproteobacteria | Rhodobacterales | Rhodobacteraceae | Pseudophaeobacter |
| Bacteria | Bacteroidota | Bacteroidia | Flavobacteriales | Flavobacteriaceae | NS4 marine group |
| Bacteria | Proteobacteria | Alphaproteobacteria | SAR11 clade | Clade IV | NA |
| Bacteria | Bacteroidota | Bacteroidia | Flavobacteriales | Flavobacteriaceae | Algitalea |
| Bacteria | Proteobacteria | Alphaproteobacteria | Rhodobacterales | Rhodobacteraceae | NA |
| Bacteria | Proteobacteria | Alphaproteobacteria | Rhodobacterales | Rhodobacteraceae | NA |
| Bacteria | Bacteroidota | Bacteroidia | Chitinophagales | Saprospiraceae | NA |
| Archaea | Crenarchaeota | Nitrososphaeria | Nitrosopumilales | Nitrosopumilaceae | Candidatus Nitrosopumilus |
| Bacteria | Proteobacteria | Alphaproteobacteria | Rhodobacterales | Rhodobacteraceae | Amylibacter |
| Bacteria | Proteobacteria | Alphaproteobacteria | Rhodobacterales | Rhodobacteraceae | Jannaschia |
| Bacteria | Proteobacteria | Gammaproteobacteria | Cellvibrionales | Halieaceae | OM60(NOR5) clade |
| Archaea | Crenarchaeota | Nitrososphaeria | Nitrosopumilales | Nitrosopumilaceae | Candidatus Nitrosopumilus |
| Bacteria | Proteobacteria | Gammaproteobacteria | Alteromonadales | Alteromonadaceae | Paraglaciecola |
| Bacteria | Bacteroidota | Bacteroidia | Flavobacteriales | NS9 marine group | NA |
| Bacteria | Proteobacteria | Alphaproteobacteria | SAR11 clade | Clade I | Clade Ia |
| Bacteria | Bacteroidota | Bacteroidia | Flavobacteriales | Flavobacteriaceae | NS5 marine group |
| Bacteria | Bacteroidota | Bacteroidia | Flavobacteriales | Flavobacteriaceae | NA |
| Bacteria | Bacteroidota | Bacteroidia | Flavobacteriales | Cryomorphaceae | NA |
| Bacteria | Bacteroidota | Bacteroidia | Flavobacteriales | Cryomorphaceae | Vicingus |
| Bacteria | Bacteroidota | Bacteroidia | Flavobacteriales | Flavobacteriaceae | NS3a marine group |
| Bacteria | Proteobacteria | Gammaproteobacteria | Alteromonadales | Pseudoalteromonadaceae | Pseudoalteromonas |
| Bacteria | Proteobacteria | Gammaproteobacteria | Oceanospirillales | Marinomonadaceae | Marinomonas |
| Bacteria | Bacteroidota | Bacteroidia | Flavobacteriales | Flavobacteriaceae | Polaribacter |
| Bacteria | Proteobacteria | Gammaproteobacteria | KI89A clade | NA | NA |
| Bacteria | Proteobacteria | Gammaproteobacteria | Burkholderiales | Methylophilaceae | OM43 clade |
| Bacteria | Proteobacteria | Gammaproteobacteria | Oceanospirillales | Nitrincolaceae | NA |
| Bacteria | Bacteroidota | Bacteroidia | Flavobacteriales | Flavobacteriaceae | Polaribacter |
| Bacteria | Proteobacteria | Alphaproteobacteria | Rhodobacterales | Rhodobacteraceae | Sulfitobacter |
| Bacteria | Proteobacteria | Alphaproteobacteria | SAR11 clade | Clade II | NA |
| Bacteria | Proteobacteria | Alphaproteobacteria | Rhodospirillales | AEGEAN-169 marine group | NA |
| Bacteria | Proteobacteria | Gammaproteobacteria | Alteromonadales | Alteromonadaceae | Alteromonas |
| Bacteria | Proteobacteria | Gammaproteobacteria | Oceanospirillales | Marinomonadaceae | Marinomonas |
| Bacteria | Bacteroidota | Bacteroidia | Flavobacteriales | Flavobacteriaceae | NS5 marine group |
| Bacteria | Proteobacteria | Alphaproteobacteria | Rhodobacterales | Rhodobacteraceae | Planktomarina |
| Bacteria | Proteobacteria | Gammaproteobacteria | SAR86 clade | NA | NA |
| Archaea | Thermoplasmatota | Thermoplasmata | Marine Group II | NA | NA |
| Bacteria | Verrucomicrobiota | Verrucomicrobiae | Verrucomicrobiales | Rubritaleaceae | Roseibacillus |
| Bacteria | Proteobacteria | Alphaproteobacteria | Caulobacterales | Hyphomonadaceae | NA |
| Bacteria | Bacteroidota | Bacteroidia | Flavobacteriales | NS9 marine group | NA |
| Bacteria | Proteobacteria | Gammaproteobacteria | Oceanospirillales | Litoricolaceae | Litoricola |
| Bacteria | Proteobacteria | Alphaproteobacteria | Defluviicoccales | NA | NA |
| Bacteria | Proteobacteria | Gammaproteobacteria | SAR86 clade | NA | NA |
| Bacteria | Nitrospinota | Nitrospinia | Nitrospinales | Nitrospinaceae | LS-NOB |
| Bacteria | Proteobacteria | Gammaproteobacteria | Alteromonadales | Alteromonadaceae | Glaciecola |
| Bacteria | Bacteroidota | Bacteroidia | Flavobacteriales | Flavobacteriaceae | Polaribacter |
| Bacteria | Bacteroidota | Bacteroidia | Flavobacteriales | Flavobacteriaceae | NS4 marine group |
| Bacteria | Bacteroidota | Bacteroidia | Flavobacteriales | NS7 marine group | NA |
| Bacteria | Bacteroidota | Bacteroidia | Flavobacteriales | Flavobacteriaceae | NS4 marine group |
| Bacteria | Bacteroidota | Bacteroidia | Flavobacteriales | Flavobacteriaceae | Polaribacter |
| Bacteria | Proteobacteria | Alphaproteobacteria | Rhodobacterales | Rhodobacteraceae | Sulfitobacter |
| Bacteria | Proteobacteria | Gammaproteobacteria | Oceanospirillales | Nitrincolaceae | NA |
| Bacteria | Proteobacteria | Alphaproteobacteria | Rhodospirillales | AEGEAN-169 marine group | NA |
| Bacteria | Bacteroidota | Bacteroidia | Sphingobacteriales | NS11-12 marine group | NA |
| Bacteria | Proteobacteria | Alphaproteobacteria | Rhodobacterales | Rhodobacteraceae | Yoonia-Loktanella |
| Bacteria | Bacteroidota | Bacteroidia | Flavobacteriales | Flavobacteriaceae | Zobellia |
| Bacteria | Bacteroidota | Bacteroidia | Flavobacteriales | Flavobacteriaceae | Formosa |
| Bacteria | Proteobacteria | Gammaproteobacteria | Cellvibrionales | Halieaceae | Luminiphilus |
| Bacteria | Planctomycetota | Planctomycetes | Pirellulales | Pirellulaceae | Rubripirellula |
| Bacteria | Proteobacteria | Alphaproteobacteria | Thalassobaculales | Nisaeaceae | OM75 clade |
| Bacteria | Bacteroidota | Bacteroidia | Flavobacteriales | Crocinitomicaceae | Fluviicola |
| Bacteria | Bacteroidota | Bacteroidia | Flavobacteriales | Flavobacteriaceae | NS2b marine group |
| Bacteria | Proteobacteria | Gammaproteobacteria | SAR86 clade | NA | NA |
| Bacteria | Bacteroidota | Bacteroidia | Flavobacteriales | Flavobacteriaceae | NS5 marine group |
| Archaea | Thermoplasmatota | Thermoplasmata | Marine Group II | NA | NA |
| Bacteria | Proteobacteria | Gammaproteobacteria | Vibrionales | Vibrionaceae | Aliivibrio |
| Bacteria | Proteobacteria | Alphaproteobacteria | Parvibaculales | NA | NA |
| Bacteria | Cyanobacteria | Cyanobacteriia | Synechococcales | Cyanobiaceae | Synechococcus CC9902 |
| Bacteria | Proteobacteria | Alphaproteobacteria | Rhodobacterales | Rhodobacteraceae | Ascidiaceihabitans |
| Bacteria | Bacteroidota | Bacteroidia | Flavobacteriales | Flavobacteriaceae | Polaribacter |
| Bacteria | Actinobacteriota | Acidimicrobiia | Actinomarinales | Actinomarinaceae | Candidatus Actinomarina |
| Bacteria | Proteobacteria | Alphaproteobacteria | SAR11 clade | Clade I | Clade Ia |
| Bacteria | Proteobacteria | Alphaproteobacteria | Rhodospirillales | AEGEAN-169 marine group | NA |
| Bacteria | Proteobacteria | Alphaproteobacteria | Puniceispirillales | SAR116 clade | Candidatus Puniceispirillum |
| Bacteria | Bacteroidota | Bacteroidia | Flavobacteriales | Flavobacteriaceae | Polaribacter |
| Bacteria | Proteobacteria | Alphaproteobacteria | SAR11 clade | Clade I | Clade Ia |
| Bacteria | Planctomycetota | Planctomycetes | Pirellulales | Pirellulaceae | Blastopirellula |
| Bacteria | Bacteroidota | Bacteroidia | Flavobacteriales | Flavobacteriaceae | NS5 marine group |
| Bacteria | Proteobacteria | Alphaproteobacteria | Caulobacterales | Hyphomonadaceae | Hellea |
| Bacteria | Proteobacteria | Alphaproteobacteria | SAR11 clade | Clade I | Clade Ia |
| Bacteria | Proteobacteria | Alphaproteobacteria | Thalassobaculales | Nisaeaceae | OM75 clade |
| Archaea | Thermoplasmatota | Thermoplasmata | Marine Group II | NA | NA |
| Bacteria | Proteobacteria | Alphaproteobacteria | SAR11 clade | Clade IV | NA |
| Bacteria | Bacteroidota | Bacteroidia | Flavobacteriales | Flavobacteriaceae | Nonlabens |
| Bacteria | Proteobacteria | Gammaproteobacteria | Cellvibrionales | Cellvibrionaceae | NA |
| Bacteria | Proteobacteria | Gammaproteobacteria | Burkholderiales | Methylophilaceae | OM43 clade |
| Bacteria | Proteobacteria | Gammaproteobacteria | Alteromonadales | Alteromonadaceae | Glaciecola |
| Bacteria | Proteobacteria | Gammaproteobacteria | SAR86 clade | NA | NA |
| Bacteria | Proteobacteria | Alphaproteobacteria | Micavibrionales | Micavibrionaceae | NA |
| Bacteria | Proteobacteria | Alphaproteobacteria | NA | NA | NA |
| Bacteria | Actinobacteriota | Acidimicrobiia | Microtrichales | Microtrichaceae | Sva0996 marine group |
| Archaea | Thermoplasmatota | Thermoplasmata | Marine Group II | NA | NA |
| Bacteria | Verrucomicrobiota | Verrucomicrobiae | Verrucomicrobiales | Rubritaleaceae | Persicirhabdus |
| Bacteria | Proteobacteria | Gammaproteobacteria | Oceanospirillales | Saccharospirillaceae | Oleispira |
| Bacteria | Bacteroidota | Bacteroidia | Chitinophagales | NA | NA |
| Bacteria | Bacteroidota | Bacteroidia | Flavobacteriales | Flavobacteriaceae | Olleya |
| Bacteria | Proteobacteria | Gammaproteobacteria | Alteromonadales | Alteromonadaceae | Aliiglaciecola |
| Bacteria | Proteobacteria | Alphaproteobacteria | Rhodospirillales | AEGEAN-169 marine group | NA |
| Archaea | Crenarchaeota | Nitrososphaeria | Nitrosopumilales | Nitrosopumilaceae | Candidatus Nitrosopumilus |
| Bacteria | Proteobacteria | Gammaproteobacteria | UBA10353 marine group | NA | NA |
| Bacteria | Proteobacteria | Alphaproteobacteria | Rhodobacterales | Rhodobacteraceae | Yoonia-Loktanella |
| Bacteria | Marinimicrobia (SAR406 clade) | NA | NA | NA | NA |
| Bacteria | Proteobacteria | Alphaproteobacteria | SAR11 clade | Clade I | Clade Ia |
| Bacteria | Bacteroidota | Bacteroidia | Chitinophagales | Saprospiraceae | Lewinella |
| Bacteria | Bacteroidota | Bacteroidia | Flavobacteriales | Flavobacteriaceae | NS4 marine group |
| Bacteria | Proteobacteria | Gammaproteobacteria | Cellvibrionales | Porticoccaceae | Porticoccus |
| **134 ASVs shared between seawater samples and drill cores from the subaerial deposit.** | | | | | |
| Bacteria | Proteobacteria | Alphaproteobacteria | SAR11 clade | Clade I | Clade Ia |
| Archaea | Thermoplasmatota | Thermoplasmata | Marine Group II | NA | NA |
| Bacteria | Proteobacteria | Gammaproteobacteria | Thiotrichales | Thiotrichaceae | NA |
| Bacteria | Bacteroidota | Bacteroidia | Flavobacteriales | NS9 marine group | NA |
| Bacteria | Planctomycetota | Planctomycetes | Pirellulales | Pirellulaceae | Rubripirellula |
| Bacteria | Proteobacteria | Alphaproteobacteria | SAR11 clade | Clade II | NA |
| Bacteria | Proteobacteria | Gammaproteobacteria | Alteromonadales | Pseudoalteromonadaceae | Pseudoalteromonas |
| Bacteria | Bacteroidota | Bacteroidia | Flavobacteriales | Flavobacteriaceae | NS5 marine group |
| Bacteria | Proteobacteria | Alphaproteobacteria | Rhodobacterales | Rhodobacteraceae | Amylibacter |
| Bacteria | Cyanobacteria | Cyanobacteriia | Synechococcales | Cyanobiaceae | Synechococcus CC9902 |
| Bacteria | Proteobacteria | Alphaproteobacteria | Rhodobacterales | Rhodobacteraceae | Litoreibacter |
| Bacteria | Proteobacteria | Alphaproteobacteria | Parvibaculales | OCS116 clade | NA |
| Bacteria | Proteobacteria | Gammaproteobacteria | Alteromonadales | Alteromonadaceae | Alteromonas |
| Bacteria | Proteobacteria | Alphaproteobacteria | SAR11 clade | Clade IV | NA |
| Bacteria | Verrucomicrobiota | Verrucomicrobiae | Verrucomicrobiales | Rubritaleaceae | Roseibacillus |
| Bacteria | Proteobacteria | Gammaproteobacteria | SAR86 clade | NA | NA |
| Bacteria | Proteobacteria | Gammaproteobacteria | Cellvibrionales | Halieaceae | OM60(NOR5) clade |
| Archaea | Crenarchaeota | Nitrososphaeria | Nitrosopumilales | Nitrosopumilaceae | Candidatus Nitrosopumilus |
| Bacteria | Actinobacteriota | Acidimicrobiia | Actinomarinales | Actinomarinaceae | Candidatus Actinomarina |
| Bacteria | Proteobacteria | Gammaproteobacteria | Oceanospirillales | Pseudohongiellaceae | Pseudohongiella |
| Bacteria | Bacteroidota | Bacteroidia | Flavobacteriales | Cryomorphaceae | NA |
| Archaea | Crenarchaeota | Nitrososphaeria | Nitrosopumilales | Nitrosopumilaceae | Candidatus Nitrosopumilus |
| Archaea | Thermoplasmatota | Thermoplasmata | Marine Group II | NA | NA |
| Bacteria | Bacteroidota | Bacteroidia | Flavobacteriales | NS9 marine group | NA |
| Bacteria | Bacteroidota | Bacteroidia | Flavobacteriales | Flavobacteriaceae | NS5 marine group |
| Bacteria | Verrucomicrobiota | Verrucomicrobiae | Opitutales | Puniceicoccaceae | Lentimonas |
| Bacteria | Bacteroidota | Bacteroidia | Flavobacteriales | Flavobacteriaceae | Formosa |
| Bacteria | Marinimicrobia (SAR406 clade) | NA | NA | NA | NA |
| Bacteria | Proteobacteria | Gammaproteobacteria | Cellvibrionales | Porticoccaceae | SAR92 clade |
| Bacteria | Verrucomicrobiota | Verrucomicrobiae | Verrucomicrobiales | Rubritaleaceae | Persicirhabdus |
| Bacteria | Actinobacteriota | Acidimicrobiia | Microtrichales | Microtrichaceae | Sva0996 marine group |
| Bacteria | Bacteroidota | Bacteroidia | Sphingobacteriales | NS11-12 marine group | NA |
| Bacteria | Proteobacteria | Gammaproteobacteria | SAR86 clade | NA | NA |
| Bacteria | Proteobacteria | Gammaproteobacteria | Thiomicrospirales | Thioglobaceae | SUP05 cluster |
| Bacteria | Proteobacteria | Alphaproteobacteria | Parvibaculales | PS1 clade | NA |
| Bacteria | Proteobacteria | Gammaproteobacteria | Ectothiorhodospirales | Ectothiorhodospiraceae | NA |
| Bacteria | Proteobacteria | Gammaproteobacteria | Vibrionales | Vibrionaceae | Aliivibrio |
| Bacteria | Planctomycetota | Phycisphaerae | Phycisphaerales | Phycisphaeraceae | CL500-3 |
| Bacteria | Cyanobacteria | Cyanobacteriia | Synechococcales | Cyanobiaceae | Synechococcus CC9902 |
| Bacteria | Proteobacteria | Alphaproteobacteria | SAR11 clade | Clade I | Clade Ia |
| Bacteria | Proteobacteria | Gammaproteobacteria | SAR86 clade | NA | NA |
| Bacteria | Verrucomicrobiota | Verrucomicrobiae | Opitutales | Puniceicoccaceae | Lentimonas |
| Bacteria | Proteobacteria | Alphaproteobacteria | SAR11 clade | Clade I | Clade Ia |
| Bacteria | Actinobacteriota | Acidimicrobiia | Microtrichales | Microtrichaceae | Sva0996 marine group |
| Bacteria | Bacteroidota | Bacteroidia | Flavobacteriales | Flavobacteriaceae | NS2b marine group |
| Bacteria | Bacteroidota | Bacteroidia | Flavobacteriales | Flavobacteriaceae | NS4 marine group |
| Archaea | Crenarchaeota | Nitrososphaeria | Nitrosopumilales | Nitrosopumilaceae | Candidatus Nitrosopumilus |
| Bacteria | Proteobacteria | Alphaproteobacteria | Rhodobacterales | Rhodobacteraceae | Amylibacter |
| Bacteria | Proteobacteria | Gammaproteobacteria | Cellvibrionales | Halieaceae | OM60(NOR5) clade |
| Bacteria | Bacteroidota | Bacteroidia | Flavobacteriales | NS9 marine group | NA |
| Bacteria | Bacteroidota | Bacteroidia | Flavobacteriales | Flavobacteriaceae | NS5 marine group |
| Bacteria | Bacteroidota | Bacteroidia | Flavobacteriales | Flavobacteriaceae | NA |
| Bacteria | Bacteroidota | Bacteroidia | Flavobacteriales | Cryomorphaceae | NA |
| Bacteria | Bacteroidota | Bacteroidia | Flavobacteriales | Cryomorphaceae | Vicingus |
| Bacteria | Proteobacteria | Gammaproteobacteria | Alteromonadales | Pseudoalteromonadaceae | Pseudoalteromonas |
| Bacteria | Proteobacteria | Gammaproteobacteria | KI89A clade | NA | NA |
| Bacteria | Proteobacteria | Gammaproteobacteria | Burkholderiales | Methylophilaceae | OM43 clade |
| Bacteria | Proteobacteria | Alphaproteobacteria | Rhodospirillales | AEGEAN-169 marine group | NA |
| Bacteria | Bacteroidota | Bacteroidia | Flavobacteriales | Flavobacteriaceae | NS5 marine group |
| Bacteria | Proteobacteria | Alphaproteobacteria | Rhodobacterales | Rhodobacteraceae | Planktomarina |
| Archaea | Thermoplasmatota | Thermoplasmata | Marine Group II | NA | NA |
| Bacteria | Verrucomicrobiota | Verrucomicrobiae | Verrucomicrobiales | Rubritaleaceae | Roseibacillus |
| Bacteria | Bacteroidota | Bacteroidia | Flavobacteriales | NS9 marine group | NA |
| Bacteria | Proteobacteria | Gammaproteobacteria | SAR86 clade | NA | NA |
| Bacteria | Bacteroidota | Bacteroidia | Flavobacteriales | Flavobacteriaceae | Polaribacter |
| Bacteria | Bacteroidota | Bacteroidia | Flavobacteriales | Flavobacteriaceae | NS4 marine group |
| Bacteria | Bacteroidota | Bacteroidia | Flavobacteriales | NS7 marine group | NA |
| Bacteria | Bacteroidota | Bacteroidia | Flavobacteriales | Flavobacteriaceae | NS4 marine group |
| Bacteria | Proteobacteria | Gammaproteobacteria | Oceanospirillales | Nitrincolaceae | NA |
| Bacteria | Proteobacteria | Alphaproteobacteria | Rhodospirillales | AEGEAN-169 marine group | NA |
| Bacteria | Bacteroidota | Bacteroidia | Flavobacteriales | Flavobacteriaceae | Formosa |
| Bacteria | Proteobacteria | Gammaproteobacteria | Cellvibrionales | Halieaceae | Luminiphilus |
| Bacteria | Planctomycetota | Planctomycetes | Pirellulales | Pirellulaceae | Rubripirellula |
| Bacteria | Proteobacteria | Alphaproteobacteria | Thalassobaculales | Nisaeaceae | OM75 clade |
| Bacteria | Bacteroidota | Bacteroidia | Flavobacteriales | Flavobacteriaceae | NS2b marine group |
| Bacteria | Proteobacteria | Gammaproteobacteria | SAR86 clade | NA | NA |
| Bacteria | Bacteroidota | Bacteroidia | Flavobacteriales | Flavobacteriaceae | NS5 marine group |
| Archaea | Thermoplasmatota | Thermoplasmata | Marine Group II | NA | NA |
| Bacteria | Proteobacteria | Gammaproteobacteria | Vibrionales | Vibrionaceae | Aliivibrio |
| Bacteria | Cyanobacteria | Cyanobacteriia | Synechococcales | Cyanobiaceae | Synechococcus CC9902 |
| Bacteria | Proteobacteria | Alphaproteobacteria | Rhodobacterales | Rhodobacteraceae | Ascidiaceihabitans |
| Bacteria | Actinobacteriota | Acidimicrobiia | Actinomarinales | Actinomarinaceae | Candidatus Actinomarina |
| Bacteria | Proteobacteria | Alphaproteobacteria | SAR11 clade | Clade I | Clade Ia |
| Bacteria | Proteobacteria | Alphaproteobacteria | Rhodospirillales | AEGEAN-169 marine group | NA |
| Bacteria | Proteobacteria | Alphaproteobacteria | SAR11 clade | Clade I | Clade Ia |
| Bacteria | Planctomycetota | Planctomycetes | Pirellulales | Pirellulaceae | Blastopirellula |
| Bacteria | Bacteroidota | Bacteroidia | Flavobacteriales | Flavobacteriaceae | NS5 marine group |
| Bacteria | Proteobacteria | Alphaproteobacteria | Caulobacterales | Hyphomonadaceae | Hellea |
| Bacteria | Proteobacteria | Alphaproteobacteria | SAR11 clade | Clade I | Clade Ia |
| Bacteria | Proteobacteria | Alphaproteobacteria | Thalassobaculales | Nisaeaceae | OM75 clade |
| Archaea | Thermoplasmatota | Thermoplasmata | Marine Group II | NA | NA |
| Bacteria | Proteobacteria | Alphaproteobacteria | SAR11 clade | Clade IV | NA |
| Bacteria | Proteobacteria | Gammaproteobacteria | Burkholderiales | Methylophilaceae | OM43 clade |
| Bacteria | Proteobacteria | Gammaproteobacteria | SAR86 clade | NA | NA |
| Bacteria | Proteobacteria | Alphaproteobacteria | NA | NA | NA |
| Bacteria | Actinobacteriota | Acidimicrobiia | Microtrichales | Microtrichaceae | Sva0996 marine group |
| Archaea | Thermoplasmatota | Thermoplasmata | Marine Group II | NA | NA |
| Bacteria | Verrucomicrobiota | Verrucomicrobiae | Verrucomicrobiales | Rubritaleaceae | Persicirhabdus |
| Bacteria | Proteobacteria | Alphaproteobacteria | Rhodospirillales | AEGEAN-169 marine group | NA |
| Bacteria | Proteobacteria | Gammaproteobacteria | UBA10353 marine group | NA | NA |
| Bacteria | Marinimicrobia (SAR406 clade) | NA | NA | NA | NA |
| Bacteria | Proteobacteria | Alphaproteobacteria | SAR11 clade | Clade I | Clade Ia |
| Bacteria | Bacteroidota | Bacteroidia | Flavobacteriales | Flavobacteriaceae | NS4 marine group |
| Bacteria | Proteobacteria | Gammaproteobacteria | Cellvibrionales | Porticoccaceae | Porticoccus |
| Bacteria | Proteobacteria | Gammaproteobacteria | OM182 clade | NA | NA |
| Bacteria | Marinimicrobia (SAR406 clade) | NA | NA | NA | NA |
| Bacteria | Bacteroidota | Bacteroidia | Flavobacteriales | Cryomorphaceae | NA |
| Bacteria | Bacteroidota | Bacteroidia | Flavobacteriales | Cryomorphaceae | NA |
| Bacteria | Proteobacteria | Alphaproteobacteria | Parvibaculales | OCS116 clade | NA |
| Bacteria | Bacteroidota | Bacteroidia | Flavobacteriales | NS9 marine group | NA |
| Archaea | Thermoplasmatota | Thermoplasmata | Marine Group II | NA | NA |
| Bacteria | Bacteroidota | Bacteroidia | Flavobacteriales | Flavobacteriaceae | Flavicella |
| Bacteria | Verrucomicrobiota | Verrucomicrobiae | Pedosphaerales | Pedosphaeraceae | SCGC AAA164-E04 |
| Bacteria | Bacteroidota | Bacteroidia | Flavobacteriales | Flavobacteriaceae | NS4 marine group |
| Bacteria | Bacteroidota | Bacteroidia | Flavobacteriales | NS9 marine group | NA |
| Bacteria | Actinobacteriota | Acidimicrobiia | Microtrichales | Microtrichaceae | Sva0996 marine group |
| Bacteria | Proteobacteria | Gammaproteobacteria | Thiomicrospirales | Thioglobaceae | SUP05 cluster |
| Bacteria | Proteobacteria | Alphaproteobacteria | SAR11 clade | Clade II | NA |
| Bacteria | Proteobacteria | Alphaproteobacteria | Rhizobiales | Stappiaceae | NA |
| Bacteria | Bacteroidota | Bacteroidia | Flavobacteriales | Cryomorphaceae | NA |
| Bacteria | Actinobacteriota | Actinobacteria | PeM15 | NA | NA |
| Bacteria | Proteobacteria | Alphaproteobacteria | Parvibaculales | Parvibaculaceae | NA |
| Bacteria | Planctomycetota | Phycisphaerae | Phycisphaerales | Phycisphaeraceae | CL500-3 |
| Archaea | Thermoplasmatota | Thermoplasmata | Marine Group III | NA | NA |
| Bacteria | Proteobacteria | Gammaproteobacteria | SAR86 clade | NA | NA |
| Bacteria | Proteobacteria | Alphaproteobacteria | Rhodospirillales | AEGEAN-169 marine group | NA |
| Bacteria | Proteobacteria | Alphaproteobacteria | Defluviicoccales | NA | NA |
| Bacteria | Marinimicrobia (SAR406 clade) | NA | NA | NA | NA |
| Bacteria | Actinobacteriota | Acidimicrobiia | Microtrichales | Microtrichaceae | Sva0996 marine group |
| Bacteria | Proteobacteria | Alphaproteobacteria | Rhodobacterales | Rhodobacteraceae | Litoreibacter |
| Archaea | Thermoplasmatota | Thermoplasmata | Marine Group II | NA | NA |
| Bacteria | Proteobacteria | Gammaproteobacteria | HOC36 | NA | NA |
| Bacteria | Proteobacteria | Alphaproteobacteria | SAR11 clade | Clade I | Clade Ia |
| Bacteria | Bacteroidota | Bacteroidia | Flavobacteriales | NS9 marine group | NA |
| **128 ASVs only shared between drilling fluid and drill cores from the submarine deposit.** | | | | | |
| Bacteria | Cyanobacteria | Cyanobacteriia | Synechococcales | Synechococcales Incertae Sedis | Schizothrix LEGE 07164 |
| Bacteria | Proteobacteria | Gammaproteobacteria | Alteromonadales | Psychromonadaceae | Psychromonas |
| Bacteria | Bacteroidota | Bacteroidia | Chitinophagales | Saprospiraceae | Lewinella |
| Bacteria | Proteobacteria | Gammaproteobacteria | Oceanospirillales | Marinomonadaceae | Marinomonas |
| Bacteria | Proteobacteria | Gammaproteobacteria | Oceanospirillales | Saccharospirillaceae | Oceaniserpentilla |
| Bacteria | Proteobacteria | Alphaproteobacteria | Rhodobacterales | Rhodobacteraceae | NA |
| Bacteria | Proteobacteria | Alphaproteobacteria | Parvibaculales | PS1 clade | NA |
| Bacteria | Proteobacteria | Gammaproteobacteria | Granulosicoccales | Granulosicoccaceae | Granulosicoccus |
| Bacteria | Bacteroidota | Bacteroidia | Flavobacteriales | Flavobacteriaceae | NS5 marine group |
| Bacteria | Verrucomicrobiota | Verrucomicrobiae | Verrucomicrobiales | Rubritaleaceae | Rubritalea |
| Bacteria | Planctomycetota | Planctomycetes | Pirellulales | Pirellulaceae | Blastopirellula |
| Bacteria | Bdellovibrionota | Bdellovibrionia | Bacteriovoracales | Bacteriovoracaceae | Peredibacter |
| Archaea | Crenarchaeota | Nitrososphaeria | Nitrosopumilales | Nitrosopumilaceae | Candidatus Nitrosopelagicus |
| Bacteria | Proteobacteria | Gammaproteobacteria | Oceanospirillales | Marinomonadaceae | Marinomonas |
| Bacteria | Patescibacteria | Gracilibacteria | NA | NA | NA |
| Bacteria | Proteobacteria | Gammaproteobacteria | Thiotrichales | Thiotrichaceae | Cocleimonas |
| Bacteria | Verrucomicrobiota | Verrucomicrobiae | Verrucomicrobiales | Rubritaleaceae | Rubritalea |
| Bacteria | Bacteroidota | Bacteroidia | Flavobacteriales | Flavobacteriaceae | Tenacibaculum |
| Bacteria | Bacteroidota | Bacteroidia | Chitinophagales | Saprospiraceae | Rubidimonas |
| Bacteria | Verrucomicrobiota | Verrucomicrobiae | Verrucomicrobiales | Rubritaleaceae | NA |
| Bacteria | Proteobacteria | Alphaproteobacteria | Caulobacterales | Hyphomonadaceae | Hellea |
| Bacteria | Proteobacteria | Gammaproteobacteria | Alteromonadales | Shewanellaceae | Psychrobium |
| Bacteria | Bacteroidota | Bacteroidia | Chitinophagales | Saprospiraceae | Rubidimonas |
| Bacteria | Proteobacteria | Gammaproteobacteria | Alteromonadales | Colwelliaceae | Colwellia |
| Bacteria | Bacteroidota | Bacteroidia | Flavobacteriales | Crocinitomicaceae | Fluviicola |
| Archaea | Crenarchaeota | Nitrososphaeria | Nitrosopumilales | Nitrosopumilaceae | Candidatus Nitrosopumilus |
| Bacteria | Proteobacteria | Gammaproteobacteria | Thiotrichales | Thiotrichaceae | Leucothrix |
| Bacteria | Patescibacteria | Gracilibacteria | NA | NA | NA |
| Bacteria | Proteobacteria | Alphaproteobacteria | Rhodobacterales | Rhodobacteraceae | Yoonia-Loktanella |
| Bacteria | Proteobacteria | Alphaproteobacteria | SAR11 clade | Clade IV | NA |
| Bacteria | Proteobacteria | Gammaproteobacteria | Alteromonadales | Alteromonadaceae | Paraglaciecola |
| Bacteria | Proteobacteria | Gammaproteobacteria | Alteromonadales | Shewanellaceae | Psychrobium |
| Bacteria | Bacteroidota | Bacteroidia | Flavobacteriales | Flavobacteriaceae | Tenacibaculum |
| Bacteria | Proteobacteria | Gammaproteobacteria | Oceanospirillales | Marinomonadaceae | Marinomonas |
| Bacteria | Proteobacteria | Alphaproteobacteria | Micavibrionales | Micavibrionaceae | NA |
| Bacteria | Proteobacteria | Gammaproteobacteria | Alteromonadales | Pseudoalteromonadaceae | Pseudoalteromonas |
| Bacteria | Proteobacteria | Gammaproteobacteria | Oceanospirillales | Kangiellaceae | NA |
| Bacteria | Bacteroidota | Bacteroidia | Chitinophagales | Saprospiraceae | NA |
| Bacteria | Proteobacteria | Gammaproteobacteria | Alteromonadales | Psychromonadaceae | Psychromonas |
| Bacteria | Bacteroidota | Bacteroidia | Chitinophagales | Saprospiraceae | NA |
| Bacteria | Proteobacteria | Gammaproteobacteria | Granulosicoccales | Granulosicoccaceae | Granulosicoccus |
| Bacteria | Proteobacteria | Gammaproteobacteria | Cellvibrionales | Cellvibrionaceae | Candidatus Endobugula |
| Bacteria | Cyanobacteria | Cyanobacteriia | Synechococcales | Synechococcales Incertae Sedis | Schizothrix LEGE 07164 |
| Bacteria | Verrucomicrobiota | Verrucomicrobiae | Verrucomicrobiales | DEV007 | NA |
| Bacteria | Verrucomicrobiota | Verrucomicrobiae | Verrucomicrobiales | Rubritaleaceae | Rubritalea |
| Bacteria | Proteobacteria | Alphaproteobacteria | Caulobacterales | Hyphomonadaceae | Hyphomonas |
| Bacteria | Proteobacteria | Gammaproteobacteria | Alteromonadales | Psychromonadaceae | Psychromonas |
| Bacteria | Proteobacteria | Gammaproteobacteria | Alteromonadales | Psychromonadaceae | Psychromonas |
| Bacteria | Proteobacteria | Gammaproteobacteria | Alteromonadales | Psychromonadaceae | Psychromonas |
| Bacteria | Proteobacteria | Gammaproteobacteria | Alteromonadales | Psychromonadaceae | Psychromonas |
| Bacteria | Bacteroidota | Bacteroidia | Flavobacteriales | Flavobacteriaceae | Tenacibaculum |
| Bacteria | Proteobacteria | Gammaproteobacteria | SAR86 clade | NA | NA |
| Bacteria | Proteobacteria | Gammaproteobacteria | Cellvibrionales | Cellvibrionaceae | NA |
| Bacteria | Proteobacteria | Gammaproteobacteria | Oceanospirillales | Marinomonadaceae | Marinomonas |
| Bacteria | Proteobacteria | Gammaproteobacteria | Alteromonadales | Psychromonadaceae | Psychromonas |
| Bacteria | Bacteroidota | Bacteroidia | Cytophagales | Cyclobacteriaceae | Marinoscillum |
| Bacteria | Bacteroidota | Bacteroidia | Flavobacteriales | Flavobacteriaceae | Aurantivirga |
| Bacteria | Bacteroidota | Bacteroidia | Chitinophagales | Saprospiraceae | NA |
| Bacteria | Proteobacteria | Alphaproteobacteria | Rhodobacterales | Rhodobacteraceae | Sedimentitalea |
| Bacteria | Bacteroidota | Bacteroidia | Flavobacteriales | Flavobacteriaceae | Winogradskyella |
| Bacteria | Deinococcota | Deinococci | Deinococcales | Trueperaceae | Truepera |
| Bacteria | Patescibacteria | Gracilibacteria | JGI 0000069-P22 | NA | NA |
| Bacteria | Bacteroidota | Bacteroidia | Chitinophagales | Saprospiraceae | NA |
| Bacteria | Bacteroidota | Bacteroidia | Flavobacteriales | Cryomorphaceae | NS10 marine group |
| Bacteria | Proteobacteria | Gammaproteobacteria | Cellvibrionales | Halieaceae | Luminiphilus |
| Bacteria | Bacteroidota | Bacteroidia | Flavobacteriales | Flavobacteriaceae | Nonlabens |
| Bacteria | Proteobacteria | Gammaproteobacteria | Oceanospirillales | Endozoicomonadaceae | Endozoicomonas |
| Bacteria | Proteobacteria | Gammaproteobacteria | Cellvibrionales | Cellvibrionaceae | Candidatus Endobugula |
| Bacteria | Bacteroidota | Bacteroidia | Flavobacteriales | Flavobacteriaceae | Cellulophaga |
| Bacteria | Bacteroidota | Bacteroidia | Flavobacteriales | Flavobacteriaceae | Algitalea |
| Bacteria | Actinobacteriota | Acidimicrobiia | Microtrichales | Microtrichaceae | Sva0996 marine group |
| Bacteria | Verrucomicrobiota | Verrucomicrobiae | Verrucomicrobiales | Rubritaleaceae | Rubritalea |
| Bacteria | Patescibacteria | Gracilibacteria | Absconditabacteriales (SR1) | NA | NA |
| Bacteria | Proteobacteria | Gammaproteobacteria | Alteromonadales | Psychromonadaceae | Psychromonas |
| Bacteria | Bacteroidota | Bacteroidia | Bacteroidales | Marinifilaceae | NA |
| Bacteria | Actinobacteriota | Acidimicrobiia | Microtrichales | Microtrichaceae | Sva0996 marine group |
| Bacteria | Proteobacteria | Gammaproteobacteria | Alteromonadales | Psychromonadaceae | Psychromonas |
| Bacteria | Verrucomicrobiota | Verrucomicrobiae | Opitutales | Puniceicoccaceae | MB11C04 marine group |
| Bacteria | Patescibacteria | Gracilibacteria | JGI 0000069-P22 | NA | NA |
| Bacteria | Bacteroidota | Bacteroidia | Chitinophagales | Saprospiraceae | Portibacter |
| Bacteria | Bacteroidota | Bacteroidia | Sphingobacteriales | NS11-12 marine group | NA |
| Bacteria | Campilobacterota | Campylobacteria | Campylobacterales | Arcobacteraceae | NA |
| Bacteria | Proteobacteria | Gammaproteobacteria | Alteromonadales | Colwelliaceae | Colwellia |
| Bacteria | Verrucomicrobiota | Verrucomicrobiae | Verrucomicrobiales | DEV007 | NA |
| Bacteria | Bacteroidota | Bacteroidia | Chitinophagales | Saprospiraceae | Rubidimonas |
| Bacteria | Bacteroidota | Bacteroidia | Flavobacteriales | Flavobacteriaceae | Algitalea |
| Bacteria | Bacteroidota | Bacteroidia | Flavobacteriales | Flavobacteriaceae | NS5 marine group |
| Bacteria | Proteobacteria | Gammaproteobacteria | Oceanospirillales | Marinomonadaceae | Marinomonas |
| Bacteria | Bacteroidota | Bacteroidia | Flavobacteriales | Flavobacteriaceae | Algitalea |
| Bacteria | Proteobacteria | Gammaproteobacteria | NA | NA | NA |
| Bacteria | Proteobacteria | Gammaproteobacteria | Alteromonadales | Psychromonadaceae | Psychromonas |
| Bacteria | Proteobacteria | Alphaproteobacteria | Rhodobacterales | Rhodobacteraceae | Leisingera |
| Bacteria | Proteobacteria | Gammaproteobacteria | Cellvibrionales | Cellvibrionaceae | Aestuariicella |
| Bacteria | Proteobacteria | Gammaproteobacteria | UBA10353 marine group | NA | NA |
| Bacteria | Proteobacteria | Gammaproteobacteria | Alteromonadales | Colwelliaceae | Colwellia |
| Bacteria | Bacteroidota | Bacteroidia | Flavobacteriales | Flavobacteriaceae | Wenyingzhuangia |
| Bacteria | Proteobacteria | Gammaproteobacteria | Alteromonadales | Alteromonadaceae | Glaciecola |
| Bacteria | Bacteroidota | Bacteroidia | Flavobacteriales | Flavobacteriaceae | Algitalea |
| Bacteria | Proteobacteria | Gammaproteobacteria | Oceanospirillales | Saccharospirillaceae | Oceaniserpentilla |
| Bacteria | Bacteroidota | Bacteroidia | Flavobacteriales | Flavobacteriaceae | Wenyingzhuangia |
| Bacteria | Proteobacteria | Alphaproteobacteria | Rhodobacterales | Rhodobacteraceae | Sulfitobacter |
| Bacteria | Marinimicrobia (SAR406 clade) | NA | NA | NA | NA |
| Bacteria | Proteobacteria | Gammaproteobacteria | NA | NA | NA |
| Bacteria | Proteobacteria | Gammaproteobacteria | Gammaproteobacteria Incertae Sedis | Unknown Family | Marinicella |
| Bacteria | Proteobacteria | Alphaproteobacteria | Rhodobacterales | Rhodobacteraceae | NA |
| Bacteria | Proteobacteria | Gammaproteobacteria | Oceanospirillales | Litoricolaceae | Litoricola |
| Bacteria | Bacteroidota | Bacteroidia | Chitinophagales | Saprospiraceae | NA |
| Bacteria | Proteobacteria | Gammaproteobacteria | Alteromonadales | Colwelliaceae | Colwellia |
| Bacteria | Proteobacteria | Gammaproteobacteria | Alteromonadales | Psychromonadaceae | Psychromonas |
| Bacteria | Bacteroidota | Bacteroidia | Flavobacteriales | Flavobacteriaceae | Ulvibacter |
| Bacteria | Verrucomicrobiota | Verrucomicrobiae | Verrucomicrobiales | Rubritaleaceae | Rubritalea |
| Bacteria | Bacteroidota | Bacteroidia | Flavobacteriales | NS9 marine group | NA |
| Bacteria | Proteobacteria | Gammaproteobacteria | Oceanospirillales | Saccharospirillaceae | Oleispira |
| Bacteria | Proteobacteria | Gammaproteobacteria | Thiotrichales | Thiotrichaceae | Leucothrix |
| Bacteria | Proteobacteria | Gammaproteobacteria | Alteromonadales | Colwelliaceae | Colwellia |
| Bacteria | Campilobacterota | Campylobacteria | Campylobacterales | NA | NA |
| Bacteria | Bacteroidota | Bacteroidia | Chitinophagales | Saprospiraceae | Aureispira |
| Bacteria | Proteobacteria | Gammaproteobacteria | Alteromonadales | Psychromonadaceae | Psychromonas |
| Bacteria | Bacteroidota | Bacteroidia | Flavobacteriales | Flavobacteriaceae | Zobellia |
| Bacteria | Campilobacterota | Campylobacteria | Campylobacterales | Arcobacteraceae | NA |
| Bacteria | Bdellovibrionota | Bdellovibrionia | Bacteriovoracales | Bacteriovoracaceae | NA |
| Bacteria | Proteobacteria | Gammaproteobacteria | Alteromonadales | Alteromonadaceae | Glaciecola |
| Bacteria | Proteobacteria | Gammaproteobacteria | Alteromonadales | Colwelliaceae | Thalassotalea |
| Bacteria | Proteobacteria | Alphaproteobacteria | Caulobacterales | Hyphomonadaceae | Hyphomonas |
| Bacteria | Proteobacteria | Gammaproteobacteria | Oceanospirillales | Marinomonadaceae | Marinomonas |
| Bacteria | Cyanobacteria | Cyanobacteriia | Synechococcales | Cyanobiaceae | Synechococcus CC9902 |
| Bacteria | Proteobacteria | Alphaproteobacteria | Rhodobacterales | Rhodobacteraceae | Pontivivens |
| Bacteria | Desulfobacterota | Desulfuromonadia | PB19 | NA | NA |

**Table S7.** Number of ASVs per library and estimated alpha diversit (Shannon and InvSimpson) for universal and archaeal primer datasets. n. d., not detected.

| Library# | Bacterial 16S rRNA encoding gene tag sequencing universal primer set (515F/806R) | | | Archaeal 16S rRNA encoding gene tag sequencing Second round nested PCR (Parch340F/Arch958VR) | | |
| --- | --- | --- | --- | --- | --- | --- |
|  | Number of ASVs | Shannon | InvSimpson | Number of ASVs | Shannon | InvSimpson |
| Sur161 | 141 | 3.691361 | 19.486844 | 41 | 3.19228141 | 17.891619 |
| Sur162 | 116 | 3.538613 | 18.290608 | 34 | 2.99325049 | 14.346385 |
| Sur163 | 107 | 3.734251 | 20.626616 | n.d. | n.d. | n.d. |
| Sur164 | 61 | 3.790214 | 34.188694 | 3 | 1.08419186 | 2.91495 |
| Sur165 | 75 | 3.463842 | 13.736494 | n.d. | n.d. | n.d. |
| Sur166 | 85 | 3.894085 | 31.502765 | 8 | 1.98946606 | 6.906769 |
| Sur167 | 92 | 2.913368 | 7.898311 | 16 | 2.49383456 | 9.72973 |
| Sur168 | 41 | 2.484037 | 7.171008 | n.d. | n.d. | n.d. |
| Sur169 | 52 | 3.384146 | 18.633876 | n.d. | n.d. | n.d. |
| Sur1610 | 129 | 3.82792 | 23.577461 | 24 | 2.71981336 | 12.293442 |
| Sur16Mix | 179 | 3.655867 | 14.003799 | 22 | 2.55954284 | 10.375009 |
| Sur171 | 226 | 4.200181 | 34.840567 | n.d. | n.d. | n.d. |
| Sur172 | 84 | 4.025106 | 34.79125 | n.d. | n.d. | n.d. |
| Sur173 | 92 | 4.149951 | 38.969828 | n.d. | n.d. | n.d. |
| Sur174 | 214 | 5.103442 | 126.57596 | n.d. | n.d. | n.d. |
| Sur175 | 95 | 4.031399 | 32.710562 | n.d. | n.d. | n.d. |
| Sur176 | 234 | 4.401516 | 43.181551 | n.d. | n.d. | n.d. |
| Sur177 | 134 | 3.236111 | 10.07773 | n.d. | n.d. | n.d. |
| C4b | 209 | 4.21608 | 29.424767 | n.d. | n.d. | n.d. |
| C9b | 164 | 3.253715 | 15.570477 | n.d. | n.d. | n.d. |
| C13b | 158 | 3.975265 | 21.641024 | 13 | 1.68236435 | 4.012734 |
| C17b | 215 | 4.856728 | 63.184916 | n.d. | n.d. | n.d. |
| C22b | 86 | 3.502664 | 14.140547 | 11 | 1.35240939 | 3.151849 |
| C27b | 117 | 3.664283 | 15.80584 | 26 | 1.77531764 | 4.099265 |
| C33b | 162 | 3.504293 | 7.858449 | n.d. | n.d. | n.d. |
| C36b | 156 | 3.978891 | 16.512563 | 18 | 1.10685151 | 2.117929 |
| C39b | 210 | 4.80093 | 55.511111 | n.d. | n.d. | n.d. |
| C42b | 217 | 4.254007 | 20.210364 | 26 | 2.31866692 | 7.207853 |
| C45b | 198 | 4.673861 | 59.395709 | 7 | 0.04251626 | 1.012963 |
| C49b | 170 | 3.119454 | 8.125041 | 13 | 1.27161611 | 2.489667 |
| C52b | 177 | 4.493878 | 54.24832 | 32 | 2.9859752 | 15.824776 |
| C55b | 138 | 3.927837 | 16.836918 | 12 | 1.2796316 | 2.434565 |
| C59b | 206 | 3.195123 | 5.789091 | n.d. | n.d. | n.d. |
| C62b | 130 | 3.886429 | 19.123428 | n.d. | n.d. | n.d. |
| C65b | 206 | 4.340737 | 26.853872 | 26 | 2.42682311 | 7.648331 |
| Fum171 | 180 | 4.518433 | 53.7684 | n.d. | n.d. | n.d. |
| Fum172 | 236 | 5.042729 | 111.418251 | n.d. | n.d. | n.d. |
| SW10 | 195 | 4.578192 | 51.776402 | n.d. | n.d. | n.d. |
| SW20 | 218 | 4.662919 | 55.778984 | n.d. | n.d. | n.d. |
| SW30 | 270 | 5.063104 | 96.651064 | n.d. | n.d. | n.d. |
| SW50 | 317 | 5.143753 | 90.916747 | n.d. | n.d. | n.d. |

# Code

library(phyloseq)

library(ggplot2)

library(decontam)

library(ape)

library(dada2)

library(vegan)

library(scales)

library(tidyverse)

library(ggsignif)

library(multcomp)

library(pairwiseAdonis)

library(readr)

library(dplyr)

library(venneuler)

library(VennDiagram)

library(grid)

library(digest)

library(reshape2)

library(stringr)

library(RColorBrewer)

library(IRanges)

library(DESeq2)

library(ggpubr)

## Figure 2

raw.quad.venn(

area1 = 532,

area2 = 903,

area3 = 2028,

area4 = 491,

n12 = 202,

n13 = 366,

n14 = 199,

n23 = 336,

n24 = 134,

n34 = 206,

n123 = 177,

n124 = 104,

n134 = 154,

n234 = 107,

n1234 = 93,

category = c("Drilling fluid", "Drill cores in subaerial deposit", "Drill cores in submarine deposit", "Seawater"),

fill = c("#666666", "#E6AB02", "#A6761D", "#197EC099"),

cex = 2,

cat.cex = 2,

)

## Figure 3a

ps_alpha_div <- plot_richness(ps3, x = "SampleType", color = "SampleType", measures = c("Observed", "Shannon")) + geom_boxplot() + geom_point() + scale_color_manual(values = c("Borehole_fluid" = "#66A61E", "Drill_core" = "#E6AB02", "Fumarole" = "#D95F02", "Seawater" = "#197EC099")) + theme(text = element_text(size=16)) + geom_signif(comparisons = list(c("Seawater", "Borehole_fluid"), c("Seawater", "Fumarole"), c("Borehole_fluid", "Drill_core"), c("Drill_core", "Seawater"), c("Borehole_fluid", "Fumarole"), c("Drill_core", "Fumarole")), map_signif_level = TRUE) + ylab("Observed ASVs")

ps_alpha_div

**ANOVA**

alpha.diversity <- estimate_richness(ps3, measures = c("Observed", "Shannon", "InvSimpson"))

metadat <- cbind(sample_data(ps3), alpha.diversity)

anova_result.o.ST <- aov(Observed ~ SampleType, metadat)

anova_result.s.ST <- aov(Shannon ~ SampleType, metadat)

**Tukey's HSD test**

alpha_est <- estimate_richness(ps3, measures = "Observed")

SampleType <- dplyr::pull(sample_data(ps3), SampleType)

alpha_est <- alpha_est %>%

add_column(SampleType) %>%

mutate(SampleType = as.factor(SampleType))

group_by(alpha_est, SampleType) %>%

summarise(mean = mean(Observed, na.rm = TRUE),

sd = sd(Observed, na.rm = TRUE))

res_aov <- aov(Observed ~ SampleType, alpha_est)

post_test <- glht(res_aov,

linfct = mcp(SampleType= "Tukey"))

## Figure 3b

ps_norm <- rarefy_even_depth(ps3, sample.size = 2000, trimOTUs = TRUE)

ord.NMDS = ordinate(ps_norm, "NMDS", "bray") # stress 0.180

pNMDS = plot_ordination(ps_norm, ord.NMDS, type="samples", title="NMDS Bray", color="SampleType", label="Samples") + geom_point(size=5) + theme_bw() + theme(text = element_text(size=20)) + scale_color_manual(values = c("Borehole_fluid" = "#66A61E", "Drill_core" = "#E6AB02", "Fumarole" = "#D95F02", "Seawater" = "#197EC099")) + stat_ellipse(geom = "polygon", alpha=0, type = "t", linetype=3)

**PERMANOVA**

dist_bray <- distance(ps_norm, method = "bray")

dist_bray_matrix <- as.matrix(dist_bray)

adonis(dist_bray_matrix ~ SampleType, permutations = 999)

res <- pairwise.adonis(dist_bray_matrix, SampleType)

## Figure 3c

ps_rel <- transform_sample_counts(ps3, function(x) x / sum(x))

mdf.ps = psmelt(ps_rel) %>% tbl_df

phyla = mdf.ps %>%

group_by(SampleType, Phylum) %>%

summarize(Abundance = sum(Abundance)) %>%

group_by(Phylum) %>%

summarize(m = mean(Abundance)) %>%

arrange(desc(m))

N = 60

topN = mdf.ps %>%

group_by(SampleType, Sample, Phylum) %>%

summarize(Abundance = sum(Abundance)) %>%

group_by(Phylum) %>%

summarize(m = mean(Abundance)) %>%

top_n(N) %>%

arrange(desc(m)) %>%

magrittr::extract2("Phylum")

mdf.other = mdf.ps %>%

mutate(Phylum.other = ifelse(Phylum %in% topN, as.character(Phylum), "Other")) %>%

group_by(SampleType,Sample, Phylum.other) %>%

summarize(Abundance = sum(Abundance))

mdf.summary = mdf.other %>%

group_by(SampleType, Phylum.other) %>%

summarise(avg = mean(Abundance),

sd = sd(Abundance),

sem = sd(Abundance)/sqrt(length(Abundance)),

proportion = round(avg*100,2)) %>%

arrange(desc(avg))

write.csv(mdf.summary, file = "Abundance calculation for SampleTypes phyla.csv")

The previous code was used to calculate relative abundances at the phylum level function of the sample type and percentages were imported into excel to make the plot.

## Figure 3d

MyVenn <- venneuler(c(A = 367, B = 491, C = 2242, D = 1415, "A&B" = 0, "A&C" = 50, "A&D" = 59, "B&C" =102, "B&D" = 27, "C&D" =122, "A&B&C"= 0, "A&B&D"=0, "A&C&D"=23, "B&C&D"=8, "A&B&C&D"=0))

plot(MyVenn)

## Figure 4.

ps6.DC <- subset_samples(ps3, SampleType == "Drill_core")

ps6.DC_rel <- transform_sample_counts(ps6.DC, function(x) x / sum(x))

ps6.DC_rel.glom.Class <- tax_glom(ps6.DC_rel, taxrank = "Class", NArm = TRUE)

dat.DC.Class <- psmelt(ps6.DC_rel.glom.Class)

dat.DC.Class$Class <- as.character(dat.DC.Class$Class)

medians.st.Class <- ddply(dat.DC.Class, ~Class, function(x) c(median=median(x$Abundance)))

Others <- medians.st.Class[medians.st.Class$median <= 0.005,]$Class

dat.DC.Class[dat.DC.Class$Class %in% Others,]$Class <- "Others"

dat.DC.Class <- within(dat.DC.Class, Sample <- factor(Sample, levels = c("C65b","C62b", "C59b", "C55b", "C52b", "C49b", "C45b", "C42b", "C39b", "C36b", "C33b", "C27b", "C22b", "C17b", "C13b", "C9b", "C4b")))

dat.DC.Class <- within(dat.DC.Class, Class <- factor(Class, levels = c("Alphaproteobacteria", "Gammaproteobacteria", "Actinobacteria", "Bacteroidia", "Bacilli", "Planctomycetes", "Verrucomicrobiae", "Clostridia", "Thermoleophilia", "Blastocatellia", "Desulfuromonadia",

"Cyanobacteriia", "Acidimicrobiia", "Bdellovibrionia", "Polyangia",

"Anaerolineae", "Deinococci", "Campylobacteria", "Thermaerobacteria", "Others")))

palMB <- c("#F1B6A1", "#D4A52A", "#E3E5DB", "#A5CFCC", "#0E899F", "#A83860", "#ED91BC", "#DB5339", "#F58851", "#42465C", "#1E479A", "#F7CDA4", "#CF529C", "#11638C")

plot.rel.DC.Class <- ggplot(dat.DC.Class, aes(x = Sample, y = Abundance, fill = Class)) + geom_bar(stat="identity") + labs(x = "SampleID", y = "Rel. abundance Class level [%]") + theme(axis.ticks = element_blank(), axis.text.x = element_text(size = 8, colour = "black", vjust = 0.1, angle = 0), axis.text.y = element_text(size = 10, colour = "black"), aspect.ratio = 1.5) + theme(panel.grid.major = element_blank(), panel.grid.minor = element_blank(), panel.background = element_blank()) + scale_y_continuous(labels = c("0", "25", "50", "75", "100"), expand = c(0,0)) + ggtitle("Class level Taxonomy by Sample") + scale_fill_manual(values=palMB) + coord_flip()

plot.rel.DC.Class

## Figure 5.

DESeq2 approach with a False Discovery Rate (FDR) cutoff of 0.01. ASVs showing a p-value < 0.01 were considered indicative of statistical significance and were manually extracted from the otu_table (vlookup function in excel) for the construction of the bubble plot.

ds = phyloseq_to_deseq2(ps3, ~ SampleType)

# or ds = phyloseq_to_deseq2(ps6.DC, ~ Category)

ds = DESeq(ds, sfType="poscounts")

alpha = 0.01

Combinations of two by two comparison among sample types:

"Borehole_fluid", "Fumarole"

"Borehole_fluid", "Drill_core"

"Borehole_fluid", "Seawater"

"Fumarole", "Drill_core"

"Fumarole", "Seawater"

"Drill_core", "Seawater"

Combinations of two by two comparison among categories:

"Category_DC_1_vs_DC_2"

"Category_DC_1_vs_DC_3"

"Category_DC_1_vs_DC_4"

"Category_DC_2_vs_DC_3"

"Category_DC_2_vs_DC_4"

"Category_DC_3_vs_DC_4"

Example:

res = results(ds, contrast=c("SampleType", "Drill_core", "Seawater"), alpha=alpha)

res = res[order(res$padj, na.last=NA), ]

res_sig = res[(res$padj < alpha), ]

res_sig = cbind(as(res_sig, "data.frame"), as(tax_table(ps3)[rownames(res_sig), ], "matrix"))

write.csv(res_sig, file = "Enrich ASVs DC vs SW 0.01.csv")

Significantly enriched ASVs identified by DESeq2 approach were extracted from the original out_table (with relative abundance) to build the bubble plot.

The following code is from <https://github.com/alex-bagnoud/OTU-table-to-bubble-plot>.

1. Import variables

otu_tab_file <- "~/R/Analyses 2021/Bacteria/DESeq2/otu_tab.txt"

tax_file <- "~/R/Analyses 2021/Bacteria/DESeq2/tax_tab.txt"

category_list <- c("F", "BF", "DC_1", "DC_2", "DC_3", "DC_4", "SW")

tax_aggr <- "Genus"

tax_number <- 100

tax_col <- "Phylum"

file_name <- "bubble_plot.svg"

plot_dim <- c(6,6)

2. Import files

otu_tab <- read.table(otu_tab_file, header = TRUE, comment.char = "", sep = "\t")

names(otu_tab)[1] <- "OTU"

otu_tab[1:4,1:4]

tax_tab <- read.table(tax_file, header = TRUE, comment.char = "", sep = "\t", fill = TRUE)

names(tax_tab)[1] <- "OTU"

tax_tab[1:5,1:7]

3. Parse the taxonomic file

for (col in 2:ncol(tax_tab)) {

for (row in 1:nrow(tax_tab)) {

if (grepl("uncultured",tax_tab[row,col],ignore.case = TRUE)) {

tax_tab[row,col] <- ""

}

if (grepl("unknown",tax_tab[row,col],ignore.case = TRUE)) {

tax_tab[row,col] <- ""

}

}

}

tax_tab2 <- as.data.frame(apply(tax_tab, 2, function(x) gsub("^$|^ $", NA, x)))

col_to_remove <- c()

for (col in 2:ncol(tax_tab2)) {

x <- sum(is.na(tax_tab2[,col]))/nrow(tax_tab2)

if (x == 1) {

col_to_remove <- c(col_to_remove, col)

}

}

if (length(col_to_remove) > 0) {

tax_tab3 <- tax_tab2[,-col_to_remove]

} else {

tax_tab3 <- tax_tab2

}

for (col in 2:ncol(tax_tab3)) {

tax_tab3[,col] <- as.character(tax_tab3[,col])

}

for (col in 2:ncol(tax_tab3)) {

for (row in 1:nrow(tax_tab3)) {

if (is.na(tax_tab3[row,col])) {

if (!grepl("OTU", tax_tab3[row,col-1]) & !grepl("unassigned", tax_tab3[row,col-1])) {

tax_tab3[row,col] <- paste0("unassigned ", tax_tab3[row,col-1])

} else {

tax_tab3[row,col] <- tax_tab3[row,col-1]

}

}

}

}

4. Compute the relative abundance of OTUs for each sample

otu_counts <- colSums(otu_tab[,-1])

otu_tab2 <- otu_tab

otu_tab2[,-1] <- sweep(otu_tab[,-1], 2, otu_counts, `/`)

otu_tab2[is.na(otu_tab2)] <- 0

colSums(otu_tab2[,-1])

5. Merge the OTU and taxonomic tables together

m <- merge(otu_tab, tax_tab3)

dim(m)

6. Aggregate the table to taxonomic level defined in the variable 'tax_aggr'

taxonomy <- c()

for (row in 1:nrow(m)) {

taxonomy <- c(taxonomy, paste0(m[row,names(m)==tax_col], ";", m[row,names(m)==tax_aggr]))

}

m2 <- m[,names(m) %in% category_list]

m3 <- aggregate(m2, by=list(taxonomy), FUN=sum)

dim(m3)

m3[1:5,1:4]

7. Sort the table by decreasing size of taxonomic groups

if (tax_number > nrow(m3)) {

tax_number <- nrow(m3)

}

m3$average <- rowMeans(m3[,-1])

m3.sorted <- m3[order(-m3$average),]

m3.sorted$selection <- rep("discarded", nrow(m3.sorted))

m3.sorted$selection[1:tax_number] <- "retained"

m3.sorted$Group.1[m3.sorted$selection == "discarded"] <- "Other;Other"

m3.sorted$average <- NULL

m3.sorted$selection <- NULL

m4 <- aggregate(m3.sorted[,-1], by=list(taxonomy=m3.sorted$Group.1), FUN=sum)

m4[m4$taxonomy == "Other;Other", -1]

mean(as.numeric(m4[m4$taxonomy == "Other;Other", -1]))

8. Transpose 'm4'

n <- m4$taxonomy

m4.t <- as.data.frame(t(m4[,-1]))

colnames(m4.t) <- n

m4.t$sample <- rownames(m4.t)

rownames(m4.t) <- NULL

9. Melt and merge the two dataframes

molten.mean <- melt(m4.t, id.vars = "sample")

molten.mean$id <- paste0(molten.mean$sample, "-", molten.mean$variable)

molten.sd <- melt(m4.t, id.vars = "sample")

molten.sd$id <- paste0(molten.sd$sample, "-", molten.sd$variable)

molten <- merge(molten.mean, molten.sd, by.x = "id", by.y = "id")

10. Final rearragement of the dataframe

molten$id <- NULL

molten$sample.y <- NULL

molten$variable.y <- NULL

names(molten) <- c("sample", "taxonomy", "mean", "sd")

molten$tax_col <- str_split_fixed(molten$taxonomy, ";", 2)[,1]

molten$tax_bin <- str_split_fixed(molten$taxonomy, ";", 2)[,2]

molten <- molten[order(molten$tax_col),]

tax_levels <- as.character(molten$tax_bin[!duplicated(molten$tax_bin)])

tax_levels <- tax_levels[tax_levels != "Other"]

tax_levels <- c(tax_levels, "Other")

molten$tax_bin <- factor(molten$tax_bin, levels = rev(tax_levels))

molten$sample <- factor(molten$sample, levels = category_list)

molten2 <- molten[molten$mean > 0,]

molten3 <- molten2[molten2$tax_col !="Other",]

11. Compute the bubble plot

bubble_plot <- ggplot(molten3,aes(sample,tax_bin)) +

geom_point(aes(size=mean, fill=tax_col),shape=21,color="black") +

theme(panel.grid.major=element_line(linetype=1,color="grey"),

axis.text.x=element_text(angle=30,hjust=1,vjust=1, size = 10),

panel.background = element_blank()) +

ylab("Taxonomic bins") +

xlab("Category") +

scale_fill_brewer(palette="Set2", name="Taxonomic\nclade") +

scale_fill_discrete(name="Taxonomic\nclade") +

scale_fill_manual(values= c(brewer.pal(12, "Set2"), brewer.pal(12, "Set3")), name="Taxonomic\nclade") +

scale_size(name = "Relative\nabundance")

+ coord_flip()

bubble_plot

## Figure S1.

ps.pa <- transform_sample_counts(ps, function(abund) 1*(abund>0))

ps.pa.neg <- prune_samples(sample_data(ps.pa)$Sample_or_Control == "Control Sample", ps.pa)

ps.pa.pos <- prune_samples(sample_data(ps.pa)$Sample_or_Control == "True Sample", ps.pa)

df.pa <- data.frame(pa.pos=taxa_sums(ps.pa.pos), pa.neg=taxa_sums(ps.pa.neg), contaminant=contamdf.prev$contaminant)

ggplot(data=df.pa, aes(x=pa.neg, y=pa.pos, color=contaminant)) + geom_point() + xlab("Prevalence (Negative Controls)") + ylab("Prevalence (True Samples)")

## Figure S2.

library("ggpubr")

Depth <- ggscatter(my_data, x = "Depth", y = "DNA",

add = "reg.line", conf.int = TRUE,

cor.coef = TRUE, cor.method = "pearson",

xlab = "DNA concentration (ng/µL)", ylab = "Depth (m b.s)")

Temperature <- ggscatter(my_data, x = "Temperature", y = "DNA",

add = "reg.line", conf.int = TRUE,

cor.coef = TRUE, cor.method = "pearson",

xlab = "DNA concentration (ng/µL)", ylab = "Temperature (C°)")

## Figure S3.

ps2_deconta_DF <- subset_samples(ps2_deconta, SampleType == "Drilling_fluid")

DF <- plot_bar(ps2_deconta_DF)

## Figure S4.

ps2_deconta_wo_BF_C_F_rel <- transform_sample_counts(ps2_deconta_wo_BF_C_F, function(x) x / sum(x))

ps2deconta.ord <- ordinate(ps2_deconta_wo_BF_C_F_rel, "NMDS", "bray")

p3 = plot_ordination(ps2_deconta_wo_BF_C_F_rel, ps2deconta.ord, type="samples", title="NMDS Bray", color="SampleType", label = "Samples") + geom_point(size=5) + theme_bw() + theme(text = element_text(size=20)) + scale_color_manual(values = c("Borehole_fluid" = "#66A61E", "Drill_core" = "#E6AB02", "Drilling_fluid" = "#666666", "Fumarole" = "#D95F02", "Seawater" = "#197EC099", "Control_extraction" = "black"))

## Figure S5.

rarefy {vegan}

rare <- rarecurve(t(otu_table(ps2)), xlab = "Sequence sample size", ylab = "Numbers of ASVs", step=50, cex=0.5)

## Figure S6.

ps6.DC_norm <- rarefy_even_depth(ps6.DC, sample.size = 3500, trimOTUs = TRUE)

otutable <- vegan_otu(ps6.DC_norm)

sampledf <- data.frame(sample_data(ps6.DC_norm))

vegan_otu <- function(ps6.DC_norm) {

OTU <- otu_table(ps6.DC_norm)

if (taxa_are_rows(OTU)) {

OTU <- t(OTU)

}

return(as(OTU, "matrix"))

}

dca <- decorana(otutable)

summary(dca)

df <- data.frame(sampledf)

ord_all <- cca(otutable ~ Depth + Temperature, data=df)

anova(ord_all)

fit <- envfit(ord_all, df, perm = 999, display = "lc", scaling = "sites")

fit$vectors

spp.scrs <- as.data.frame(scores(fit, display = "vectors"))

spp.scrs

pval <- fit$vectors$pvals

pval

fdat <- cbind(spp.scrs, Vector = rownames(spp.scrs), pval)

bestEnvVariables<-rownames(fdat)[fdat$pval<=0.05]

eval(parse(text=paste("ord1 <- cca(otutable ~ ",do.call(paste,c(as.list(bestEnvVariables),sep=" + ")),",data=sampledf)",sep="")))

summary(ord1)

fit1 <- envfit(ord1,sampledf[,bestEnvVariables], perm = 999, display = "lc", scaling = "sites")

spp.scrs <- data.frame(scores(fit1, display = "vectors"))

pval <- fit1$vectors$pvals

spp.scrs <- cbind(spp.scrs, Vector = rownames(spp.scrs), pval) # vector table

scrs <- as.data.frame(scores(ord1, display = "sites")) # sample table

scrs <- cbind(scrs, sampledf)

spp.scrs1<- subset(spp.scrs, pval<=0.05)

p <- ggplot(scrs) +

geom_point(mapping = aes(x = CCA1, y = CCA2, colour = Category), alpha = 0.8, size = 5) +

geom_segment(data = spp.scrs1,

aes(x = 0, xend = CCA1*2.5, y = 0, yend = CCA2*2.5),

arrow = arrow(length = unit(0.25, "cm")), colour = "black", size=1) +

geom_text(data = spp.scrs1, aes(x = CCA1*3, y = CCA2*3, label = Vector), size = 4) +

theme_bw() +

theme(axis.title=element_text(size=14), axis.text=element_text(size=14), legend.text=element_text(size=14)) +

theme(legend.position="right")
